# De Novo Powered Air-Purifying Respirator Design and Fabrication for Pandemic Response

**DOI:** 10.3389/fbioe.2021.690905

**Published:** 2021-09-06

**Authors:** Akshay Kothakonda, Lyla Atta, Deborah Plana, Ferrous Ward, Chris Davis, Avilash Cramer, Robert Moran, Jacob Freake, Enze Tian, Ofer Mazor, Pavel Gorelik, Christopher Van, Christopher Hansen, Helen Yang, Yao Li, Michael S. Sinha, Ju Li, Sherry H. Yu, Nicole R. LeBoeuf, Peter K. Sorger

**Affiliations:** ^1^ Greater Boston Pandemic Fabrication Team (PanFab) c/o Harvard-MIT Center for Regulatory Science, Harvard Medical School, Boston, MA, United States; ^2^ Department of Aeronautics and Astronautics, MIT, Cambridge, MA, United States; ^3^ Department of Biological Engineering, Johns Hopkins University School of Medicine, Baltimore, MD, United States; ^4^ Harvard Ludwig Cancer Research Center and Department of Systems Biology, Harvard Medical School, Boston, MA, United States; ^5^ Harvard-MIT Division of Health Sciences and Technology, Cambridge, MA, United States; ^6^ GenOne Technologies, Cambridge, MA, United States; ^7^ Mine Survival Inc., Panama City Beach, FL, United States; ^8^ Fikst Product Development, Woburn, MA, United States; ^9^ Beijing Key Laboratory of Indoor Air Quality Evaluation and Control, Department of Building Science, Tsinghua University, Beijing, China; ^10^ Research Instrumentation Core Facility, Harvard Medical School, Boston, MA, United States; ^11^ Borobot, Middleborough, MA, United States; ^12^ Harvard Graduate School of Design, Cambridge, MA, United States; ^13^ Harvard-MIT Center for Regulatory Science, Harvard Medical School, Boston MA, United States; ^14^ Department of Nuclear Science and Engineering and Department of Materials Science and Engineering, MIT, Cambridge, MA, United States; ^15^ Department of Dermatology, Yale School of Medicine, New Haven, CT, United States; ^16^ Department of Dermatology, Center for Cutaneous Oncology, Brigham and Women’s Hospital and Dana-Farber Cancer Institute, Boston, MA, United States

**Keywords:** COVID-19, pandemic response, 3D-printing, powered air-purifying respirators, personal protective equipment, open source product development, injection molding, medical device design

## Abstract

The rapid spread of COVID-19 and disruption of normal supply chains has resulted in severe shortages of personal protective equipment (PPE), particularly devices with few suppliers such as powered air-purifying respirators (PAPRs). A scarcity of information describing design and performance criteria for PAPRs represents a substantial barrier to mitigating shortages. We sought to apply open-source product development (OSPD) to PAPRs to enable alternative sources of supply and further innovation. We describe the design, prototyping, validation, and user testing of locally manufactured, modular, PAPR components, including filter cartridges and blower units, developed by the Greater Boston Pandemic Fabrication Team (PanFab). Two designs, one with a fully custom-made filter and blower unit housing, and the other with commercially available variants (the “Custom” and “Commercial” designs, respectively) were developed; the components in the Custom design are interchangeable with those in Commercial design, although the form factor differs. The engineering performance of the prototypes was measured and safety validated using National Institutes for Occupational Safety and Health (NIOSH)-equivalent tests on apparatus available under pandemic conditions at university laboratories. Feedback was obtained from four individuals; two clinicians working in ambulatory clinical care and two research technical staff for whom PAPR use is standard occupational PPE; these individuals were asked to compare PanFab prototypes to commercial PAPRs from the perspective of usability and suggest areas for improvement. Respondents rated the PanFab Custom PAPR a 4 to 5 on a 5 Likert-scale 1) as compared to current PPE options, 2) for the sense of security with use in a clinical setting, and 3) for comfort compared to standard, commercially available PAPRs. The three other versions of the designs (with a Commercial blower unit, filter, or both) performed favorably, with survey responses consisting of scores ranging from 3 to 5. Engineering testing and clinical feedback demonstrate that the PanFab designs represent favorable alternatives to traditional PAPRs in terms of user comfort, mobility, and sense of security. A nonrestrictive license promotes innovation in respiratory protection for current and future medical emergencies.

## Introduction

The rapid and global spread of COVID-19 has led to dramatic increases in demand for personal protective equipment (PPE) for healthcare workers as well as significant disruption of supply chains and distribution networks for these products. As a consequence, the availability of high-quality respiratory protection has been problematic, causing healthcare institutions to reuse normally disposable filtering facepiece respirators (FFRs; N95-type masks) and turn to non-traditional devices as substitutes (Hack the Pandemic, 2020; Thingiverse.com, 2020; COVID-19 Response, 2020; Get Us PPE, 2021; Open Mask, 2021). Non-traditional supply chains commonly involve community-based collaborations among independent engineers, scientists, hobbyists, and volunteers in partnership with healthcare and academic institutions (McCue, 2020; Westervelt, 2020; NIH 3D Print Exchange, 2020; Sinha et al., 2020; Clark, 2020). While multiple non-traditional designs for simple PPE products such as face shields have emerged (Mostaghimi et al., 2020), and multiple commercial and non-traditional technologies have been developed to decontaminate or reuse N95-type masks (N95 Decon, 2020; McAvoy et al., 2021), few alternative sources of supply exist for more complex products. This is particularly true of powered air-purifying respirators (PAPRs) which can be worn by individuals unable to fit N95-type masks, are more comfortable in many settings, and also provide a higher level of respiratory protection. Ongoing efforts to increase the supply of PAPRs have largely involved large manufacturing companies with government support (e.g., the 3M Ford Limited-Use Public Health Emergency PAPR) (CDC, 2020a; Limited-Use Public Health Emergency PAPR, 2021).

PAPRs typically cover the entire head with a loose-fitting headpiece or hood and provide a continuous supply of filtered air to a user from a blower worn on a belt or backpack. Like N95 masks, PAPRs are used in both healthcare and industrial settings, but under non-pandemic conditions, healthcare use of PAPRs is typically limited to situations in which a healthcare worker (HCW) is unable to wear a disposable N95 mask yet must care for a patient with a suspected or confirmed airborne infection, such as tuberculosis (Institute of Medicine, 2015). Common reasons for being unable to wear a N95 mask are the presence of facial hair and poor mask fit for individuals with small or narrow faces; the latter is often revealed by qualitative fit tests routinely performed on HCWs (Occupational Safety and Health Administration, 2021). It is estimated that ∼10% of HCWs fail fit testing (Institute of Medicine, 2015), and for these individuals PAPRs are the best, and in some instances the only, alternative form of respiratory protection. N95 masks are often reused in pandemic conditions due to supply shortages, leading to concerns about further loss of fit after multiple don-doff cycles (Bergman et al., 2012).

In addition, HCWs report that PAPRs are more comfortable than masks in situations in which continuous respiratory protection is required for many hours, especially for those who have respiratory symptoms under normal circumstances or who work in hot conditions. It has also been observed that many healthcare workers who must wear N95-type masks day after day (e.g., during a sustained pandemic) experience painful abrasions (Gheisari et al., 2020; Lan et al., 2020). Attempts to mitigate discomfort by using creams, tapes, or loosening the straps that hold masks against a user’s face can decrease respiratory protection (Bui et al., 2021). PAPRs overcome this problem and provide better protection: commercial PAPRs certified by the National Institutes for Occupational Safety and Health (NIOSH) offer higher filtration efficiency as compared to N95 masks (99.97 vs. 95%) (Institute of Medicine, 2015) and have assigned protection factors substantially higher than those of N95-type masks (Tompkins and Kerchberger, 2010). PAPRs are also better suited to periods of very high demand: whereas N95 masks are designed for one-time use, commercially available PAPRs are designed to be sterilized and reused multiple times (Rebmann and Wagner, 2009).

PAPRs are generally in short supply in most US healthcare institutions, which typically seek the lowest cost approach to respiratory protection for HCWs. The acquisition costs for commercial PAPRs are ∼100–1,000-fold higher than N95 masks: purchasing managers report that a low-cost PAPR retails for ∼800.00 USD and a medium-priced device sells for ∼2,000.00 USD (3M, 2021) whereas N95-type masks normally cost ∼1.50 USD per unit in bulk (the cost of N95-type masks increased 5–10 fold during the COVID-19 pandemic, however) (Ivry and Kochkodin, 2020). PAPR filters must be replaced regularly and are also relatively expensive (Institute of Medicine, 2015). Thus, despite multiple Federal panels and reports spanning a period of two decades calling for innovations in respiratory protection (Sinha et al., 2020), there has been little concrete response. This is a setting in which open-source product development (OSPD) (Huizingh, 2011) has the potential to make a substantial contribution.

**FIGURE 1:**
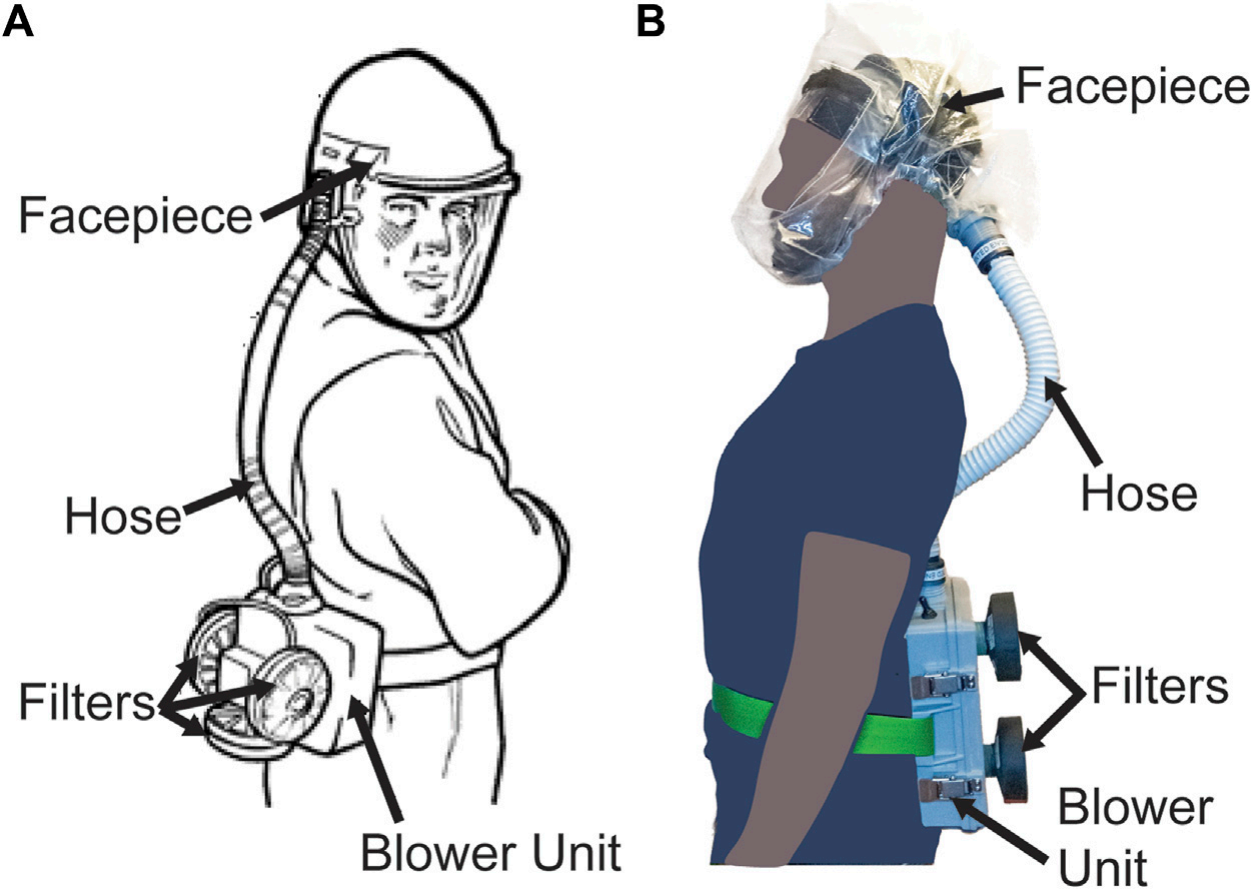
PAPR components. **(A)** Diagram of PAPR components, adapted from OSHA.gov (OSHA Technical Manual, 2006). **(B)** PanFab PAPR described in this work.

PAPRs are composed of three primary functional components: the filter cartridge, the blower unit, and the facepiece, which is connected to the blower via a flexible hose. Additional components, such as low flow rate alarms, enhance user safety and usability (Figure 1). The blower unit and its associated power and control systems are enclosed inside an air-tight housing. This housing couples to the filter cartridges and to the hose. The blower unit pulls room air through one or multiple high-efficiency particulate air/high-efficiency particulate absorbing (HEPA) filter cartridges, thereby removing aerosols and small particles. The blower then pushes the filtered air into the facepiece (also known as a hood) through the hose, and it is breathed in by the user. In the case of a loose-fitting facepiece, air also escapes through gaps between the facepiece material and the user’s body. The presence of positive pressure in the facepiece ensures that unfiltered outside air does not enter the facepiece and is not inhaled by the user.

The current shortage of PAPRs likely reflects the complexity of these devices, which have multiple components, each requiring significant expertise to design, engineer, and test. Resources describing the design criteria for PAPRs used in healthcare settings are scarce because most designs are proprietary, making it challenging for new or local manufacturers to help address shortages. Additionally, the regulatory approval process for PAPRs via NIOSH is significantly more complex than regulatory approval processes for simpler devices such as face shields (Mostaghimi et al., 2020) that have already been produced in bulk by non-traditional suppliers. In the US, PAPRs are regulated by the Occupational Safety and Health Administration (OSHA) under the Respiratory Protection standard (29 CFR 1910.134). This requires that PAPRs be approved by NIOSH but does not require 510(k) premarket notification or clearance by the Food and Drug Administration (FDA) (eCFR, 2021; CDC, 2014). One resulting challenge is that NIOSH testing standards are highly prescriptive, but the physical and engineering principles underlying these tests are not always obvious. The prescriptive approach may be appropriate under normal circumstances when it is important to maintain quality standards in the face of cost pressure, but it is problematic in emergency conditions in which approved testing apparatus are in short supply. In the current work we therefore rely on “NIOSH-equivalent” testing to assess performance.

We sought to create public domain PAPR designs with non-restrictive licensing that would help to address current and future shortages in respiratory protection. We also sought to use open source product development to address the broader problems of supply chain disruption caused by healthcare emergencies and shortages of medical supplies in resource-limited environments (e.g., developing nations). After consulting with clinicians and infection control specialists, we focused our efforts on designing filter cartridges and blower units (consisting of a housing, blower, battery, flow control system, and flow control alarm), the two PAPR components most commonly in shortage. NIOSH standard testing procedures (STPs) (CDC, 2020b), which specify the testing requirements needed for NIOSH approval of PAPRs, provided performance specifications for the filter cartridge and blower unit components (Supplementary Material S1); we used these specifications to guide PAPR design.

In this paper, we describe the design, validation, and user testing of modular PAPR components- the filter cartridges and the blower units, developed by the Greater Boston Pandemic Fabrication Team (PanFab) (PanFab, 2021). These components are intended to provide alternatives to standard commercially available PAPR components and to be locally manufacturable in times of severe PAPR shortage. For both the filter cartridge and the blower unit components, we describe a “PanFab Custom Design” and a “PanFab Commercial Design” to accommodate different scenarios with respect to shortages of materials. The Custom design has less reliance on commercial products and supply chains and can be fabricated in large part using additive manufacturing (3D-printing) methods for low volume production or injection molding for high volume needs (Antonini et al., 2021). The Commercial design relies on commercially available parts made for other products and requires fewer custom fabrication steps, facilitating rapid introduction of new, locally fabricated units. The PanFab PAPR components are modular and interchangeable: any combination of components can be used together and also with traditional PAPR components from leading suppliers. For example, the PanFab Custom Filter can be used with the PanFab Commercial Blower Unit and vice-versa. The PanFab PAPR components are also compatible with the widely used ILC Dover Sentinel XL PAPR facepiece (ILC Dover, 2021) and filters. The blower unit can also be adapted to other commercially available PAPR facepieces by fabricating a slightly modified hose-to-facepiece connector. Under the provisions of a Creative Commons Attribution-ShareAlike 4.0 International Public License, other entities are free to use components of the PanFab PAPRs by themselves, in their own designs, or to innovate these designs further.

Initial prototype testing was conducted at academic laboratories using equipment and supplies that were available during the COVID-19 pandemic. User feedback on the functionality and comfort of the designs was then obtained at a major US academic medical center from four participants: two healthcare providers and two research technicians who used PAPRs regularly as part of standard PPE prior to the pandemic. User feedback was elicited to identify possible points of improvement for future PAPR designs and is intended to be part of an iterative process; insufficient testing was performed to achieve statistical significance or meet NIOSH certification standards. We intend for this to happen as part of scale-up prior to large scale manufacturing. Performance testing was conducted using alternative apparatus and methods than those prescribed by NIOSH, which are hard to replicate outside of a conventional certification laboratory. An additional limitation of our approach is that PAPR certification, like certification of most medical products, requires a manufacturing process controlled by a quality management system (e.g., one similar to ISO 9001 standards). Achieving this standard is only possible in a commercial setting, and we are therefore collaborating with an industrial partner to create a design amenable to NIOSH standards. Future users of the PanFab PAPRs must perform their own testing and confirm that fabricated products meet the requirements of FDA Emergency Use Authorizations and similar regulatory guidance. We return to this issue in the discussion section.

## Methods

### Prototype Development

NIOSH requirements (NIOSH STP CVB-APR-STP-0081) specify that PAPRs have a minimum filtration efficiency of 99.97% for NaCl aerosols (this corresponds to the 100-N class of PAPRs). To reduce the power required to drive air through filters, they should also have as little pressure drop as possible at the minimum required flow rate of 170 L per minute (lpm; NIOSH STP RCT-APR-STP-0012). For the PanFab Commercial PAPR, we selected a commercially available HEPA filter that is used in consumer vacuum cleaners and widely available; we speculated that supply of these filters is unlikely to be significantly affected by disruption of medical device supply chains caused by COVID-19 or similar pandemics. For the PanFab Custom PAPR, a custom-designed filter cartridge was designed to be lighter in weight and have a lower form factor as compared to the Commercial version.

Design of the blower units focused on meeting the required flow rate of ≥170 lpm and overcoming pressure drops caused by the filters and tubing in the air flow path at this flow rate. In addition, blower units needed to be operational for at least 1 h, comfortable to wear, sterilizable, airtight, and relatively silent (NIOSH STP RCT-APR-STP-0030). For a full list of design requirements and NIOSH testing requirements, refer to Table 1 and Table 2, respectively. Supplementary Material S2 provides a full discussion of the design methodology and resulting prototype components.

**TABLE 1:** PanFab PAPR design components, selection criteria, specifications, and commercial components.

Component	Design/Selection criteria	PanFab component specifications	Traditional commercial component
Filter	• High filtration efficiency under NIOSH filtration test conditions	• Milwaukee HEPA rated filter, part number: 49-90-1900	• ILC Dover high efficiency particulate air filter, part number: S-4002
	• Minimal pressure drop at required flow rate	• Custom Filters LLC 100-P rated filter	
	• Easy replaceability		
Blower Unit	• Flow rate of over 170 lpm	• Delta electronics centrifugal blower, part number: 603-2093-ND	• ILC Dover sentinel XL PAPR blower unit, part number: S-2002
	• Static pressure rating sufficient to overcome pressure drops and provide required flow rate	○ Maximum flow rate: 518 lpm	
	• Power rating low enough to minimize battery size/weight	○ Maximum static pressure: 403.5 Pa	
		○ Rated voltage: 12VDC	
		○ Current rating: 0.58A	
		○ Noise: 50.5 dBA at 1m	
Housing	• Non-porous, hard material	• Custom housing:	
	• Airtight sealing	○ ABS 3D-printed or injection molded	
	• Easy opening/closing for battery charging	○ EPDM 1/4″ thick cam and groove gasket for sealing at filter outlet and silicone 1/8″ nominal diameter O-ring for housing lid sealing	
	• Easy coupling/decoupling with filters	○ Draw latches for housing lid closure	
	• Low weight and form factor	• Pelican case housing:	
	• Easily and cheaply 3D-printable and injection moldable	○ Pelican V100 vault small pistol case	
Control system	• Regulate flow rate	• Arduino R3 controller	
	• Measure flow rate	• OSH Park custom-printed shield	
	• Sound an alarm at least 80 dBA at ears if flow rate falls below 170 lpm	• Sensirion differential pressure sensor, part number: SDP810-500PA	
		○ Range: −500 to 500 Pa	
		• Precision Electronics Corporation potentiometer, part number: RV4NAYSD103A	
		○ Response: linear	
		○ Resistance: 10k-ohms	
		○ Power Rating: 2W	
		• Mallory Sonalert Products piezoelectric buzzer, part number PS-580Q	
		○ Voltage rating: 5V–15V	
		○ Current: 150mA	
		○ Frequency: 2.8 kHz	
		○ Sound Level: 100 dB at 12 V and 100 cm	
Battery	• Match blower power characteristics	• Tenergy NiMH battery pack, Amazon Standard Identification Number: B077Y9HNTF	• ILC Dover sentinel XL PAPR battery, part number: S-2003
	• Capacity to run the PAPR for at least 1 h	○ Voltage: 12V	
	• Lightweight and small form factor	○ Capacity: 2,000 mAh	
	• Safe for use in medical setting	○ Maximum discharge current: 2A	
Facepiece	• Coverage of nose and mouth	• University of Washington VHA ADAPT PAPR Hood	• ILC Dover sentinel XL PAPR clear hood, part number: S-3101
	• Conducive to communication		
	• Compatible with equipment such as stethoscope		
	• Compatible with eyewear		
	• Avoids fogging		

**TABLE 2:** PanFab PAPR component validation test type, regulatory guidance, and alternative test results. Full STPs available as Supplementary Material 1.

Test type	Relevant NIOSH STP	Result of NIOSH-alternative test
Filtration efficiency	Procedure No. CVB-APR-STP-0081 determination of particulate filter efficiency level against solid particulates (PAPR 100-N)	Milwaukee filters: 99.99%, 100% at 300 nm and 230 lpm
		Custom filter: 99.99% at 300 nm and 230 lpm
	Procedure No. TEB-APR-STP-0001 determination of particulate filter penetration (PAPR) test	Milwaukee filters: 99.18% and 99.58% at 170 lpm
		Custom Filter: 99.98% at 170 lpm
Flow rate	Procedure No. RCT-APR-STP-0012 determination of air flow for powered air-purifying respirators	240 lpm at 70% blower duty cycle
Qualitative fit	Procedure No. RCT-APR-STP-0067	Pass, n = 1
	Particulate respirator qualitative fit test utilizing saccharin or bitrex solutions	
Sealing	Procedure No. CVB-APR-STP-0010 determination of respirator fit, quantitatively using corn oil aerosol, for powered air-purifying respirators with loose-fitting respiratory inlet coverings	Pass, n = 1
Noise level	Procedure No. RCT-APR-STP-0030: determination of noise level test, power air-purifying espirator with hoods or helmets	58.1 to 59.2 dBA at full battery charge and maximum blower speed
Low flow rate alarm	Procedure No. CVB-APR-STP-0085 determination of low flow warning device sound level	Between 82.95 and 84.7 dBA at 230 lpm flow rate
	Procedure No. CVB-APR-STP-0088 determination of low flow warning device activation	
Audibility test	Procedure No. CVB-APR-STP-0089 determination of communication performance test for speech conveyance and intelligibility	Pass, n = 1

### Prototype Testing

NIOSH has developed several STPs for testing the safety and functionality of PAPR components (CDC, 2020c). Third-party commercial laboratories typically test PAPRs to these STPs as means to establish compliance with NIOSH standards. However, high demand for testing during the COVID-19 pandemic made many of these test options either unavailable or considerably delayed. As an alternative, we devised apparatus intended to perform the physical, chemical, and engineering measurements described in NIOSH STPs but using materials readily available in university laboratories. Prototype testing was carried out across several university laboratories at MIT on the filters, blowers, power systems, control and warning systems, and the seals between components. The use of these “NIOSH-equivalent” tests allowed us to perform PAPR design and testing in an emergency setting, but does not obviate the need for testing to NIOSH STPs prior to large scale manufacturing. The type of testing needed for clinical implementation of our designs in an emergency setting remains to be determined; as the COVID-19 pandemic recedes we hope that regulatory agencies will provide better guidance on balancing risks under these circumstances.

A loose-fitting facepiece known as the VHA ADAPT PAPR Hood, developed by the Center for Limb Loss and MoBility (CLiMB) at the University of Washington (UW Mechanical Engineering Research Centers, 2012), was used for prototype testing. This facepiece was used in place of standard commercial PAPR facepieces such as the ILC Dover Sentinel XL PAPR facepiece both because these were in limited supply and because we sought to create an all open-source design (testing of the CliMB facepiece is beyond the scope of the current work and expected to take place independently). However, the PanFab PAPR is compatible with ILC Dover facepieces.

#### Filter Testing

**FIGURE 2:**
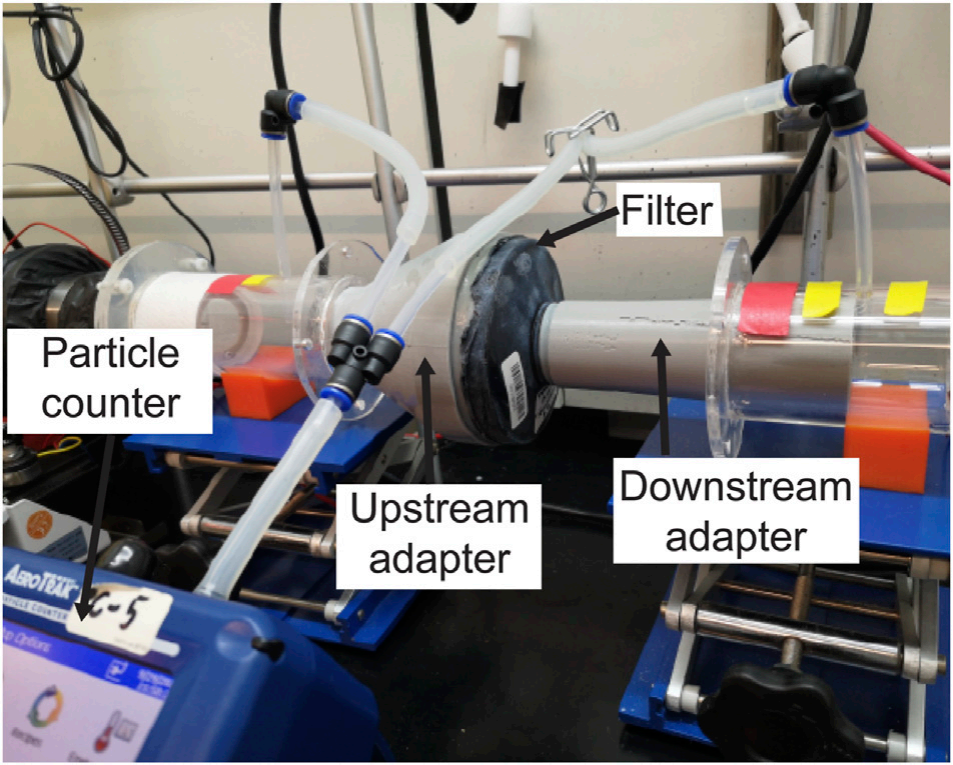
Loading filtration test setup, with filter cartridge in line with KCl-containing air stream. Other components of the apparatus have been previously described (Plana et al., 2020).

NIOSH performs two distinct filtration efficiency tests: a full loading test and an instantaneous (abbreviated) test, the latter of which estimates the lowest filtration efficiency expected at the start of a filter’s service life. NIOSH performs the loading filtration efficiency test of 100-N class of PAPR filters using 75 nm median diameter NaCl aerosols (NIOSH STP CVB-APR-STP-0081). To test the filtration efficiency for the PanFab PAPR filters, we modified a previously described university-based apparatus (Plana et al., 2021) originally used to assess the filtration efficiency of N95-type FFRs (Figure 2). Due to the unavailability of NaCl aerosol generators, KCl was used instead. A Collison Nebulizer (MRE 6-Jet, BGI Inc., Waltham, MA) generated aqueous KCl particle streams, a Handheld Particle Counter (TSI 9306-V2 AeroTrak, TEquipment, Long Branch, NJ) was used to count particles, and a differential pressure gauge (purchased from McMaster-Carr, Elmhurst, IL, part number 4125K21) measured the pressure drop across the filter.

The AeroTrak Counter had a lower measurable limit of 300 nm particle size, which we expect to result in a more conservative estimate of filtration efficiency than particle sizes specified in NIOSH STPs (Stafford and Ettinger, 1967). Filter cartridges were placed within the apparatus in line with the flow of the KCl-containing particle stream. Special 3D-printed adapters, sealed to the cartridges, were tightly coupled to upstream and downstream air ducts, ensuring no leakage. Filtration efficiency was computed from the measured KCl concentrations upstream and downstream of the cartridges.

The US Code of Federal Regulations (42 CFR § 84.175) specifies that PAPR performance should also be tested using dioctyl phthalate to assess its resistance to oil droplets (NIOSH STP TEB-APR-STP-0001). This is an instantaneous filtration test in which a filter cartridge is challenged with a dioctyl phthalate-containing aerosol for approximately 10 s. In lieu of dioctyl phthalate, which is a suspected carcinogen (National Biomonitoring Program, 2019), an aerosol containing the oil polyalphaolefin (PAO) was used. PAO is a chemical representative of the most widely used class of synthetic lubricants and a substitute for dioctyl phthalate accepted by the US military (Carlon et al., 1991). Filtration efficiency testing was then performed as described above.

#### Air-Flow Testing

**FIGURE 3:**
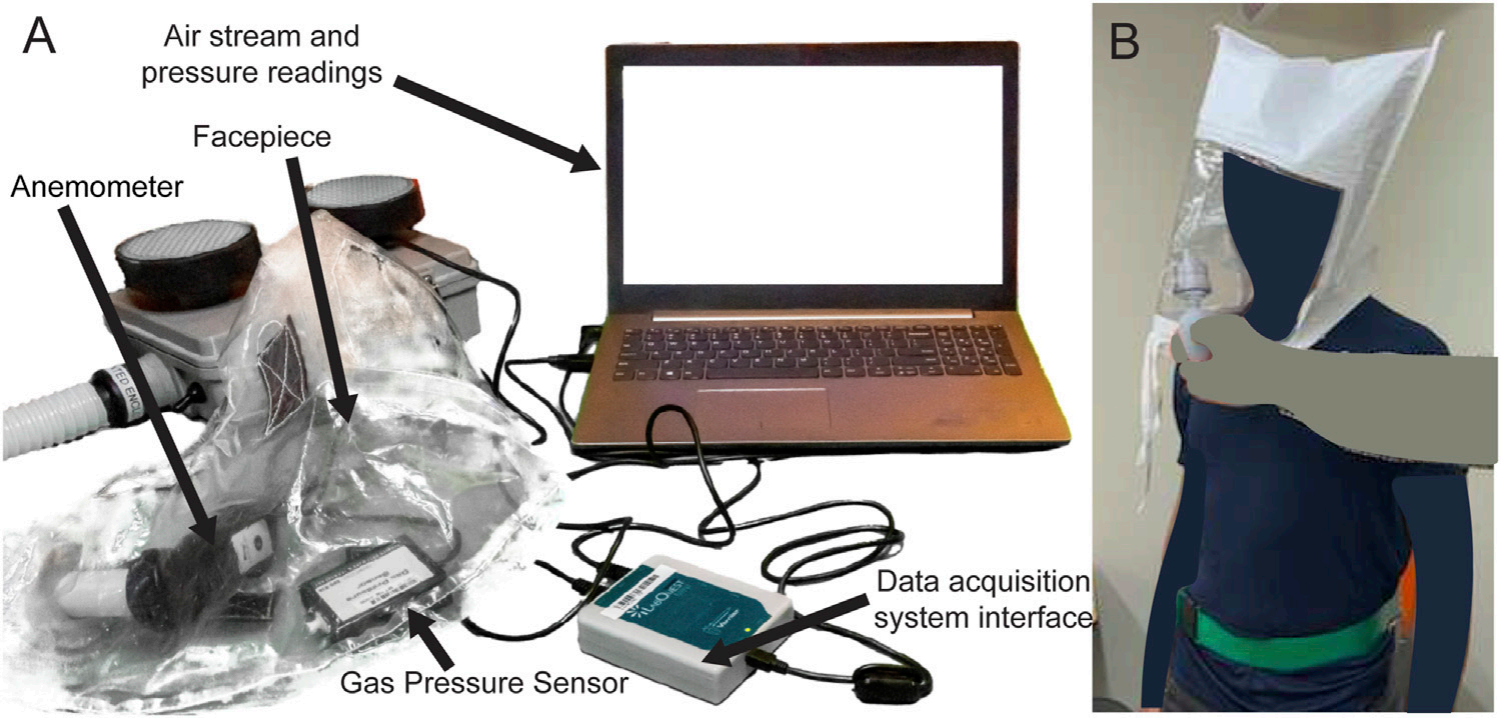
**(A)** Test setup to measure flow rate and the positive pressure inside the facepiece, using Vernier Anemometer and Gas Pressure Sensor. PAPR facepiece contains anemometer and gas pressure sensor. Tape covers the neck opening of the facepiece for testing. **(B)** Bitrex Fit Test setup.

NIOSH requires a minimum flow rate of 170 lpm for PAPRs that have loose fitting facepieces (Approval Tests and Standards for Air-Purifying Particulate Respirators, 2020), as described in NIOSH STP RCT-APR-STP-0012. To measure flow rate, the STP describes connecting a vacuum chamber, evacuated with a vacuum pump, to a running PAPR blower unit and using a dry test meter to measure flow rate. In the absence of this setup, a conventional impeller type anemometer (Vernier Software and Technology, Beaverton, OR) was connected to the air inlet at the facepiece, with the neck opening sealed with tape (Figure 3A). An adapter was 3D-printed to couple the facepiece inlet to the anemometer flow area, such that all the flow into the facepiece passed through the cross-sectional area of the anemometer inlet. Flow rate was calculated by multiplying the air velocity recorded on the anemometer with the cross-sectional area. Additionally, a Vernier Gas Pressure Sensor placed inside the facepiece measured the positive pressure created in the facepiece. While not as precise as the procedure described in NIOSH STP RCT-APR-STP-0012, we expect that the modified test provides a close approximation of the flow rate using equipment available in a standard laboratory.

#### Facepiece Fit Testing

To determine if unfiltered air can enter the facepiece, we conducted a Bitrex (Edinburgh, Scotland) qualitative fit test as described in NIOSH STP RCT-APR-STP-0067 (Current Standard Testing Procedures for Air-Purifying Respirators, 2020) on one test subject (Figure 3B). This test evaluates whether flow rate into the facepiece is sufficient to prevent unfiltered ambient air from reaching the user; the unfiltered ambient air contains an aqueous aerosol of denatonium benzoate (a bitter chemical) and subjects are asked if they can taste it during the test. Under the NIOSH Interim Final Rule for PAPR testing, the fit test is to be performed as per NIOSH STP CVB-APR-STP-0010, wherein subjects donning the PAPR are sent into a room filled with corn oil aerosol. If the subject is able to taste the corn oil, the test is deemed to have failed. However, given the lack of a dedicated room in which to perform this test we adopted a Bitrex fit testing procedure which is commonly used in hospital settings to evaluate respirators of different types. The Bitrex test is a permissible substitute for corn oil testing according to pre-pandemic NIOSH testing requirements.

#### Housing Sealing Testing

A modified Bitrex fit test was also used to evaluate the quality of seal created by the PAPR housing (we were unable to use the standard CVB-APR-STP-0010 testing protocol due to limitations in the availability of the necessary equipment). Bitrex was sprayed on the sealing surfaces of the blower units, including where the two parts of the blower unit join, the switch mount, and the filter connections. If a test subject can taste the Bitrex solution, the test was judged to have failed. Commercial Milwaukee vacuum cleaner filters sourced from a local home improvement store (or on-line) were connected to both blower units during these tests.

#### Auditory, Communication, and Low Flow Rate Alarm Testing

NIOSH STP RCT-APR-STP-0030 requires the noise level at each ear, with the blower unit running at maximum flow, not exceed 80 A-weighted decibels (dBA). We used a Vernier SLM-BTA Sound Level Meter to measure sound level. We also tested ease of communication following NIOSH STP CVB-APR-STP-0089. The subject was tasked with speaking and listening to a set of words. Ease of communication was evaluated by counting the number of words correctly transcribed in each task, normalized by baseline performance without the PAPR.

NIOSH STP CVB-APR-STP-0085 requires that PAPRs have an alarm to alert users when air flow rate falls below the minimum level of 170 lpm. Auditory alarms are required to be louder than 80 dBA. We used a Vernier SLM-BTA Sound Level Meter to measure the sound level of the low flow rate alarm after triggering the alarm by manually restricting the air flow at the facepiece inlet.

## Results

**FIGURE 4:**
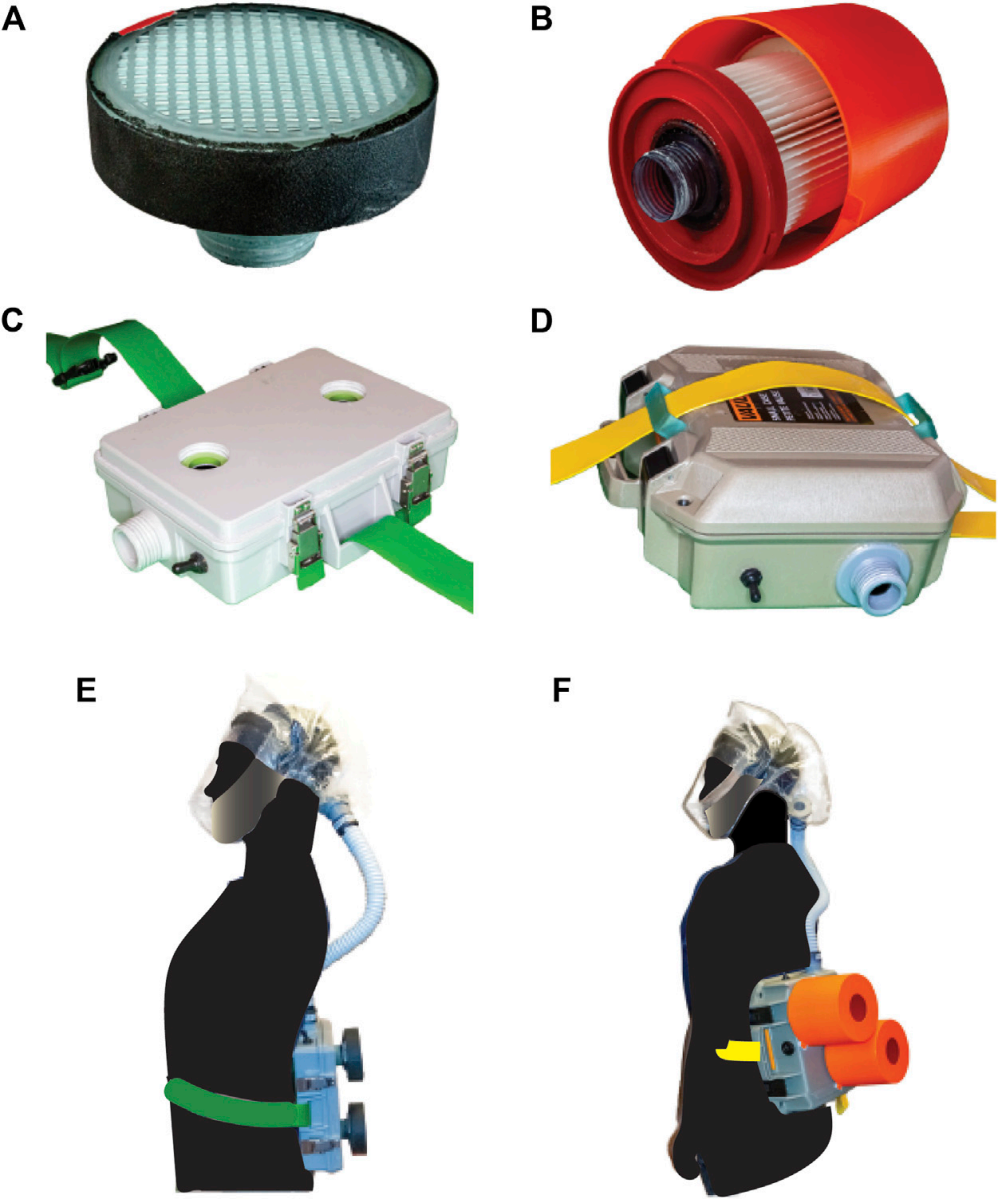
PanFab PAPR components. **(A)** PanFab Custom filter cartridge. **(B)** PanFab Commercial filter cartridge. **(C)** PanFab Custom blower unit. **(D)** PanFab Commercial blower unit. **(E)** PanFab Custom Design (Custom filter cartridge plus Custom blower unit). **(F)** PanFab Commercial Design (Commercial filter cartridge plus Commercial blower unit).

PanFab Custom and Commercial Designs were developed for both filter cartridges and blower units (Figure 4). For a full description of the filter and blower unit designs, refer to Table 1 and Supplementary Material S2. A Milwaukee Tool (Brookfield, WI) HEPA-rated vacuum cleaner filter (part number 49-90-1900) was selected as the PanFab Commercial Filter Cartridge. This filter model is readily available in North American home improvement stores and similar types of filters are available in other countries, driven by increasing concern about the health effects of exposure to silica dust in construction (Poinen-Rughooputh et al., 2016). Widespread innovation is occurring in this area (with many new tools having integrated HEPA filtration) and groups interested in other ways to develop respiratory protection devices for HCWs are encouraged to stay abreast of these developments. A custom 3D-printed adapter converts the outlet of the Milwaukee filter to standard NATO 40-millimeter threaded connection, allowing it to be used with the PanFab and other commercial blower units. With slight modification of the adapters, other commercial HEPA-rated vacuum cleaner filters could be used as alternatives. A custom 3D-printed filter cover protects the filter fabric. The Custom variant of the PanFab filter cartridge was designed in collaboration with Custom Filters LLC (Aurora, IL) to have the necessary 100-P rating while remaining small and light. Two filters were used in both variants of the PAPR as opposed to one, so as to minimize the pressure drop for a given flow rate and to provide redundancy.

A centrifugal blower (Delta Electronics, Neihu, Taiwan, Part Number BFB1012HD-04D4L) generates a 230 lpm flow rate, higher than the required 170 lpm, and a 12-volt (V) NiMH battery pack (Tenergy, Fremont, CA, Amazon Standard Identification Number: B077Y9HNTF) was used to power it; this battery pack was sufficient for ∼4 h of continuous use. The battery can be charged in various ways, including with solar power, as long as 12 Voltage Direct Current (VDC) < 1.8 A (Amp) can be supplied through an electrical connector compatible with that used in the battery. A wide variety of 12V NiMH and lithium ion battery packs are available at home improvement centers and could be used as substitutes following performance testing.

Control circuitry was based on a standard Arduino R3 board (Arduino LLC, Boston, MA) with a custom-fabricated shield (OSH Park, Portland, OR). Discrete components connected to the shield included a 10 kiloohm potentiometer (Precision Electronics Corporation, North York, ON, Canada, part number RV4NAYSD103A), differential pressure sensor (Sensirion AG, Staefa, Switzerland, part number SDP810-500PA), and piezoelectric buzzer (Mallory Sonalert Products Inc., Indianapolis, IN, part number PS-580Q). These components were used for control and alarm tasks such as regulating the air flow rate, measuring flow rate, and sounding the low-flow buzzer. All of the discrete components are readily substitutable with similar products made by multiple manufacturers. We established that the Sonalert buzzer generated a sound of at least 80 dBA (as per CVB-APR-STP-0085) when the flow rate fell below a NIOSH specified threshold (as per CVB-APR-STP-0088). The shield circuit diagram, fabrication files, and Arduino code are all available in Supplementary Material S4.

The housings that enclose the blower, battery, and control components were designed to be airtight when closed, with the filters and hose attached. In the case of the Commercial Design, a Pelican V100 Vault Case (Pelican Products, Torrance, CA) was used with custom made “inserts” for connection to filters and hose via NATO 40mm connections. The Custom housing was designed to be smaller and lighter in weight, with integrated connections to the filters and hose. A hood coupler and a locking ring were designed to connect the hose to the facepieces, which use a NATO 40 mm threaded connection. Finally, a hose adapter was designed to form air-tight connections between the ends of the plastic hose, the housing outlet, and the facepiece inlet. While all custom-made parts, including the custom housing, were 3D printed for prototyping, these designs were also optimized for injection molding to facilitate future high volume production (Antonini et al., 2021). The CAD files for all custom and 3D printable/moldable parts, as well as printing instructions, are in Supplementary Material S4.

With their respective filters installed, the PanFab Custom and Commercial PAPRs weighed 1.87 and 3.36 kg, respectively. Both PanFab PAPRs are worn on the waist using a Skil-Care (Yonkers, NY) PathoShield Gait Belt. This 50 mm wide web belt is heat-sealed (rather than stitched), has a liquid-proof plastic coating covering the vinyl webbing (for easy cleaning) and a Delrin side-release buckle; it is widely available in healthcare settings and many functionally identical substitutes exist. The maximum enveloping cuboidal dimensions of the PanFab Custom and Commercial PAPRs (including their respective filters) along lateral, longitudinal, and sagittal axes are 21 cm × 25.8 cm × 12.4 cm and 30.6 cm × 33.6 cm × 25.4 cm respectively. Run time for the PanFab blower units was measured to be approximately 3 h and 55 min and charge time approximately 2 h and 53 min at 0.9 A charging current, with a variance on the order of 1–2 min for the runtime and charge time respectively. This compares favorably to the Ford-3M Limited-Use Public Health Emergency PAPR blower unit, which weighs 2.7 kg, runs for 4–6 h, and has a charge time of 1.5 h with a 3 Amp hour battery (Limited-Use Public Health Emergency PAPR, 2021). Alternative battery packs could easily be added to the PanFab design to increase run time; charge time is primarily a function of the charger.

The estimated cost in parts for a single unit of the PanFab Custom PAPR is 284 USD and for the PanFab Commercial PAPR is 328 USD. A detailed *Bill of Materials* for both PAPRs is provided in Supplementary Material S4. Consultation with industry experts in the area of respiratory protection, including a NIOSH certified manufacturer, allowed us to estimate the final cost for PanFab designs including commercialization and labor costs, and account for discounts for materials ordered in bulk. This yielded an estimated per-unit cost for the PanFab Custom PAPR of 310 USD. In comparison, the Ford-3M PAPR designed for pandemic response has been reported to sell for 715 USD (Cole, 2020). This compares with low-cost commercial PAPR prices of ∼800 USD per unit, a 3 M Versaflo PAPR price of ∼1,775 USD per unit (3M, 2021), and N95 mask costs of ∼1.50 USD per unit.

The development of functional PanFab PAPR prototypes took a total of eight months with initial product specification and prototyping completed in three months. Additional manufacturing modifications took an additional two months. The latter set of modifications readied the PAPRs for large-scale production via injection molding. While prototyping was underway, the design validation and testing procedure was established over a period of five months. Design validation was constrained by the availability of testing resources under pandemic conditions. This, together with a dearth of explanations for testing specifications, were the primary factors slowing completion of the project. We hope that our descriptions of testing approaches and regulatory documents will allow others to proceed more quickly.

### Testing and Validation

PanFab PAPR components underwent a series of rigorous testing and validation steps. As mentioned earlier, many traditional NIOSH tests were not readily available from commercial laboratories due to high demand associated with the COVID-19 pandemic. Moreover, tests are highly prescriptive and not easily set up in an academic research laboratory. A further compromise we were forced to make is that full NIOSH certification was simply not possible for the PanFab PAPRs, regardless of testing procedures, in the absence of documentation that they would be manufactured according to established quality-control criteria. Compliance with these manufacturing standards is important, but it is secondary to our goal of developing a functional PAPR design. We therefore established alternate test setups and protocols to replicate several NIOSH tests (Table 2). The ability of the final designs to pass these tests should increase the confidence of traditional and non-traditional manufacturers that PanFab designs are very likely to pass full NIOSH certification; we very strongly encourage formal certification testing prior to use of these designs in a healthcare setting.

#### Filter Tests

Two filter cartridges were used in PanFab PAPRs. One was an off-the-shelf HEPA-rated vacuum cleaner filter manufactured by Milwaukee, and the other was a 100-P rated filter designed in collaboration with Custom Filters LLC. As part of loading test, two Milwaukee filter cartridges and one Custom Filters filter were challenged with KCl aerosol at an operationally equivalent flow rate of 230 lpm. Filtration efficiency with 300 nm aerosol size was found to be 99.99 and 100.00% for two replicate Milwaukee filters and 99.99% for the Custom Filters filter, thereby exceeding the NIOSH salt aerosol filtration efficiency criteria of 99.97%. Equipment was not available to measure filtration efficiency below 300 nm but it is generally observed that HEPA filtration efficiency is lowest at 300 nm and increases as particle size falls (US EPA, 2019). Results from our testing apparatus also correlate with prior testing done at ICS Laboratories, Inc. (Brunswick, OH) for N95-type respirators; ICS Laboratories, Inc. performs third party testing to NIOSH standards using NIOSH STPs (for more information, visit Cleanmask.org (Clean Mask, 2021)).

In the PAO-based instantaneous filtration test carried out by Custom Filters LLC, two Milwaukee filter cartridges and one Custom Filters cartridge were challenged with 90.56 mg/m^3^ PAO aerosols at 85 lpm. Filtration efficiency was 99.18 and 99.58% for the two Milwaukee filter replicates and 99.98% for the Custom Filters filter. While the Milwaukee filter does not pass the NIOSH requirement of efficiency higher than 99.97% for oil-based aerosol, consultation with experts on NIOSH certification and regulation led us to conclude that this would not necessarily preclude use in a healthcare setting, given the low concentration of oil aerosols found in this environment. Oil aerosols are primarily a concern in industrial settings in which PAPRs are also used.

#### Air Flow Tests

Using the apparatus described in the Methods section, flow rate was calculated as the product of the measured velocity and the cross-sectional area of the anemometer. Flow rate with a 70% blower Pulse Width Modulation (PWM) duty cycle was measured to be close to 240 lpm for both filter types. A positive pressure of 40 Pa was recorded inside the facepiece.

#### Facepiece Fit Tests

Qualitative fit testing, using Bitrex as the testing agent was performed repeatedly on one subject, as per RCT-APR-STP-0067. Tests were performed with all configurations of the Commercial and Custom PAPR designs (i.e., using Custom and Commercial blower units with Commercial and Custom filter cartridges). The University of Washington (CLiMB) (UW Mechanical Engineering Research Centers, 2012) facepiece was used in our tests. No Bitrex could be tasted by the subject in any of the test configurations, in any of the tasks prescribed in the facepiece fit test STP, indicating a successful result.

#### Housing Sealing Tests

Bitrex was sprayed on the sealing surfaces of the PAPR blower units. No Bitrex was tasted when tests were performed with both of the PAPR housings, indicating successful seals for both PanFab PAPR designs.

#### Auditory Communication Tests

Noise level in the facepiece at the ears was measured at between 58.1 and 59.2 dBA at full battery charge and maximum blower speed, which is lower than the 80 dBA limit set in RCT-APR-STP-0030. Low flow alarm sound level at the ears was found to be between 82.95 and 84.7 dBA at battery charge corresponding to the as-designed low flow condition of 230 lpm flow rate, which passes the 80dBA requirement in CVB-APR-STP-0085. The ability of PAPR wearers to communicate with other individuals was tested with one subject as per CVB-APR-STP-0089 with a 99.9% performance rating for listening and 74% for speaking tasks; both pass the required 70% threshold (see the STP for definition of performance rating). Full information regarding PanFab PAPR validation testing performed during pandemic conditions is summarized in Table 2.

### End-User Feedback

To evaluate factors affecting usability in a clinical setting, we created a clinical feedback questionnaire and distributed it to four participants who used and rated the performance of the PanFab PAPRs. Two participants were clinicians, who had not used PAPRs regularly prior to the pandemic, and two were research technical staff for whom PAPR use is a standard part of occupational PPE (Table 3).

**TABLE 3:** Test subject demographic information.

Subject	Height (cm)	Weight (kg)	BMI	Sex	Regular PAPR Use
1	160	57	22.3	Female	No
2	175	70	22.9	Male	No
3	178	100	31.6	Male	Yes
4	175	136	42.9	Female	Yes

User testing of PanFab PAPRs focused on three main criteria: 1) comparison to current PPE options; 2) sense of security with use in a clinical setting; and 3) comfort as compared to standard commercially available PAPRs. Additional questions assessed the PAPR facepiece alone, as well as ease of donning and doffing. A full list of questions and results are available in Supplementary Material S3. Four versions of the PanFab PAPR were assessed using different types of filters and blower units: one with a commercial filter and blower unit (PanFab Commercial Design), one with a custom filter and blower unit (PanFab Custom Design), and two versions with mixed Custom and Commercial filters and housings. Of all PanFab PAPR versions, the PanFab Custom Design performed most favorably: all four respondents rated the PanFab Custom PAPR superior to current PPE options, with a score of 4–5 on a 5 Likert-scale across the three criteria listed below (Table 4).

**TABLE 4:** Clinical feedback survey results. Score averaged among four users.

Enclosure	Filter	Compare to available PPE, average user score^a^ (n = 4)	Sense of security with use, average user score^b^ (n = 4)	Comfort compared to standard PAPR, average user score^c^ (n = 4)
Custom	Custom	4	4.75	5
Custom	Milwaukee	3.75	4.75	4.25
Pelican	Custom	3.25	4.5	3.75
Pelican	Milwaukee	3	4	3.25

a Score of 1 = PanFab PAPR much worse than current PPE options, 5 = PanFab PAPR much better than current PPE options.

b Score of 1 = Very uncomfortable, 5 = Very comfortable.

c Score of 1 = PanFab PAPR much worse than standard, 5 = PanFab PAPR much better than standard.

The three other versions of the designs (with a Commercial blower unit, filter, or both) performed favorably, with survey responses consisting of scores ranging from 3–5. Participants experienced more issues with mobility as compared to the fully-custom PAPR, and comments on PAPR versions using commercial parts emphasized the need for better weight distribution to improve balance. Participant comments across all PAPR design versions focused on possible improvements regarding the sizing and comfort of the PAPR facepiece, which was not part of the current study and was instead provided by collaborators. In sum, the clinical feedback suggested that the PanFab PAPRs are favorable alternative forms of PPE in terms of user comfort, mobility, and sense of security with use. Additional testing with a larger number of participants will be required to formally compare the performance of different PAPR configurations.

## Discussion

The successful design, prototyping, and testing of a PAPR by a volunteer team comprising medical professionals, scientists, student engineers, and concerned citizens (the PanFab team) demonstrates the potential for addressing pandemic-related shortages of relatively complex types of PPE using a rapid and iterative approach to prototyping and design (Antonini et al., 2021). The process generated near-final PAPR designs with full-time effort by three graduate engineering students, support from a clinical specification and testing team, access to standard academic laboratories, and modest financial support provided in part by the MIT COVID-19 Emergency Fund and the decision of the US National Institutes of Health to allow individuals paid by Federal research grants to devote time and effort to pandemic mitigation. Testing required more time than design and fabrication, as discussed below.

### Design and Results

The PanFab Commercial Design used commercially available components with custom-fabricated modifications while the PanFab Custom Design used additive manufacturing (3D-printing) to create a fully customized, lighter weight, and smaller enclosure. Frequent feedback from clinicians who use PAPRs in a hospital setting strongly influenced design decisions, particularly with respect to PAPR comfort and usability. Key design decisions included determining size and orientation of the housing, the number and orientation of the filters, and the method of donning/doffing the blower unit. Feedback from manufacturing experts yielded a design that is amenable to large-scale production by injection molding of the blower housing. PanFab PAPR designs are modular and compatible with several standard commercial PAPR components, including facepieces and filters, allowing for substitution of components in limited supply. The PanFab Custom Design compares favorably to the 3 M Ford Limited-Use Public Health Emergency PAPR (Limited-Use Public Health Emergency PAPR, 2021) with respect to weight and size, although the 3 M Ford unit appears to use higher performance batteries. Both PanFab designs also compare favorably to commercial PAPRs with respect to cost.

The safety and functionality of PanFab PAPRs was evaluated using protocols that closely followed NIOSH STPs and aimed to meet or exceed the functional objectives of those tests. Given the limitations imposed by pandemic conditions, it was necessary to use substitute tests in university laboratories rather than use a NIOSH-specific apparatus at a commercial pre-certification laboratory. PanFab PAPRs passed all of the performed tests, in various combinations of Commercial and Custom components. User feedback on the PAPRs was obtained from clinicians inexperienced in using PAPRs and technical research staff who routinely use PAPRs in a major Boston-area hospital. The PanFab Custom Design scored favorably as compared to the traditionally manufactured PAPRs (primarily from ILC Dover) available to hospital staff. This feedback also guided improvements that were made during iterative design of the prototype (e.g., using a different hood with the PAPR design to accommodate a greater diversity of user face shapes).

A limitation in the current study is that PanFab PAPRs were not tested in real-world settings, including for extended periods of time in a hospital. We are therefore unable to make claims about the durability of the designs in clinical use or the resistance of the units to damage. We were also unable to assess the efficacy of different methods for sterilizing PanFab PAPRs, but materials used in the designs are compatible with alcohol-based wipes (e.g., acrylonitrile butadiene styrene (Industrial Specialties Mfg and IS MED Specialties, 2019) allowing CDC recommendations for disinfecting PAPRs to be followed (CDC, 2020b). Previous research also suggests that ionized hydrogen peroxide techniques would be compatible with PAPR sterilization (Cramer et al., 2021). Additional usability, durability and sterilization testing await the availability of additional PAPR units assembled during production scale-up. However, the tests in this paper achieve the goal of PAPR design validation.

### Challenges in Design, Testing, and Regulatory Approval

During early specification and prototyping, we faced significant challenges in locating relevant design criteria for PAPRs. There also exists very limited information in the public domain on alternate testing equipment. Answers to questions such as the minimum time of continuous device operation required, relevant material characteristics for facepiece fabrics, and appropriate materials for the pathway for inhaled air were not readily available. We therefore sought out experts with relevant expertise. Substantial time and effort would have been saved had a centralized resource of information been available. To streamline the process for future crises, we have consolidated relevant information collected from US regulatory agencies in Supplementary Material S1. We encourage individuals from other countries to contact us with information relevant to products in their markets and will provide this updated information on PANFAB.ORG.

When evaluating our designs, we found that it was difficult to use NIOSH standard testing procedures since the necessary equipment was not readily available. Informed substitution was made difficult by prescriptive procedures and opaque objectives in terms of fundamental mechanical or physical principles being assessed. Our use of third-party commercial labs that test to NIOSH standards was also limited by long lead times and a requirement for multiple samples of each prototype, which would require financial resources beyond those available to our group. Thus, testing equipment and protocols, as opposed to design and fabrication, emerged as the primary challenge in developing the PanFab PAPRs.

Despite our best efforts, regulatory hurdles remain for use PanFab PAPRs in a clinical setting. To receive NIOSH certification, a product must be manufactured and submitted by a NIOSH-approved manufacturer with a quality management system in place. Under normal circumstances, this requirement guarantees the safety of products made in volume. However, this restricts the development of new products to NIOSH-approved manufacturers, which has had the effect of creating near-monopolies for some types of PPE. To improve resilience in future emergencies, regulators might consider how to optimally balance the risk of non-traditional PPE against the risk of no protection at all. We propose that consideration be given to rules that allow non-NIOSH certified fabricators to respond to declared healthcare emergencies while still complying with the most critical aspects of functional testing. Under this process, critical “go or no-go” tests would be defined by clearly described physical principles and corresponding testing protocols that could be performed on generally available laboratory equipment. The modified standards would include an explicit description of the end goal of the test and suggest a range of alternative devices that can be used to measure airflow, filtration efficiency, audibility, user testing etc. Rational substitution of instruments would also be allowed. While we do not advocate for relaxing standards under non-crisis conditions, modifying and streamlining testing procedures for prototype devices would reduce barriers to the entry of new products and promote innovation.

## Conclusion

The current COVID-19 crisis has revealed major weaknesses and points of failure in our health care system and its supply chains, particularly for PPE. This does not come as a surprise. Multiple studies over a 15-year period have decried the absence of innovation in the design and provision of respiratory protection for health care and other essential workers (Sinha et al., 2020). For example, a 2006 report by the US Institute of Medicine (IOM) called for urgent research to inform the design and development of new medical masks and respirators (Institute of Medicine, 2006); a 2008 IOM report addressed the design and engineering of more effective PPE (Institute of Medicine, 2008); the 2009 Project B.R.E.A.T.H.E. report laid out a comprehensive action plan for a new generation of respirators (Department of Veterans Affairs, 2009); and a 2019 consensus report from the US National Academies of Sciences echoed the same urgent needs (National Academies of Sciences, Engineering, and Medicine, 2019). Despite these repeated calls for action and greater innovation, there has been little response from the commercial sector or from government: in the COVID-19 pandemic, most innovation has come from volunteer groups of scientists and clinicians allied with maker communities with access to rapid prototyping and fabrication equipment, technology that is increasingly inexpensive and available to ordinary citizens (Sinha et al., 2020). Thus, open source product development (OSPD) (Huizingh, 2011) emerges as perhaps the only avenue to mitigating existing weaknesses while increasing product innovation under both normal and crisis conditions.

An OSPD approach is not a panacea and it is not free. As discussed above, it would be helpful for NIOSH and other regulatory agencies to develop less prescriptive testing procedures for products such as PAPRs. Funding is also essential. The work described here benefited from the generosity of many individuals but all attempts to fund it via competitive applications to foundations or universities were turned down because research into respiratory protection is not considered innovative by conventional academic criteria (salary support for grant-funded investigators was, however, temporarily available under relaxed grant guidelines from the US National Cancer Institute under NOT-CA-20-054). This speaks to a larger problem in matching acute healthcare needs to available expertise and necessary resources in academia, not just industry.

The production of a regulated medical product is difficult to achieve in the absence of commercial expertise. This is consistent with previous data showing that OSPD is most effective within the context of private-public partnerships (Bonvoisin et al., 2018). We are therefore working with an industry partner to develop PanFab PAPRs into commercial products. However, all the PanFab PAPR designs and software described here remain public domain resources and are available under non-restrictive Creative Commons Attribution-ShareAlike 4.0 International Public License; the design files, CAD files, and code are available on GitHub (https://github.com/labsyspharm/PanFab-PAPR-2021) and on the NIH 3D-Print exchange online repository (NIH 3D Print Exchange, 2020). Additionally, all materials needed for the construction and use of the design are also available in Supplementary Material S2, Supplementary Material S4. We hope that these materials serve as a resource for further development and innovation.

## Data Availability

The original contributions presented in the study are included in the article/Supplementary Material and can also be found on GitHub (https://github.com/labsyspharm/PanFab-PAPR-2021) and through the online NIH 3D Print Exchange (https://3dprint.nih.gov/discover/3dpx-015673; https://3dprint.nih.gov/discover/3dpx-015672; https://3dprint.nih.gov/discover/3dpx-015671; https://3dprint.nih.gov/discover/3DPX-015674).

## Supplementary Material

SUPPLEMENTARY MATERIAL S1 NIOSH PAPR standard testing procedures.

SUPPLEMENTARY MATERIAL S2 Full description of PanFab PAPR design methodology and selected prototype components.

SUPPLEMENTARY MATERIAL S3 Full clinical survey questions and results.

SUPPLEMENTARY MATERIAL S4 PanFab PAPR design materials and instructions, including: 3D-printing instructions, Arduino code, assembly instructions, bill of materials, CAD design files, custom Arduino shield design, and use instructions.
