# Supplementary material for: De Novo Powered Air-Purifying Respirator Design and Fabrication for Pandemic Response
Source: Front Bioeng Biotechnol. 2021 Sep 6;9:690905. doi: 10.3389/fbioe.2021.690905 (PMC8450396; doi:10.3389/fbioe.2021.690905)
Supplement: Supplementary file 1 [file DataSheet1.ZIP › Additional Materials/Supplementary Material 1.pdf]

DETERMINATION OF RESPIRATOR FIT, QUANTITATIVELY USING CORN OIL AEROSOL, FOR POWERED AIR-PURIFYING RESPIRATORS WITH LOOSE-FITTING RESPIRATORY INLET COVERINGS, STANDARD TESTING PROCEDURE (STP)

UNCONTROLLED COPY FOR INSPECTION AND USE

1. PURPOSE

This procedure establishes the method by which a generated corn oil aerosol is used for determining if powered, air-purifying respirators (PAPRs) supplied with loose fitting respiratory inlet coverings meet the facepiece-fit requirements at 42 CFR Part 84, Subpart K, Section 84.176(b).

2. GENERAL

This STP describes the Determination of Respirator Fit, Quantitatively Using Corn Oil Aerosol, For Powered Air-Purifying Respirators With Loose-Fitting Respiratory Inlet Coverings test procedure in sufficient detail that a person knowledgeable in the appropriate technical field can select equipment with the necessary resolution, conduct the test, and determine whether or not the evaluated product passes the test.

3. EQUIPMENT/MATERIAL

3.1. TSI Rear Light Scattering Laser Photometer, model 8587A, or equivalent with concentration range 1.0 µg/m^3 to >200 mg/m^3. See Figure 1.

3.2. NIOSH Dynamic Fit Software. (The software is used to monitor the particle count both inside and outside the respirator, calculate a fit factor for each exercise and the average fit factor for the trial in real time.)

3.3. Aerosol Generator, MSP Model 2045 High Output Aerosol Generator or equivalent - The aerosol generator is required to be capable of maintaining 5 to 100 mg/m^3 of corn oil challenge aerosol concentrations with a Mass Median Aerodynamic Diameter (MMAD) of 0.4 to 0.6 µm for the specified test duration in the test chamber. See Figure 2.

3.4. TSI model 8530, DustTrak II Aerosol Monitor, or equivalent - Range 0.001 to 400 mg/m^3 (Calibrated to ISO 12103-1, A1 test dust), and resolution ±0.1% of reading or ±0.001 mg/m^3, whichever is greater – See Figure 3.

3.5. Corn Oil - 99% Pure. CAS Number 8001-30-7 - Commercial product names are Maise/Maize Oil, Maydol and Mazola Oil.

3.6. Scanning Mobility Particle Sizer (SMPS), TSI model 3936 series - SMPS is composed of the TSI model 3080 Electronic Classifier, TSI model 3775 Condensation Particle Counter and Long Differential Mobility Analyzer (DMA). See Figure 4.

3.7. Environmental test chamber - The chamber shall be designed so that the individual(s) performing fit factor testing are visible at all times while in the chamber. The chamber design must include an entry vestibule designed to allow safe entry and exit from the chamber with minimal disturbance to both the aerosol concentration and the concentration uniformity. An example of a charged corn oil chamber is illustrated in Figure 5.

3.8. Chamber Communications - Electronic audio communications (chamber loudspeaker) are employed to transmit a real-time voice signal from laboratory technicians to test subjects to ensure that test subjects can clearly hear when to start and stop the test exercise regimen or receive safety information.

3.9. Facial Size Measurement Calipers - Calibrated face sizing calipers shall be used to measure the human test subject to the requirements identified in Appendix A. Examples of calipers are sliding measurement calipers: Seritex model GPM 104, 0-200 mm length, or spreading measurement calipers: Seritex model GPM 106, 0 – 300 mm width. These are shown in Figure 6 and Figure 7.

3.10. Facepiece Direct Probes. The sample probes shall be of the shape defined by Liu [AIHAJ (45); 278-283, 1984] and shall not interfere with the fit or function of the respirator. Figure 8 and Figure 9 are photographs of the probes used. Each probe bulkhead fitting is sealed using two rubber washers, one metal washer and one nut.

4. TESTING REQUIREMENTS AND CONDITIONS

4.1. Prior to beginning any testing, confirm that all measuring equipment employed has been calibrated in accordance with the testing laboratory's calibration procedure and schedule. All measuring equipment utilized for this testing must have been calibrated using a method traceable to recognized international standards when available.

4.2. General respiratory inlet covering fit requirements for PAPRs -

4.2.1. The fit test shall be performed using a panel of test subjects of various facial sizes measured in accordance with the NIOSH Bivariate Panel (NIOSH Panel). The measured face length and face width are used to designate the subject's NIOSH Panel cell number, as illustrated in Figure 10.

4.2.1.1. Face Width is the Bizygomatic Breadth measurement (Figure 9), using the spreading measurement calipers.

4.2.1.2. Face Length is the Menton-Sellion measurement (Figure 9), using the sliding measurement calipers.

4.2.2. Any PAPR part which must be removed to perform the respiratory inlet covering fit test shall be replaceable without special tools and without disturbing the facepiece on the wearer's face.

4.2.3. The respirator, including the respiratory inlet covering, shall be adjusted

UNCONTROLLED COPY FOR INSPECTION AND USE

according to the manufacturer's user instructions, prior to entering the chamber; however, upon entry into the test chamber neither the respirator, nor the respiratory inlet covering shall not be re-adjusted.

4.3. Test subject selection

4.3.1. For PAPRs with up to three sizes of loose-fitting respiratory inlet coverings, the test will be conducted employing 18 individual test subjects which represent the NIOSH Panel (Appendix 8.5). See Table 1 for the suggested test subject distribution in relation to the NIOSH Panel (NIOSH is allowing flexibility in the use of subjects from well-populated panel cells. NIOSH will attempt to test using a panel that contains at least one subject from each cell, but when subjects from all cells are unavailable they may be supplanted by adding subjects from more populated cells; however, no more than four subjects from any one cell may contribute to the overall test panel composition for any respirator under evaluation.)

Table 1: Recommended test subject distribution to be used for fit testing in relation to the NIOSH Panel

NIOSH Panel – Cell Number	Number of Test Subjects
1	1
2	1
3	2
4	4
5	1
6	1
7	4
8	2
9	1
10	1

4.3.1.1. For PAPRs supplied with respiratory inlet coverings in only one size - a subject failing to achieve a pass in their initial trial is considered to be a face-size failure. (no alternate size to try)

4.3.1.2. For PAPRs supplied with loose-fitting, respiratory inlet coverings in two sizes -

4.3.1.2.1. Subjects from panel cells 1-4 and cell 6 shall be tested first wearing the smaller size inlet covering. If the subject does not achieve a trial pass in the smaller size inlet covering, the subject can be tested again (second trial) wearing the larger size inlet covering.

4.3.1.2.2. Subjects from panel cells 7-10 and cell 5 shall be tested first wearing the larger size inlet covering. If the subject does not achieve a trial pass in the larger size inlet covering, the

UNCONTROLLED COPY FOR INSPECTION AND USE

subject can be tested again (second trial) wearing the smaller size inlet covering.

4.3.1.2.3. A subject failing to achieve a trial pass in either of the sizes available for testing is considered to be a face-size failure.

4.3.1.3. For PAPRs supplied with loose-fitting, respiratory inlet coverings in three sizes -

4.3.1.3.1. Subjects from panel cells 1 and 2 shall be tested wearing the smaller size initially.

4.3.1.3.2. Subjects from panel cells 3-7 shall be tested wearing the regular/medium size initially.

4.3.1.3.3. Subjects from panel cells 8, 9, and 10 shall be tested wearing the larger size initially.

4.3.1.3.4. If a subject does not achieve a trial pass in the first inlet covering size evaluated, the subject will be retested in the next available size (second trial). A subject failing in the smaller size inlet covering, can try the medium size (second trial), and then larger size (third trial) inlet covering. A subject failing to achieve a trial pass in the medium size inlet covering can try the smaller and larger size inlet coverings. A subject failing in the larger size inlet covering can try the medium, then the smaller size inlet covering. A subject failing to achieve a trial pass in any of the sizes available for testing is considered a face-size failure.

4.3.1.3.5. The test administrator may determine whether or not third trials are needed for test completion. For a subject failing to achieve a trial pass in the small or medium size inlet covering, trying the large size may not be necessary since the large size may be obviously too big for the subject. For a subject failing to achieve a trial pass in the large and medium size inlet covering, trying the small size may not be necessary since the small size may be obviously too small for the subject.

5. PROCEDURE

5.1. Chamber Set-up

5.1.1. Turn on air handling unit with sufficient airflow to maintain the proper corn oil concentration.

5.1.2. Turn on vacuum pump for laser photometers

UNCONTROLLED COPY FOR INSPECTION AND USE

UNCONTROLLED COPY FOR INSPECTION AND USE

5.1.3. Turn on mixing fans to 6.1 volts.

5.1.4. Turn on air compressor for corn oil generators to maintain the proper corn oil concentration. Corn Oil Challenge Concentration = 30 to 40 mg/m^3.

5.1.5. Turn on laser photometers.

5.1.6. Turn on SMPS and warm up for 15 minutes.

5.1.7. Turn on DustTrak.

5.1.8. Allow 30 minutes for the chamber concentration to stabilize.

5.1.9. Use the DustTrak to monitor the chamber concentration.

5.1.10. Adjust the air pressure at the generators regulator to establish the corn oil concentration of 30 to 40 mg/m^3.

5.1.11. Use the SMPS according to the manual to determine the particle size. The correct size should be 0.4 to 0.6 µm with a geometric standard deviation of less than 2.0.

5.2. <u>Conducting the Corn Oil Test</u>

5.2.1. The Users Instructions (UI) provided with the test samples shall be reviewed by all test facility personnel. Test subjects will be taught by the test facility administrator on the areas of manufacturer's size selection, donning, doffing and procedures related to the accessories as specified by the UI.

5.2.2. Test subject training will be conducted by test facility personnel based on the manufacturer's users' instructions. Each test subject shall perform an unassisted donning of the respirator. Self-donning under supervision of the test administrator is permitted to make the appropriate adjustments to the respiratory inlet covering until they are satisfied that they are wearing the respirator in compliance with the manufacturer's users' instructions. Expert donning is not allowed in the conduct of this test.

5.2.3. Subjects will be assigned to a specific photometer and moved to the chamber in groups of four or less based on the number of photometers.

5.2.4. Test subjects entering and leaving the corn oil-charged chamber must enter the vestibule first. Once the outside door is closed, the interior door is opened to allow subjects in the chamber. Once subjects are in the chamber they will be instructed to attach their sample line tubing to their assigned photometer. Chamber concentration is required to be monitored continuously during the entire duration of each individual (face-size trial) Corn Oil test.

5.2.5. Information for each test subject will be recorded in the NIOSH Dynamic Fit

software program. Test Administrator will start the software program and relay the information of time to start the test, exercise, and timing of the exercise being performed.

5.2.6. A fit factor test consists of a set of four two-minute standard exercises. During the test, each human subject will perform the following four exercises for two minutes each in the below listed sequence. Subjects should not touch any portion of the respirator during any part of the testing exercises. Test administrator will give verbal commands to stop and start each exercise.

5.2.6.1. Two (2) minutes nodding up and down and turning head side to side.

5.2.6.2. Two (2) minutes callisthenic arm movements.

5.2.6.3. Two (2) minutes running in place.

5.2.6.4. Two (2) minutes pumping with tire pump.

5.2.7. Instruct the subjects to disconnect sample line from the photometer. Exit the chamber using the vestibule room. Inform the subject to return to the ready line and await further instructions for doffing the respirator or leaving the respirator donned. Subjects that are being reviewed for test failure protocol will remain with respirator donned until instructed to doff.

5.2.8. An overall pass/fail statement for each individual will be recorded by the NIOSH Dynamic Fit software and written on the test data sheet as shown in attachment 8.1.

5.2.9. All comments and observations by test subjects, which are voluntary, will be written on the test data sheet.

5.2.10. If a respirator is identified as a failure upon trial termination, test administrator will conduct failure assessment protocol of the respirator in two phases. First phase is to inspect the respirator while it is still donned on the test subject. Second phase is to inspect the respirator when it is doffed. Post-test failure analysis should consist of inspection of the test subjects eye to eye lens positioning, head harness positioning, head harness strap twists, probe loose, missing or on a molded seal or surface causing seal gap or any other case dependent situations. If noted deficiencies are confirmed with the respirator being improperly probed, reassign another like respirator to the test subject and retest. If the respirator has a serviceable probe but continues to fail, log it as a Corn Oil failure. Only inspect the probe assembly if test results are consistently failing or suddenly failing after successful exercise results are indicated. Probe failures such as ripped face blank material or inadequate probe sealing areas are cause for reanalysis of the determined probe entry point.

6. <u>PASS/FAIL CRITERIA</u>

UNCONTROLLED COPY FOR INSPECTION AND USE

6.1. The requirement for passing this test is set forth in 42 CFR Part 84, Subpart K, Section 84.176(b).

6.2. The number of face-size failures will not exceed four.

6.3. If an overall pass is achieved, but three subjects report the same issue about the comfort of the facepiece, the test will be considered a failure.

6.4. Fit factor for loose-fitting PAPR -

6.4.1. For each face-size trial, an overall average fit factor of 500 must be achieved.

6.4.2. A minimum average fit factor of 500 must be achieved during each of the subject exercises which comprise a single face-size trial.

7. RECORDS/TEST SHEETS

7.1. All test data collected will be recorded on the appropriate Determination of Quantitative Corn Oil Fit Test data sheet.

8. ATTACHMENTS

8.1. Example Data Sheet - PAPR Fit Corn Oil Test Data Sheet – Page 1

8.2. Example Data Sheet - PAPR Fit Corn Oil Test Data Sheet – Page 2

8.3. Photographs

8.3.1. Figure 1. Photograph of Laser Photometer

8.3.2. Figure 2. Photograph of Aerosol Generator

8.3.3. Figure 3. Photograph of DustTrak II Aerosol Monitor

8.3.4. Figure 4. Photograph of Scanning Mobility Particle Sizer

8.3.5. Figure 5. Photograph of Charged Test Chamber

8.3.6. Figure 6. Photograph of Sliding Calipers

8.3.7. Figure 7. Photograph of Spreading Calipers

8.3.8. Figure 8. Photograph of Front View of Sample Probe

8.3.9. Figure 9. Photograph of Side View of Sample Probe

8.4. Figure 9. Anthropometric Measurements

8.5. Figure 10. Diagram, NIOSH Panel

UNCONTROLLED COPY FOR INSPECTION AND USE

8.1. Example Data Sheet – Page 1

National Institute for Occupational Safety and Health
Respirator Branch
Test Data Sheet

NIOSH

Task Number: TN-XXXXX
Test: PAPR Fit Testing (Cornoil)
Manufacturer: Company Name
Item Tested:
Minimum Fit Factor:

Reference No.: CFR 84.176(b)
STP No.:

Subject	Face Size	Facepiece	Result	
			Trial 1	Trial 2

Overall Result: ______________________

Test Operator: ______________________ **Date:** _____________
Engineering Technician

Comments:

Was all equipment verified to be in calibration throughout testing?

Test Operator Signature: ________________________

UNCONTROLLED COPY FOR INSPECTION USE

8.2. Example Data Sheet – Page 2

Fit Testing Report

Name	GAGEPak #		Calibration Due
TSI Photometer			
TSI Photometer			

8.3. Photographs

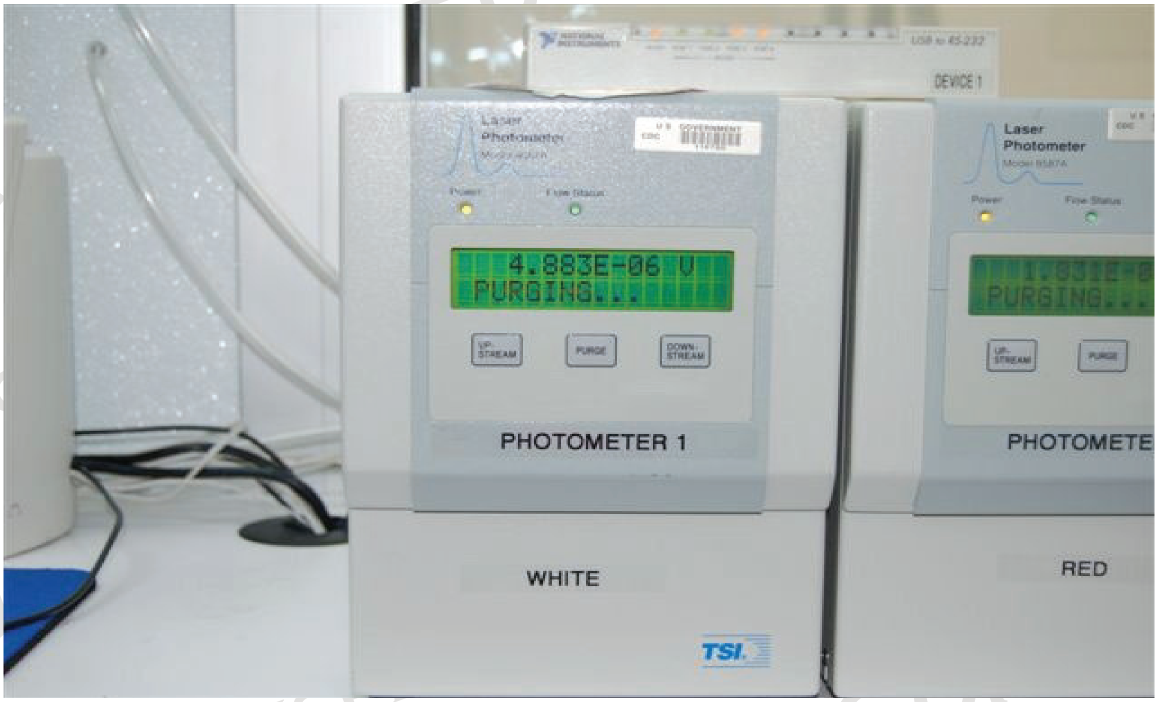

Figure 1: Laser Photometer, Model 8587A

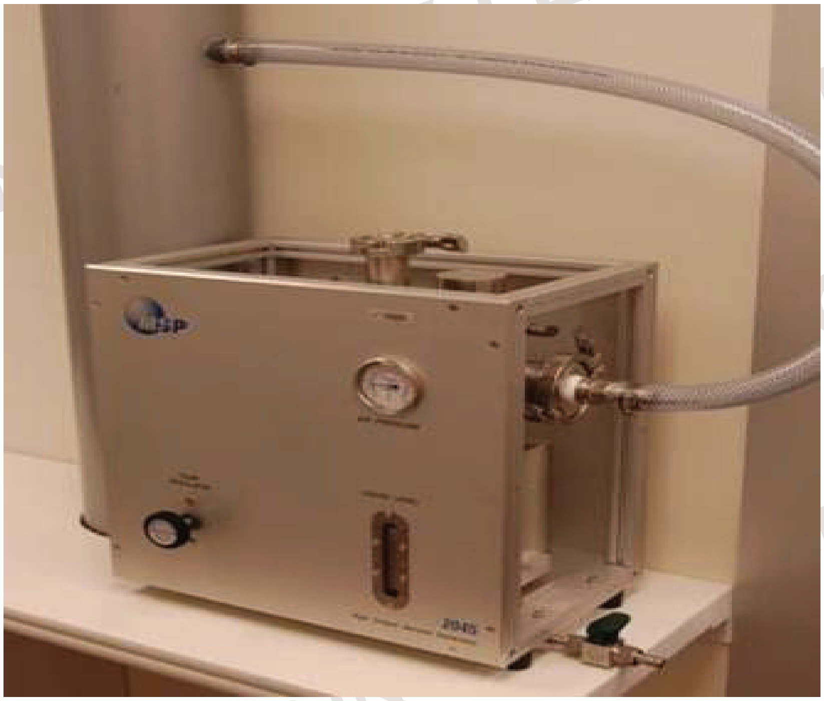

Figure 2: Aerosol Generator

UNCONTROLLED COPY FOR INSPECTION USE

8.3. Photographs

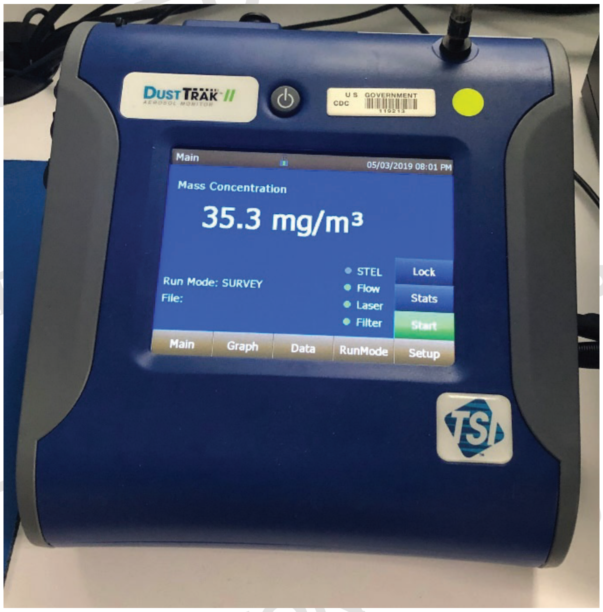

Figure 3: DustTrak II Aerosol Monitor

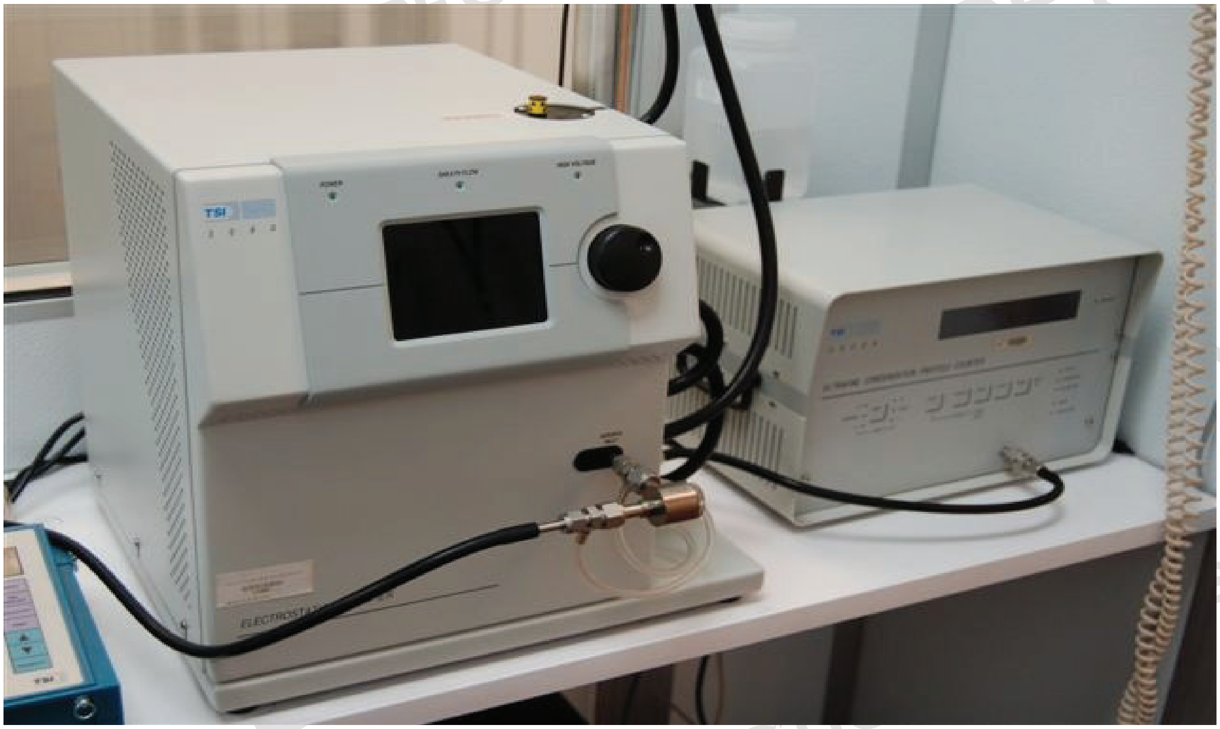
Figure 4: Scanning Mobility Particle Sizer

8.3. Photographs

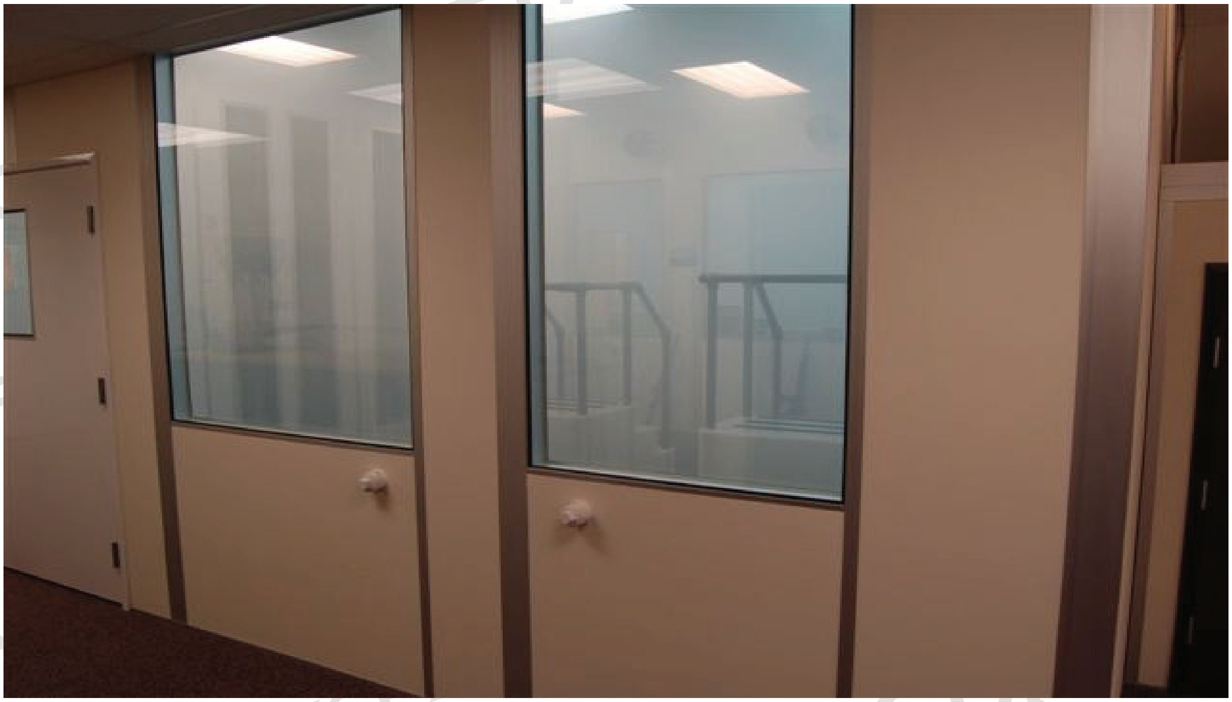

Figure 5: Charged Test Chamber

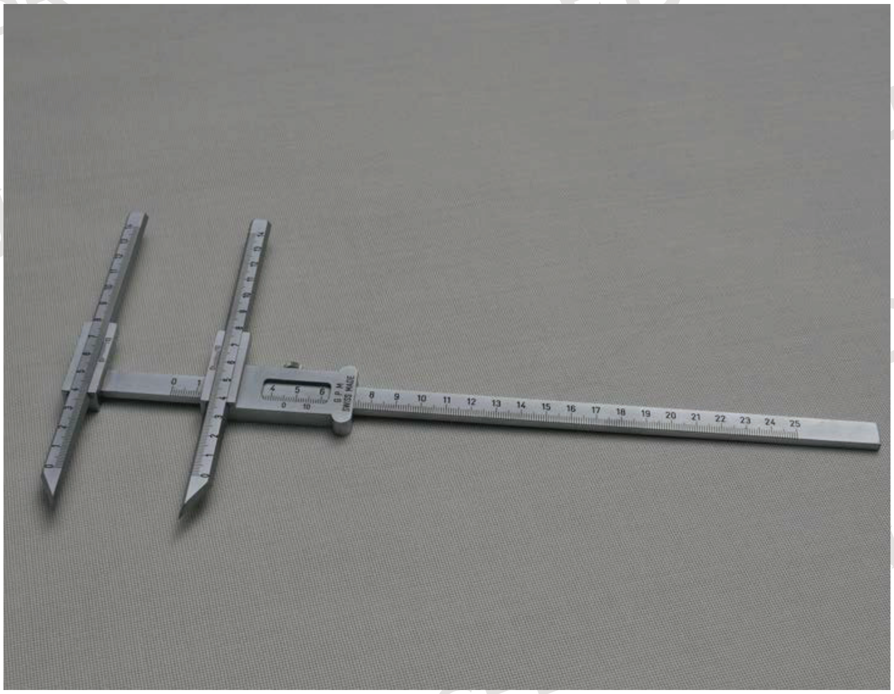

Figure 6: Sliding Calipers

UNCONTROLLED COPY FOR INSPECTION AND USE

8.3. Photographs

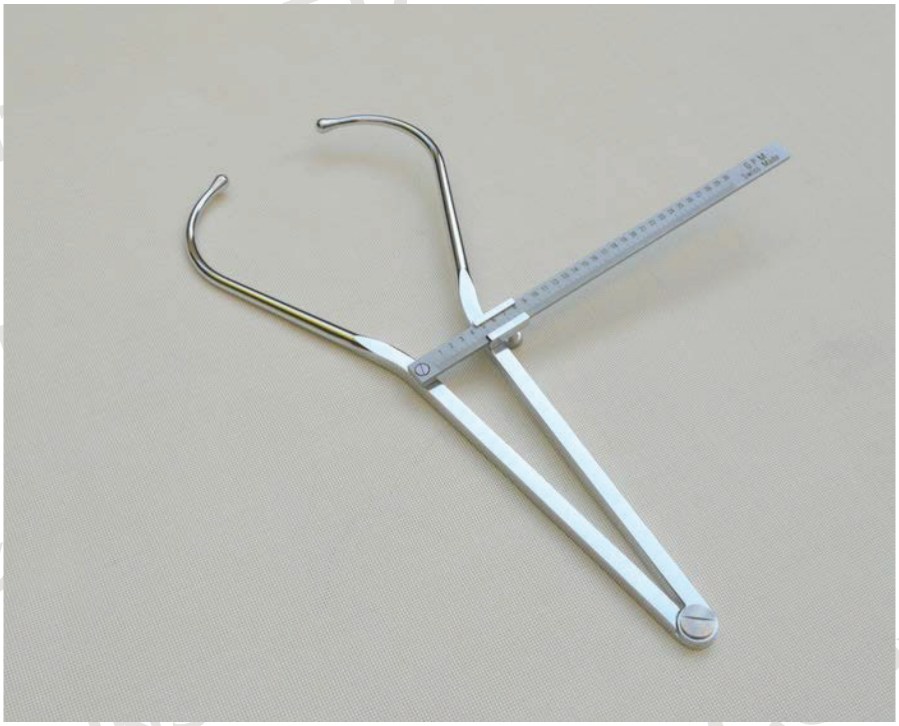

Figure 7: Spreading Calipers

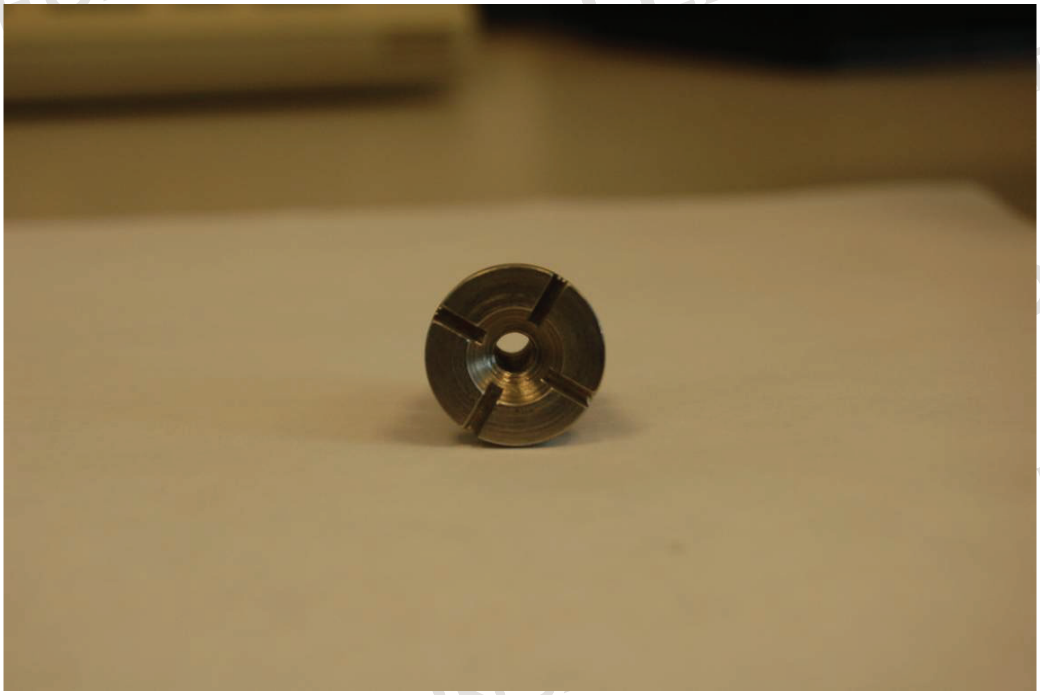

Figure 8: Front View of Sample Probe

8.3 Photographs

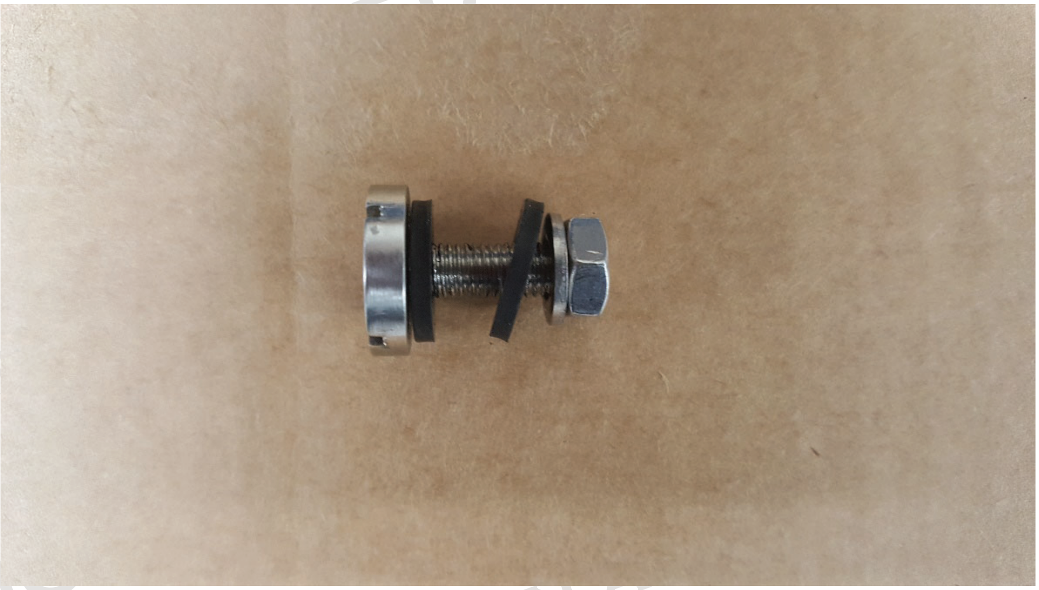

Figure 9. Side View of Sample Probe

UNCONTROLLED COPY
FOR INSPECTION AND USE
UNCONTROLLED COPY
FOR INSPECTION AND USE
UNCONTROLLED COPY
FOR INSPECTION AND USE

8.4. Anthropometric Measurements

Description	Definition	Diagram
**Bizygomatic Breadth**	Maximum horizontal breadth of the face as measured with a spreading caliper between the zygomatic arches.	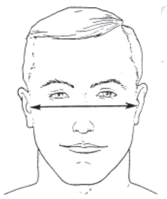
**Menton–Sellion Length**	Distance as measured with a sliding caliper in the midsagittal plane between the menton landmark and the sellion landmark.	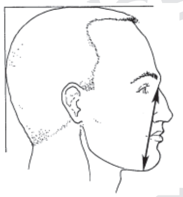

Figure 9: Anthropometric Measurements

UNCONTROLLED COPY FOR INSPECTION AND USE

8.5. NIOSH Panel

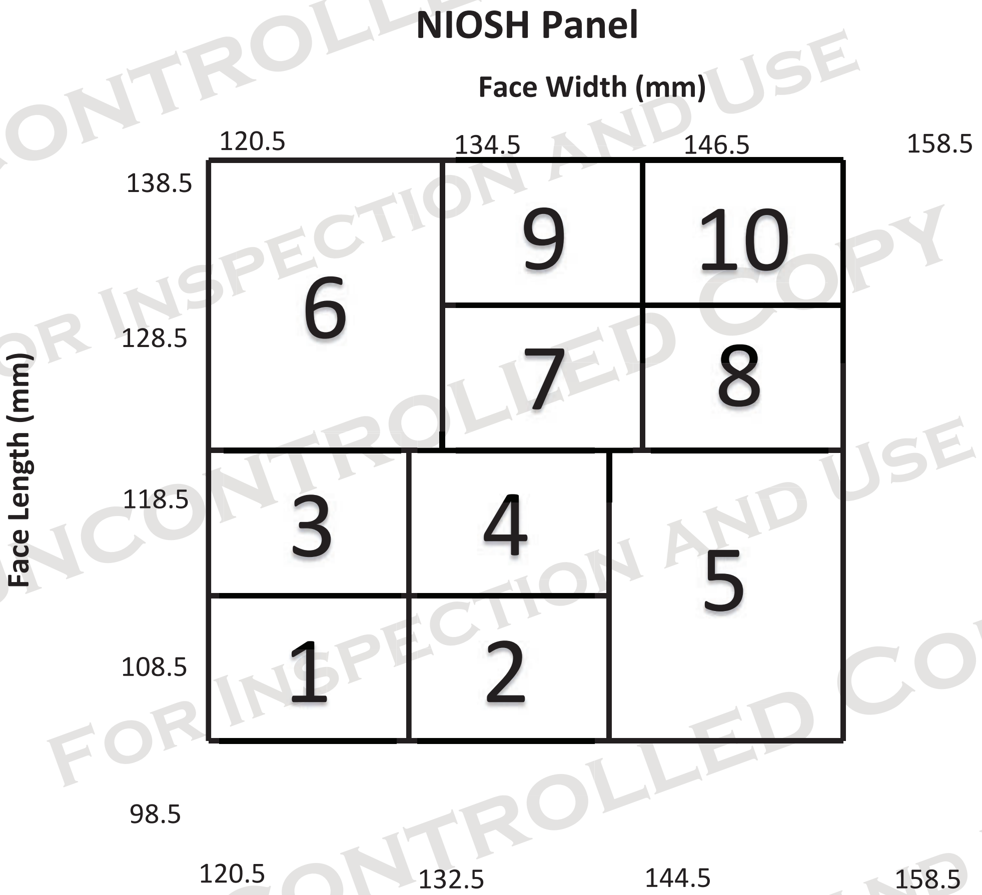

Figure 10: NIOSH Bivariate Panel (NIOSH PANEL)

UNCONTROLLED COPY FOR INSPECTION AND USE

Revision History

Revision	Date	Reason for Revision
0.0	18 March 2020	Original release

UNCONTROLLED COPY FOR INSPECTION AND USE
UNCONTROLLED COPY FOR INSPECTION AND USE
UNCONTROLLED COPY FOR INSPECTION AND USE
UNCONTROLLED COPY FOR INSPECTION AND USE

National Institute for Occupational Safety and Health
National Personal Protective Technology Laboratory
626 Cochrans Mill Road
Pittsburgh, PA 15236

Procedure No. CVB-APR-STP-0081	Revision: 0.0	Date: 23 March 2020

UNCONTROLLED COPY FOR INSPECTION AND USE

DETERMINATION OF PARTICULATE FILTER EFFICIENCY LEVEL AGAINST SOLID PARTICULATES FOR POWERED AIR-PURIFYING RESPIRATORS (PAPRs), SERIES PAPR100-N, STANDARD TESTING PROCEDURE (STP)

1. PURPOSE

This procedure establishes the means for ensuring that the particulate filtering efficiency of PAPR100-N series filters meet the requirements set forth in 42 CFR, Part 84, Subpart K, Section 84.180. These filters or filter cartridges may be integral to respirator construction; mounted individually, or in sets of up to three; used in conjunction with filters, cartridges and canisters for half-mask, full facepieces, hoods, and helmets.

2. GENERAL

This STP describes the test method to be used for the Determination of Particulate Filter Efficiency Level Against Solid Particulates for Powered, Air-purifying Respirators, Series PAPR100-N, test procedure in sufficient detail that a person knowledgeable in the appropriate technical field can conduct the test and determine whether, or not the product passes the test.

3. EQUIPMENT/MATERIALS

3.1. The list of necessary test equipment and materials follows.

3.1.1. TSI Model 8130 Automated Filter Tester or equivalent instrument. Air flow control accuracy is 2% of full scale. Pressure measurement accuracy is 2% of full scale. Penetrations can be measured to 0.001%, efficiencies to 99.999%.

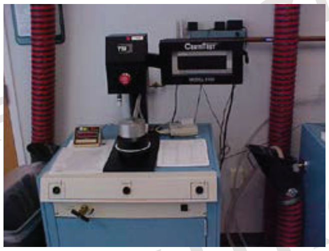

3.1.2. Microbalance accurate to 0.0001 grams (g).

3.1.3. Type A/E glass filters, 102 mm diameter, high efficiency filters with a 1 micrometer pore size.

3.1.4. Timer (accurate to 0.01 percent).

3.1.5. 2% sodium chloride solution in distilled water (NaCl).

3.1.6. Temperature and humidity chamber capable of maintaining 38 ±2.5 °C and 85 ± 5% relative humidity.

3.1.7. Respirator filter holder supplied for specific manufacturer type which is compatible with TSI filter tester. NIOSH will not be obligated to use these holders for actual certification testing. All manufacturer test fixtures must be correlated with the NIOSH test method.

3.1.8. Thermal printer (supplied with TSI 8130) or optional data acquisition system.

3.1.9. TSI, Green Line paper, part number 813010. Lot number must be included on each box. Each lot number must include the "Penetration vs. Resistance graph".

4. TESTING REQUIREMENTS AND CONDITIONS

4.1. Prior to beginning any testing, confirm that all measuring equipment employed has been calibrated in accordance with the testing laboratory's calibration procedure and schedule. All measuring equipment utilized for this testing must have been calibrated using a method traceable to recognized international standards when available.

4.1.1. Respirator filters and filter cartridges shall be tested as follows. Filters used in conjunction with PAPR100-Ns, and odd or unusually shaped filters may be tested on a test fixture provided by the applicant.

4.1.2. If a test fixture is supplied by the applicant, the test fixture shall have a serial number or other unique, easily referenced identifier permanently etched, engraved, or affixed.

5. PROCEDURE

5.1. Respirator filters will be challenged by a NaCl aerosol at 25 ±5 °C and a relative humidity of 30 ±10% that has been neutralized to the Boltzmann equilibrium state. The particle size distribution will be a count median diameter of 0.075 ± 0.020 micrometer and a geometric standard deviation not exceeding 1.86. Each respirator filter unit will be challenged with an aerosol concentration not exceeding 200 mg/m^3.

5.1.1. The NaCl aerosol concentration will be determined on the days that initial penetration testing is performed by the following gravimetric method and calculated as milligrams per cubic meter (mg/m^3).

5.1.2. Weigh a 102 mm filter to the nearest 0.1 mg., mount in the gravimetric filter holder, subject it to the generated aerosol at 30 Lpm for 40 minutes and reweigh the filter. Use a timer to monitor the duration of the test. Record the pre- and post-weights, time, and average flow rate on the data sheet and calculate the aerosol concentration in mg/m^3 by the following formula:

UNCONTROLLED COPY FOR INSPECTION AND USE

UNCONTROLLED COPY FOR INSPECTION AND USE

$$\text{Concentration (C) in mg/m}^3 = \frac{(W2 - W1)}{(Q / 1000)\ (T)}$$

Where:

W1 = Initial filter weight in mg
W2 = Final filter weight in mg
Q = Flowrate in liters per minute
T = Test time in minutes

With a flowrate of 30 Lpm for 40 minutes, the above formula simplifies to:

$$C = \frac{W2 - W1}{1.2}$$

5.1.3. Use the following formula to calculate the test duration:

$$\text{T in minutes} = \frac{(\text{mg load})\ (1000\ \text{L / m3})}{(C)\ \ (Q)}$$

Where:

C = Concentration in mg/m3 from 5.1.2.
Q = Flow rate for test in Lpm

5.1.4. The upstream and downstream photometer readings are used for monitoring stability and for calculating a photometer correlation factor (CF). The correlation factor is determined with an empty filter holder and is calculated internally as shown below:

$$CF = \frac{\text{Downstream Photometer Voltage} - \text{Downstream Background Voltage}}{\text{Upstream Photometer Voltage} - \text{Downstream Background Voltage}}$$

The correlation factor is used by the software to express the upstream photometer signal in terms of the downstream photometer signal.

5.1.5. The NaCl particle size distribution shall be verified using “green line” filter discs supplied by TSI with a known penetration range. Graphs of penetration vs. resistance for two sheets and five sheets of stacked filter discs are supplied with each lot of the standard filters, with a central line and upper and lower lines representing the expected penetration range at a given resistance. The test data should fall within an acceptance zone having boundaries defined by the upper and lower curves on the graphs. The standard filter test using both 2 sheets and 5 sheets will be run at least once in each 8 hour test period to verify that the aerosol distribution is within the acceptance zone.

5.2. Respirator filters will be pre-conditioned at 85 ± 5% relative humidity and 38 ±2.5°C for

25 ±1 hours. After conditioning, the filters shall be sealed in a gas tight container and tested within 10 hours.

5.3. Filters will be mounted and sealed on holders to prevent leakage around the filter holder. Single air purifying respirator filters will be tested at a challenge flow rate of 85± 4 Lpm. Filters used as pairs on a respirator are tested using a single filter of the pair at 42.5 ± 2 Lpm challenge flow rate. Filters used in threes are tested using a single filter of the set at 28.3 ± 1 Lpm challenge flow rate.

5.3.1. The challenge flow rate must be checked for stability for at least 30 seconds prior to testing.

5.4. A sample of 20 filter units will be tested against the NaCl aerosol. Three filters will be loaded until the aerosol mass loading levels as shown in the table below are reached and evaluated to determine the method for the remaining 17 filters. This is the mass amount of NaCl aerosol that has contacted the filter.

Number of Filters In Respirator Configuration	Aerosol Mass Loading Level
Single	200 ± 5 mg.
Double	100 ± 5 mg.
Triple	66.7 ± 5 mg.

5.4.1. Type 1. If preliminary testing of all three initial test filters consistently results in a straight line (Figure 3), for the remaining 17 filters, record the initial penetration reading.

5.4.2. Type 2. If filter testing of all three initial test filters consistently results in a curve which indicates increased efficiency during the complete run (Figure 3), for the remaining 17 filters, record the initial penetration reading.

5.4.3. Type 3. If filter testing of all three initial test filters consistently results in decreased efficiency over time (Figure 3), load the remaining 17 filters with NaCl to the level specified in the table above and record the maximum penetration reading.

5.4.4. Type 4. If filter testing of all three initial test filters consistently results in increased efficiency, then a decrease in efficiency, and then flattens out during the remainder of the complete run (Figure 3), for the remaining 17 filters, record the maximum penetration reading after reaching and maintaining a flat line for a period of 20 minutes following the decreasing segment in efficiency.

5.4.5. For any other filter type, determine loading at which maximum penetration consistently occurs and test at that loading value for the remaining 17 filters.

5.4.6. If any one of the 20 filters have a penetration greater than 0.030%, further testing of that filter will be terminated. Any filter that exceeds the specified limit shall

UNCONTROLLED COPY FOR INSPECTION AND USE

be remounted and retested to ensure that leakage was not caused by a mounting leak If retesting eliminates the excessive leakage and testing has gone beyond the initial penetration, that sample will be considered an invalid sample, and another tested in its place.

5.5. The penetration of the first three filters will be measured, recorded, and printed at approximately 1-minute intervals during the test period. The highest penetration observed throughout the test of each filter will be recorded as the maximum penetration of that filter.

5.6. Determine and record on the data sheet the maximum filter penetration for each of the 20 filters.

6. PASS/FAIL CRITERIA

6.1. The requirement for passing this test is set forth in 42 CFR, Part 84, Subpart K, Section 84.180.

6.2. The minimum efficiency for each of the 20 filters shall be determined and recorded and shall be equal to or greater than 99.97 %.

6.3. For the sample of 20 filters or filter cartridges to demonstrate acceptable performance, each filter shall meet or exceed the specified minimum efficiency level at the end point of the test.

7. RECORDS/TEST SHEETS

7.1. Record the test data in a format that shall be stored and retrievable.

8. ATTACHMENTS

8.1. Filtration Efficiency versus Time Example Plots

8.2. Example Data Sheet

8.3. Photograph of TSI 8130 CertiTester with chuck open

8.4. Photograph of TSI 8130 CertiTester with the chuck closed

UNCONTROLLED COPY FOR INSPECTION AND USE

8.1. Filtration Efficiency versus Time Example Plots

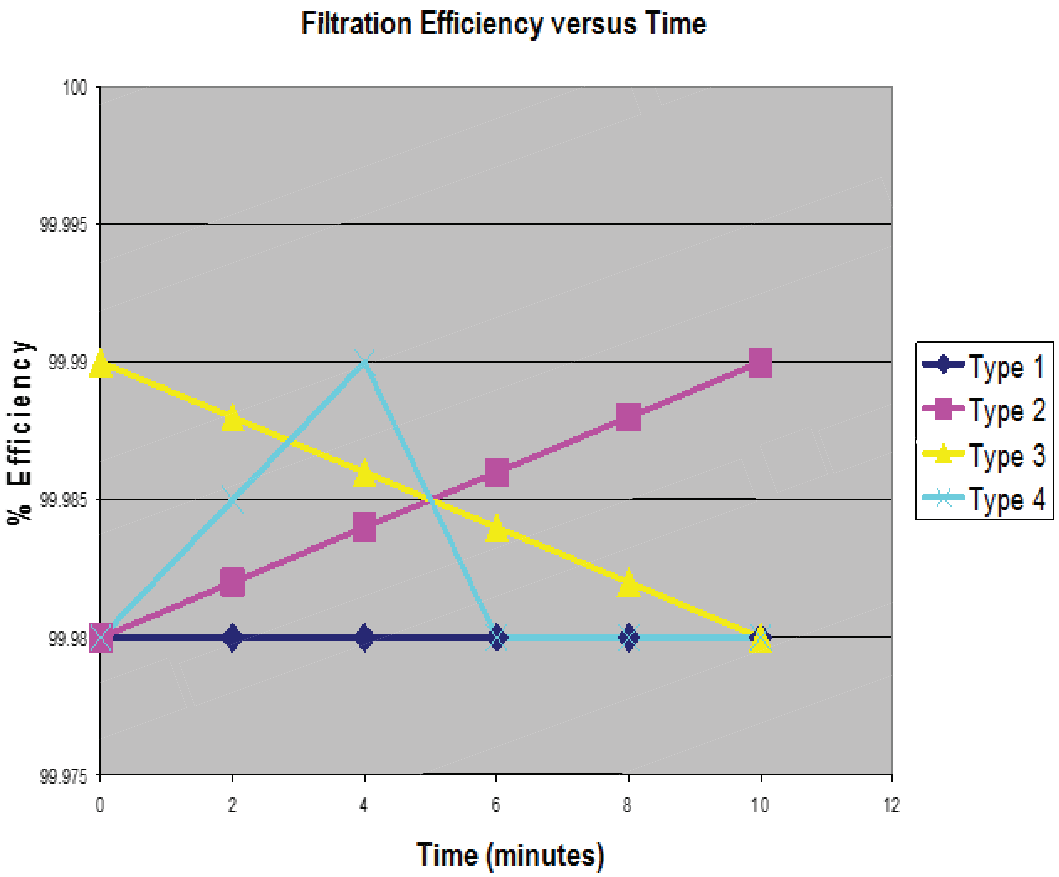

UNCONTROLLED COPY FOR INSPECTION AND USE

8.2. Example Data Sheet

National Institute for Occupational Safety and Health
Respirator Branch
Test Data Sheet

NIOSH

Task Number: TN-XXXXX
Test: Sodium Chloride (NaCl) PAPR100-N
Manufacturer: Company Name
Item Tested:

Reference No.: CFR 84.180
STP No.:

Filter	Flow Rate	Initial Filter Resistance	Maximum Allowable Percent Leakage	Initial Percent Leakage	Maximum Percent Leakage	Result
1	85	7.9	0.03	.002	.002	PASS
2	85	8.2	0.03	.002	.002	PASS
3	85	8.1	0.03	.001	.003	PASS
4	85	7.8	0.03	.003	.004	PASS
5	85	7.8	0.03	.001	.003	PASS
6	85	8.3	0.03	.002	.002	PASS
7	85	8.2	0.03	.001	.002	PASS
8	85	8.1	0.03	.002	.003	PASS
9	85	8.2	0.03	.001	.002	PASS
10	85	8.2	0.03	.001	.002	PASS
11	85	8.3	0.03	.002	.003	PASS
12	85	7.8	0.03	.001	.001	PASS
13	85	7.9	0.03	.001	.001	PASS
14	85	8.3	0.03	.002	.002	PASS
15	85	8.2	0.03	.001	.002	PASS
16	85	7.9	0.03	.002	.002	PASS
17	85	8.2	0.03	.002	.003	PASS
18	85	7.9	0.03	.001	.002	PASS
19	85	8.2	0.03	.000	.002	PASS
20	85	7.8	0.03	.002	.003	PASS

Overall Result: PASS

Signature: ____________________________ **Date:**____________________
Engineering Technician

UNCONTROLLED COPY FOR INSPECTION USE

UNCONTROLLED COPY FOR INSPECTION AND USE

8.3. Photograph of TSI 8130 with chuck open

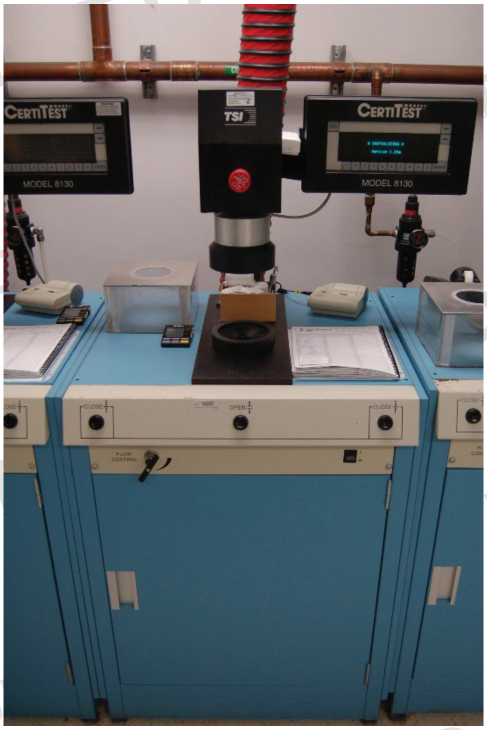

8.4. Photograph of TSI 8130 with chuck closed

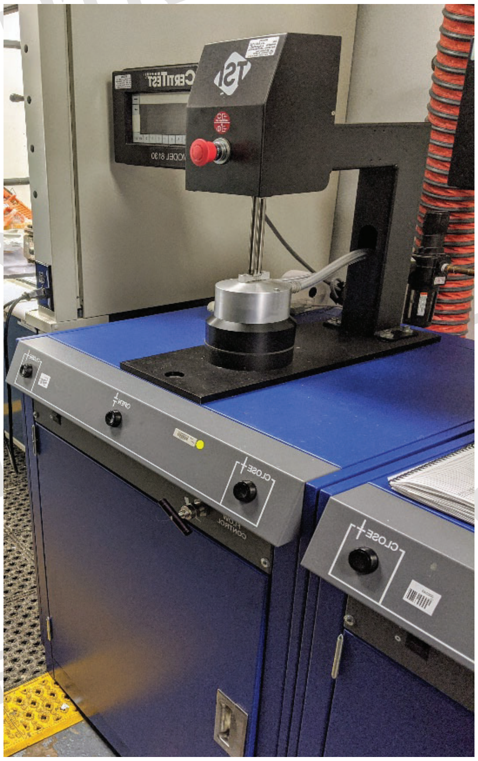

Revision History

Revision	Date	Reason for Revision
0.0	23 March2020	Original release

UNCONTROLLED COPY
FOR INSPECTION AND USE
UNCONTROLLED COPY
FOR INSPECTION AND USE
UNCONTROLLED COPY
FOR INSPECTION AND USE
UNCONTROLLED COPY
FOR INSPECTION AND USE

UNCONTROLLED COPY FOR INSPECTION AND USE

DETERMINATION OF LOW FLOW WARNING DEVICE SOUND LEVEL ON POWERED AIR PURIFYING RESPIRATOR (PAPR), SERIES PAPR100, STANDARD TESTING PROCEDURE (STP)

1. PURPOSE

This test establishes the method for ensuring that the level of protection provided by the Low Flow Warning Devices Sound Level, series PAPR100, meet the minimum requirements set forth in 42 CFR, Part 84, Subpart K, Section 84.171(j)(6).

2. GENERAL

This STP describes the Determination of Low Flow Warning Device Sound Level on PAPR100s test procedure in sufficient detail that a person knowledgeable in the appropriate technical field can select equipment with the necessary resolution, conduct the test, and determine whether or not the product passes the test.

3. EQUIPMENT/MATERIAL

3.1. The list of necessary test equipment and materials follows:

3.1.1. Noise Dosimeter-- Quest Technologies Noise Pro Series Dosimeter For OSHA use, the dosimeter must have a 5 dB exchange rate, use a 90 dBA criterion level, be set at slow response, and use either an 80 dBA or 90 dBA threshold gate, or a dosimeter that has both capabilities, whichever is appropriate for the evaluation.

3.1.2. Lifesize mannequin.

4. TESTING REQUIREMENTS AND CONDITIONS

4.1. Prior to beginning any testing, confirm that all measuring equipment employed has been calibrated in accordance with the testing laboratory's calibration procedure and schedule. All measuring equipment utilized for this testing must have been calibrated using a method traceable to recognized international standards when available.

4.2. Noise level test must be performed in a location that has a maximum background noise level of no more than 60 dBA.

5. PROCEDURE

5.1. Position the microphones of the Quest NoisePro Dosimeter on each ear of the mannequin. Following the respirator manufacturer's instructions, mount the respirator assembly onto the mannequin.

5.2. Turn PAPR system on and follow the low flow user check, in user manual instructions, to activate the audible alarm.

5.3. Take and record five measurements at the mannequin's ear locations.

5.4. Data Analysis

5.4.1. Average the five readings taken from the left ear.

5.4.2. Average the five readings taken from the right ear.

5.4.3. Average the Left Ear Average and Right Ear Average to get the Overall Average.

6. PASS/FAIL CRITERIA

6.1. The requirement for passing this test is set forth in 42 CFR Part 84, Subpart K, Section 84.171(j)(6).

6.2. If the warning provided is audible only, the minimum sound level must be 80 dBA.

6.3. This test should be done on a minimum of two respirators.

7. RECORDS/TEST SHEETS

7.1. All test data collected will be recorded on the appropriate Determination of Minimum Sound Level for Low Flow Warning Device on PAPR100 Test Data Sheet.

8. ATTACHMENTS

8.1. Example Data Sheet

8.2. Photograph – Mannequin Wearing Sound Meters

UNCONTROLLED COPY FOR INSPECTION AND USE

8.1. Example Data Sheet

Determination of Minimum Sound Level for Low Flow Warning Device "On"
PAPR100 Test Data Sheet

Project No.: ________________________________ Date: __________

Company: ________________________________

Respirator Type: ________________________________

Reference: 42 CFR, Part 84, Subpart K, Section 84.171(j)(6)

Requirement: The average sound level at both ears must be greater than 80 dBA.

Procedure: The respirator is mounted on a mannequin. Five sound level measurements are taken at each ear and averaged. The results for the left and right ears are then averaged to arrive at an overall test average.

Results:
Background Noise:________________**dBA**

	Unit # 1: Left Ear\dBA	Unit # 1: Right Ear\dBA	Unit # 2: Left Ear\dBA	Unit # 2: Right Ear\dBA
1.	_______	_______	_______	_______
2.	_______	_______	_______	_______
3.	_______	_______	_______	_______
4.	_______	_______	_______	_______
5.	_______	_______	_______	_______
**Left Ear Average:**	_________		_________	
**Right Ear Average:**	_________		_________	
**Overall Average:**	_________		_________	

Comments:
____
____
____

Test Engineer: ________________________________ PASS________ FAIL________

UNCONTROLLED COPY FOR INSPECTION USE

8.2. Photograph of Sound Test Mannequin Wearing Sound Level Meters

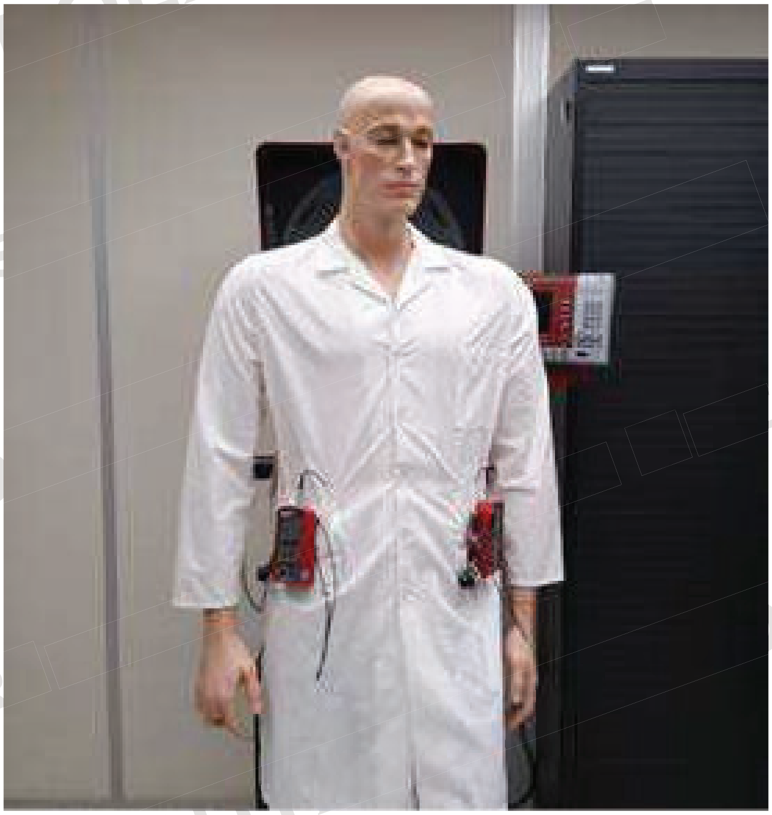

UNCONTROLLED COPY
FOR INSPECTION AND USE
UNCONTROLLED COPY
FOR INSPECTION AND USE
UNCONTROLLED COPY
FOR INSPECTION AND USE

Revision History

Revision	Date	Reason for Revision
0.0	23 March 2020	Original release

UNCONTROLLED COPY
FOR INSPECTION AND USE

UNCONTROLLED COPY FOR INSPECTION AND USE

DETERMINATION OF LOW FLOW WARNING DEVICE
ACTIVATION FOR POWERED AIR-PURIFYING RESPIRATORS, SERIES PAPR100,
STANDARD TESTING PROCEDURE (STP)

1. PURPOSE

This procedure establishes the method for ensuring that the level of protection provided by the low flow warning device requirement for powered air-purifying respirators, series PAPR100, meet the requirements set forth in 42 CFR Part 84, Subpart K, 84.171(j).

2. GENERAL

This procedure describes the Determination of Low Flow Warning Device Activation for Powered Air-Purifying Respirators, Series PAPR100, test procedure in sufficient detail that a person knowledgeable in the appropriate technical field can select equipment with the necessary resolution, conduct the test, and determine whether or not the product passes the test.

3. EQUIPMENT/MATERIAL

3.1. Air-tight chamber – approximately 24 inches by 24 inches by 16 inches with a bolt on door, a 3-inch diameter inlet for accepting breathing tubes and adapter, a 1 inch diameter outlet, and a ¼ inch outlet for a manometer probe.

3.2. Setra Datum 2000 Model 239 digital manometer with an accuracy of ± 0.023 in-H2O or better, or equivalent.

3.3. Teledyne Hastings L-25S Laminar Mass Flow Meter, or equivalent.

3.4. Spencer 075-1/3 Centrifugal Blower vacuum source, or equivalent.

3.5. Anthropometric Headform, in accordance with ISO 16900, size medium, or equivalent.

4. TESTING REQUIREMENTS AND CONDITIONS

4.1. Prior to beginning any testing, confirm that all measuring equipment employed has been calibrated in accordance with the testing laboratory's calibration procedure and schedule. All measuring equipment utilized for this testing must have been calibrated using a method traceable to recognized international standards when available.

4.2. Testing shall be conducted on as-received respirators at 25 ± 2.5 degrees Celsius (°C) and adhere to User's Instructions.

4.3. Three complete respirator systems will be evaluated for low flow warning device activation.

4.4. Determination of Low Flow Warning Device Visibility will be completed for any respirator with a visual warning device. The standard testing procedure is described in CVB-APR-STP-0087.

4.5. Determination of Low Flow Warning Device Sound Level on Series PAPR100 will be completed for any respirator with an audible warning device, where the warning provided is audible only, or other warnings are not readily apparent. The standard testing procedure is described in CVB-APR-STP-0085.

5. PROCEDURE

5.1. Prior to any testing, the respirator shall be evaluated per the user check instructions in the User's Manual to ensure that the low flow warning(s) activate as designed.

5.2. Test setup for low flow warning device activation for continuous flow powered air-purifying respirators

5.2.1. The test chamber outlet is connected to the mass flow meter inlet. The mass flow meter outlet is connected to the vacuum blower inlet. A flow control valve is placed between the mass flow meter and vacuum blower. The vacuum blower vents to atmosphere. Ensure that all pipe lengths are sufficient to maintain laminar flow.

5.2.2. Mount the respirator to the headform in the as-worn configuration following all pertinent User's Instructions.

5.2.3. Connect the headform and respirator to the chamber.

5.2.3.1. On units with breathing tubes, the breathing tube is placed through the chamber inlet port and the blower is placed outside of the chamber. The headform and respirator are placed inside the chamber. Seal the chamber inlet around the breathing tube.

5.2.3.2. On units without breathing tubes, the headform trachea outlet tube is placed through the chamber inlet port and the headform and respirator are placed outside the chamber. Seal the chamber inlet around the headform outlet tube.

5.2.3.2.1. Loose fitting respiratory inlet coverings may be adjusted to capture air-flow that would otherwise exit the respirator.

5.2.4. Connect the digital manometer to the pressure tap on the headform. Pressure is measured at a pitot ring positioned 25 mm inside of the trachea inlet.

5.2.4.1. Ensure the manometer has a reading of zero at ambient conditions, and adjust if necessary.

UNCONTROLLED COPY FOR INSPECTION AND USE

5.2.5. Close the chamber door and ensure the chamber is sealed.

5.2.6. Turn on the PAPR and the vacuum blower. Ensure that no air flow warnings are present on the respirator.

5.2.7. Adjust the flow control valve until manometer has a reading of zero.

5.2.8. Restrict the flow to the respirator. This is done by incrementally adding restriction to the inlet of the respirator, such as attaching small pieces of adhesive tape or a similar flow restricting item.

5.2.8.1. While restricting flow to the respirator, adjust the flow control valve to ensure that the manometer continuously reads zero.

5.2.9. Add increased flow restriction to the respirator inlet until the low flow warning device activates. Record the maximum airflow which activates the low flow warning device.

5.2.10. Turn off the PAPR100 and vacuum blower.

6. <u>PASS/FAIL CRITERIA</u>

6.1. The criterion for passing this test is set forth in 42 CFR Part 84, Subpart K, Section 81.171(j).

6.1.1. The low flow warning must actively and readily indicate when flow inside the respiratory inlet covering falls below the minimum required air flow. The minimum air flow shall be 115 LPM for tight-fitting PAPR100 and 170 LPM for loose-fitting PAPR100.

6.1.2. Any warning must be detectable by the wearer without any intervention by the wearer.

6.1.3. Warning devices must be configured so that they may not be de-energized while the blower is energized.

6.1.4. Any warnings which require different reactions by the wearer must be distinguishable from one another.

7. <u>RECORDS/TEST SHEETS</u>

7.1. Record the test data in a format that shall be stored and retrievable.

UNCONTROLLED COPY FOR INSPECTION AND USE

Revision History

Revision	Date	Reason for Revision
0.0	23 March 2020	Original Release

UNCONTROLLED COPY FOR INSPECTION AND USE

UNCONTROLLED COPY FOR INSPECTION AND USE

DETERMINATION OF COMMUNICATION PERFORMANCE TEST FOR SPEECH CONVEYANCE AND INTELLIGIBILITY OF POWERED AIR-PURIFYING RESPIRATOR (PAPR) SERIES PAPR100 STANDARD TESTING PROCEDURE

1. PURPOSE

1.1. This test establishes the method for ensuring that the level of speech conveyance and intelligibility provided by the Communication Performance Test on Powered Air-Purifying Respirator (PAPR) Series PAPR100 meet requirements set forth in 42 CFR Part 84, Subpart K, Section 84.181.

1.2. The purpose of this test is to quantify the performance of a respirator in transmitting intelligible speech of a human test subject. This is accomplished by determining a listener's ability to comprehend single-syllable words spoken by a subject wearing the respirator.

2. GENERAL

2.1. This STP describes the Communication Performance Tests for Speech Conveyance and Intelligibility of a PAPR100 in sufficient detail that a person knowledgeable in the appropriate technical field can select equipment with the necessary resolution, conduct the test, and determine whether or not the product passes the test.

2.2. The Communication Performance Test shall be performed using the Modified Rhyme Test (MRT) to evaluate a listener's ability to comprehend single-syllable words from a speaker. Both listeners and speakers shall be in combinations of masked and unmasked postures during the MRT to provide an indication of speech transmission and intelligibility.

2.3. This test is considered a human factors test that requires participation of 8 test subjects to quantify the overall performance rating of a PAPR100 system.

2.4. Two (2) Test Administrators are necessary to properly conduct this test.

3. EQUIPMENT/MATERIALS

3.1. Laboratory space that permits an unobstructed 10 +1/-0 ft distance between test speaker and the listener group when facing one another. Refer to Figure 1. for the subject and equipment positioning for the Modified Rhyme Test setup.

3.2. Noise Plug, Precision Pink Noise Test Generator (GTC Industries) or equivalent for producing pink noise in the frequency range of 20.0 Hz to 50.0 kHz. Accuracy: 3dB per octave rolloff from 20 Hz to 20 KHz. Pink noise is defined as an audio test signal that

contains all the frequencies in the audio spectrum with equal energy at each octave. Pink noise contains less energy at the higher audio frequencies than at the lower ones - See Figure 1.

3.3. A stereo amplifier used to transmit and amplify the signal for the pink noise background.

3.4. The two loudspeakers positioned midway between the test speaker and listeners. Details of the exact positioning of the speaker equipment are further defined in Section 5.3.2.

3.5. Two Type 2 digital sound level meters (Sper Scientific, LTD, Model 840029 – See Figure 3.) with an "A" weighting decibel scale of 30 to 130 dB or equivalent. One sound level meter will be positioned in front of the Test Speaker and the second sound level meter will be positioned at head level beside listener L2- see Figure 1.

3.6. Sper Scientific, LTD Acoustical calibrator (model 840031) or equivalent is used to calibrate the sound level meters – See Figure 4.

3.7. Twelve word lists from the Modified Rhyme Test (MRT). These twelve word lists are separated onto individual sheets to be used by the test speakers during the test – See Appendix A.

3.8. Test listeners' multiple-choice answer pages on laptops or equivalent. Modified Rhyme Test listener responses are recorded and scored using laptops that have been programmed for this application; otherwise, the manual method using multiple-choice answer sheets can be used – See Example Data Sheet 8.1.

3.9. Sound and/or audiovisual recording device for recording speech.

3.10. <u>Human Test Subjects</u>

3.10.1. Test Administrators shall have successfully completed the CDC/ATSDR Scientific Ethics Training, the DHHS/NIH Human Participant Protections Education for Research Teams, or equivalent course.

3.10.2. At least eight (8) volunteer test subjects are required for this test.

4. <u>TESTING REQUIREMENTS AND CONDITIONS</u>

4.1. Prior to beginning any testing, confirm that all measuring equipment being used has been calibrated in accordance with the testing laboratory's calibration procedure and schedule. All measuring equipment utilized for this testing must have been calibrated using a method traceable to recognized international standards when available.

4.2. Administrator 1 will monitor the test speakers' speech and the actions of the listener group to make sure they are responding to the test speaker.

UNCONTROLLED COPY FOR INSPECTION AND USE

4.3. Administrator 2 will monitor the dBA level of the test speaker and give verbal or nonverbal feedback as to loudness of his/her speech and record the dBA level on the data sheet – see Example Data Sheet 8.2.

4.4. The eight test subjects will be divided into two groups, a listener group comprised of three subjects (at least one female and one male) and a speaker group comprised of five subjects (at least one female and one male).

4.5. All subjects shall be fluent in English. In addition, these subjects shall have no obvious or strong regional or foreign accents.

4.6. The eight test subjects shall be trained in the donning and use of the respirator per manufacturer's instructions by the test administrator.

4.7. Each subject shall be sized and fitted for the respirator per manufacturer's instructions. Where an individual is qualified to wear multiple sizes of the respirator, the subject shall select the respirator size that provides the most comfortable fit.

4.8. The subjects shall have normal hearing.

4.9. The test subjects shall not have any facial hair or conditions that may cause interference with the seal of the respirator facepiece.

4.10. Test Equipment Set-Up.

4.10.1. Room selected to perform the MRT shall be of ample size to comfortably house all the test equipment indicated and the personnel associated with the test. It shall be free of external noise interference.

4.10.2. The loudspeakers shall be positioned opposite each other at a height of approximately 29.5 inches from the floor and 9 +0.5/-0 ft from the center of the sound field between the test speaker and listeners. The laptops will be placed directly in front of the listeners at a convenient distance away – See Figure 1.

5. PROCEDURE

5.1. Fill in the Pretest data on the data sheet – See Example Data Sheet 8.1.

5.2. Record the background noise of the room, assuring no external noise, at the center listener's head position.

5.3. Training of the Test Speakers.

5.3.1. Without the listeners in the room, one subject at a time will be seated in the test speaker's position and given an MRT word list – See Appendix A. A sound level meter will be positioned in front of the test speaker to assess the volume of their voice as the word list is read. The sound level meter shall be set to display "A"-weighted sound levels.

UNCONTROLLED COPY FOR INSPECTION AND USE

5.3.2. Test administrator 1 shall be seated beside the test speaker to monitor the reading of the word list. The administrator shall instruct the test speaker to read, without placing any unusual emphasis on any stimulus word, at a rate of approximately one phrase every 6 seconds, using the introductory phrase "The word is (list word)". In addition, the test speaker shall be directed to communicate each word without using any visual gestures, such as hand signals, and without repeating any of the list words.

5.3.3. Test administrator 2 shall be seated in the center test listener's position and will instruct the test speaker to begin reading the word list at a voice level of 75 to 85 dBA.

5.3.4. During the reading of the word list, test administrator 2 will monitor the "A"-weighted output volume of the speaker and provide feedback as to the loudness of the speaker's performance throughout the reading of the word list. Test administrator 1 will provide feedback to the test speaker regarding the pronunciation of the list words and the rate of performance. Additional training will be done if warranted per the judgment of the test administrators. These procedures will be completed without the use of a respirator and without any background noise above ambient conditions.

5.3.5. Additionally, training with background noise of 60 ± 2 dBA (consisting of pink noise), will also be completed according to the above procedures.

5.3.6. This will be repeated for all subjects in the speaker group.

5.4. Training of the Test Listeners.

5.4.1. The three test listeners are seated facing a single test speaker at a distance of 10 ft. The listeners are seated next to one another with approximately one foot between them. Each listener will be given a laptop with a list of multiple-choice words for recording his or her responses.

5.4.2. An MRT word list will be provided to a trained test speaker. The test speaker will be instructed to communicate the word list to the test listeners as was done during speaker training.

5.4.3. As a group, listeners will be instructed to listen attentively as the test speaker reads 50 words to them each with the introductory phrase "The word is (list word)." The test listeners shall be directed to select, or make a best guess of the word, that was perceived to be spoken from the six possible response words provided to them on the laptops. Test listeners are instructed to provide a "thumbs-up" hand signal to the speaker as a cue to say the next phrase.

5.4.4. During the reading of the word list, administrator 2 will monitor the A-weighted output volume of the speaker at the listening position and provide feedback as to the loudness throughout the reading of the word list. In addition, test

UNCONTROLLED COPY FOR INSPECTION AND USE

administrator 1 will provide the test speaker feedback regarding the rate of performance. Administrator 1 will also monitor the actions of the listener group and provide additional instructions as needed. Test administrators will determine if additional training is required. These procedures will be completed without the use of a respirator and without any added background noise (pink noise) above ambient conditions.

5.4.5. Additionally, training with added background noise of 60 ± 2 dBA (consisting of pink noise), will also be conducted according to the procedures above.

5.5. Conducting the Test.

5.5.1. The noise generator shall be turned on and set to produce 60 ±2 dBA of pink noise, as measured by the sound level meter at the center test listener's head position without listeners present. This value shall be recorded on the Respirator Communication Performance Test Data Collection Sheet.

5.5.2. The three test listeners shall be seated in the listening position with a laptop located conveniently in front of them.

5.5.3. A test speaker will then be positioned in the speaking position and present one complete MRT word list to the listening panel. A different test speaker shall then be used to present the next MRT trial. Test speakers will continue to rotate among the speaker test panel until all trials have been completed.

5.5.4. Data will be obtained without the respirator (unmasked) and with the respirator (masked) worn and operated per the manufacturer's instructions by both speakers and listeners. All conditions shall be randomly assigned, and a different word list shall be used for each test. A test matrix of the MRT conditions is provided in Table 2 to assist the test administrator in establishing the sequence of testing.

5.5.5. Background noise levels shall be monitored at the center test listener's head position and recorded, on the Respirator Communication Performance Test Data Collection Sheet, at the beginning, middle, and end of each MRT session by administrator 2.

5.5.6. The test speakers shall be monitored and recorded during the test to determine if the test speakers conform to the word list specified for each trial by test administrator 1. Test administrator 1 shall also make note of any improperly pronounced or misspoken words by the test speakers.

5.5.7. A total of 10 MRT trials shall be performed; 5 unmasked and 5 masked. This will result in a total of 15 MRT scores (five per listener) for the unmasked condition and 15 scores for the masked condition.

5.6. Data Analysis.

UNCONTROLLED COPY FOR INSPECTION AND USE

5.6.1. Use the Respirator Communication Performance Test Data Collection Sheets to assist with the analysis of the 10 MRT scores for each listener.

5.6.2. The number of correct responses shall be adjusted for chance or guessing made possible by the multiple-choice answers using the equation:

$$\text{Adjusted Score} = \text{Number of Correct Listener Responses} - \frac{\text{Number of Wrong Listener Responses}}{5}$$

5.6.3. Determine the Number of Words Spoken Correctly by the speakers from either the Test Administrators notes or if necessary, by listening to the Audio recorder tapes.

5.6.4. Listener performance on the MRT shall be scored in terms of the percentage of words correctly identified using the equation for both the masked and unmasked posture:

% Correct = (Adjusted Score/Number of Words Spoken Correctly) * 100

5.6.5. Average Unmasked and Average Masked % Correct Scores shall be calculated for each individual listener.

5.6.6. Each individual listener's Average Masked % Correct Score shall be divided by their Average Unmasked % Correct Score to calculate a Performance Rating using the equation:

5.6.7. $\text{Performance Rating (\%)} = \left(\frac{\text{Average Masked \% Correct Score}}{\text{Average Unmasked \% Correct Score}} \right) \text{x} 100$

5.6.8. The performance rating of all listeners shall then be averaged to determine the Overall Performance Rating of the PAPR100 using the following Equation:

$$\text{Overall Performance Rating (\%)} = \frac{\text{Performance Rating (\%)}_{L1} + \text{Performance Rating (\%)}_{L2} + \text{Performance Rating (\%)}_{L3}}{3}$$

6. PASS/FAIL CRITERIA

6.1. The requirement for passing this test is set forth in 42 CFR, Part 84, Subpart K, Section 84.181.

6.2. A candidate PAPR100 must obtain an Overall Performance Rating greater than or equal to 70% to meet the Communication requirement.

7. RECORDS/TEST SHEETS

UNCONTROLLED COPY FOR INSPECTION AND USE

7.1. All test data shall be recorded on the Respirator Communication Performance Test Data Collection Sheets.

8. ATTACHMENTS

8.1. Example Data Sheet - Communication Performance Test Listener Data Sheet

8.2. Example Data Sheet - Communication Performance Test Data Collection Sheet

8.3. Table 1. MTR Trial Matrix

8.4. Figures

8.4.1. Figure 1. Modified Rhyme Test Setup

8.4.2. Figure 2. Photograph of Noise Plug, Precision Pink Noise Generator (GTC Industries)

8.4.3. Figure 3. Photograph of Sper Scientific LTD digital sound level meter, Model 840029

8.4.4. Figure 4. Photograph of Acoustical calibrator (Sper Scientific model 840031)

8.5. Appendix A. Communication Performance Word List Test Sheets

UNCONTROLLED COPY FOR INSPECTION AND USE

8.1. Example Data Sheet – Communication Performance Test Listener Data Sheet

NIOSH Application Number ______________________________ Date: ____________

Respirator Manufacture: ____________________________________ ; MRT/Sheet# __________

Respirator Type: ______________ ; Speakers Respirator Number, if worn __________ (Indicate if unworn)

Listener's Respirator (circle one): Unworn Worn If Worn, Listeners Respirator Number ________________

Listener #:______ Listening Position:_ Speaker #:______ MRT Word List#:________

#			
1	but	bug	bus
	buff	bun	buck
2	kin	kid	kick
	king	kit	kill
3	peak	peach	peas
	peal	peace	peat
4	dig	wig	big
	fig	pig	rig
5	fold	sold	gold
	hold	cold	told
6	kick	lick	sick
	tick	wick	pick
7	path	pack	pass
	pat	pad	pan
8	beat	beak	beach
	beam	bean	bead
9	pot	hot	lot
	not	tot	got
10	fit	hit	bit
	sit	kit	wit
11	sup	sub	sud
	sum	sun	sung
12	dent	tent	rent
	went	sent	bent
13	best	west	nest
	vest	test	rest
14	map	mat	math
	mad	mass	man
15	hop	cop	shop
	mop	pop	top
16	sack	sad	sap
	sag	sat	sass
17	say	pay	may
	gay	way	day
18	heath	heave	heap
	heat	heal	hear
19	tame	came	fame
	same	name	game
20	page	pane	pace
	pave	pale	pay
21	dust	gust	must
	bust	just	rust
22	pun	puff	pup
	pub	pus	puck
23	then	den	ten
	pen	hen	men
24	cuss	cud	cup
	cut	cub	cuff
25	hook	shook	book
	took	cook	look
26	late	lake	lay
	lame	lane	lace
27	wed	fed	bed
	led	shed	red
28	sane	sake	safe
	save	same	sale
29	pit	pin	pig
	pill	pick	pip
30	heel	peel	keel
	feel	eel	reel
31	toil	boil	foil
	coil	oil	soil
32	fig	fizz	fit
	fib	fin	fill
33	mark	bark	dark
	lark	hark	park
34	bash	bat	ban
	back	bath	bad
35	will	hill	kill
	bill	fill	till
36	pale	sale	bale
	gale	male	tale
37	duck	dud	dung
	dun	dug	dub
38	sit	sip	sill
	sick	sin	sing
39	tack	tan	tab
	tang	tam	tap
40	cake	came	Cave
	cane	case	cape
41	fang	bang	hang
	sang	gang	rang
42	law	saw	paw
	jaw	raw	thaw
43	rake	rate	ray
	raze	race	rave
44	dip	dim	din
	dill	did	dig
45	tear	teal	teak
	team	tease	teach
46	tin	fin	sin
	win	pin	din
47	seethe	seek	seen
	seed	seep	seem
48	run	bun	fun
	sun	nun	gun
49	neat	beat	seat
	meat	feat	heat
50	lip	hip	dip
	sip	rip	tip

Score

8.2. Example Data Sheet – Communication Performance Test Data Collection Sheet

TN # : ________________ **MRT Trial # :** _______________ **Speaker ID # :** ____________

Word List # : _______ Please Circle: Masked Unmasked

Number of words correctly spoken by the MRT Speaker: __________

Listener #1 ID: _________________

1. % of Words Answered Correctly = __________________
2. Adjusted Score = ______________________

Listener #2 ID: _________________

1. % of Words Answered Correctly = __________________
2. Adjusted Score = ______________________

Listener #3 ID: _________________

1. % of Words Answered Correctly = __________________
2. Adjusted Score = ______________________

Background Noise (w/o pink noise): ___________

Pink Noise Before Session: ___________

Pink Noise Middle of Session: ___________

Pink Noise After Session: ____________

UNCONTROLLED COPY FOR USE

8.3. Table 2. MRT Trial Matrix

Trial #	Speaker	With or Without Mask	Word list
1	1	No mask	11A
2	2	No mask	3A
3	3	Masked	5A
4	4	Masked	7A
5	5	No mask	4A
6	2	Masked	12A
7	4	No mask	5A
8	1	Masked	1A
9	5	Masked	8A
10	3	No mask	6A

8.4. Diagram 1. Modified Rhyme Test Setup

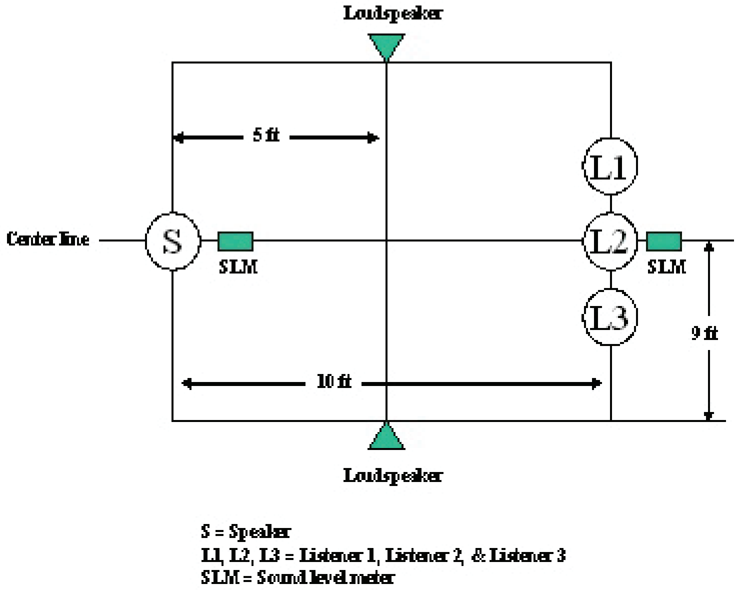

UNCONTROLLED COPY
FOR INSPECTION AND USE

8.5. Figure 1. Noise Plug, Precision Pink Noise Generator (GTC Industries)

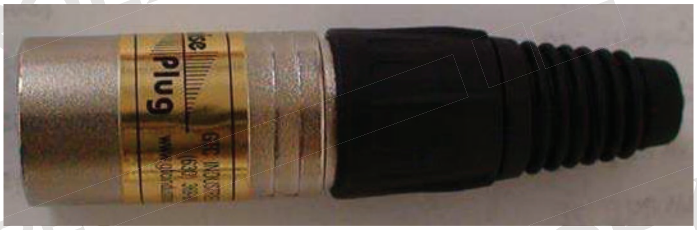

8.6. Figure 2. Sper Scientific LTD digital sound level meter, Model 840029

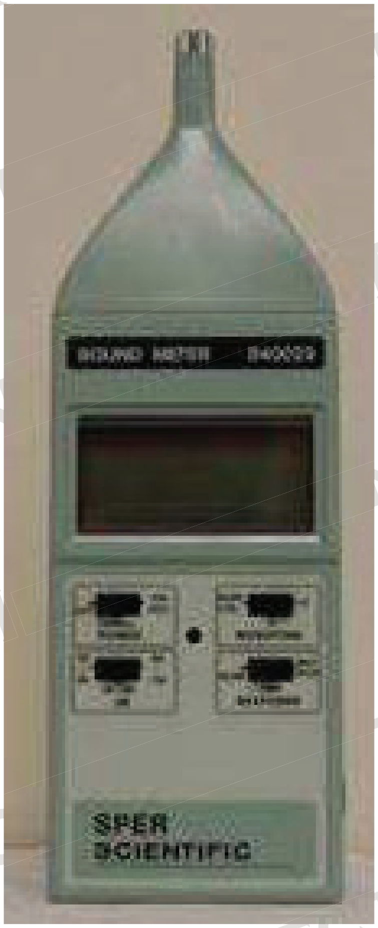

UNCONTROLLED COPY
FOR INSPECTION AND USE

8.7. Figure 3. Acoustical calibrator (Sper Scientific model 840031)

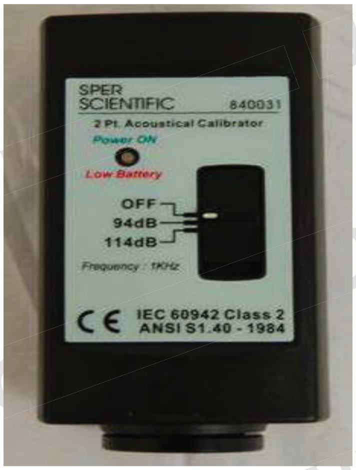

UNCONTROLLED COPY FOR INSPECTION AND USE

8.8. Appendix A. Communication Performance Word List Test Sheets

Appendix A

Modified Rhyme Test Word Lists

UNCONTROLLED COPY
FOR INSPECTION AND USE

List 1A

1. The word is **but**
2. The word is **kit**
3. The word is **peak**
4. The word is **pig**
5. The word is **cold**
6. The word is **sick**
7. The word is **pat**
8. The word is **beat**
9. The word is **hot**
10. The word is **fit**
11. The word is **sung**
12. The word is **sent**
13. The word is **rest**
14. The word is **mat**
15. The word is **top**
16. The word is **sack**
17. The word is **day**
18. The word is **heal**
19. The word is **name**
20. The word is **pay**
21. The word is **must**
22. The word is **pun**
23. The word is **hen**
24. The word is **cud**
25. The word is **book**
26. The word is **late**
27. The word is **led**
28. The word is **same**
29. The word is **pin**
30. The word is **feel**
31. The word is **soil**
32. The word is **fizz**
33. The word is **park**
34. The word is **bash**
35. The word is **till**
36. The word is **male**
37. The word is **dud**
38. The word is **sin**
39. The word is **tack**
40. The word is **case**
41. The word is **bang**
42. The word is **thaw**
43. The word is **ray**
44. The word is **dig**
45. The word is **team**
46. The word is **win**
47. The word is **seek**
48. The word is **bun**
49. The word is **feat**
50. The word is **sip**

UNCONTROLLED COPY FOR INSPECTION AND USE
UNCONTROLLED COPY FOR INSPECTION AND USE
UNCONTROLLED COPY FOR INSPECTION AND USE

UNCONTROLLED COPY FOR INSPECTION AND USE

List 2A

1. The word is **bus**
2. The word is **kick**
3. The word is **peas**
4. The word is **fig**
5. The word is **hold**
6. The word is **lick**
7. The word is **pass**
8. The word is **beach**
9. The word is **pot**
10. The word is **bit**
11. The word is **sub**
12. The word is **went**
13. The word is **west**
14. The word is **map**
15. The word is **cop**
16. The word is **sap**
17. The word is **pay**
18. The word is **heath**
19. The word is **tame**
20. The word is **pale**
21. The word is **gust**
22. The word is **pus**
23. The word is **then**
24. The word is **cuss**
25. The word is **cook**
26. The word is **lace**
27. The word is **bed**
28. The word is **save**
29. The word is **pit**
30. The word is **reel**
31. The word is **boil**
32. The word is **fib**
33. The word is **bark**
34. The word is **bad**
35. The word is **fill**
36. The word is **gale**
37. The word is **duck**
38. The word is **sit**
39. The word is **tap**
40. The word is **cane**
41. The word is **sang**
42. The word is **saw**
43. The word is **race**
44. The word is **dim**
45. The word is **teak**
46. The word is **din**
47. The word is **seethe**
48. The word is **run**
49. The word is **meat**
50. The word is **dip**

List 3A

1. The word is **bug**
2. The word is **kin**
3. The word is **peach**
4. The word is **rig**
5. The word is **gold**
6. The word is **tick**
7. The word is **pan**
8. The word is **beak**
9. The word is **got**
10. The word is **hit**
11. The word is **sup**
12. The word is **rent**
13. The word is **best**
14. The word is **math**
15. The word is **hop**
16. The word is **sad**
17. The word is **gay**
18. The word is **heap**
19. The word is **fame**
20. The word is **page**
21. The word is **bust**
22. The word is **puff**
23. The word is **ten**
24. The word is **cup**
25. The word is **took**
26. The word is **lake**
27. The word is **shed**
28. The word is **safe**
29. The word is **pig**
30. The word is **eel**
31. The word is **toil**
32. The word is **fill**
33. The word is **lark**
34. The word is **bath**
35. The word is **will**
36. The word is **tale**
37. The word is **dung**
38. The word is **sill**
39. The word is **tang**
40. The word is **cave**
41. The word is **rang**
42. The word is **jaw**
43. The word is **raze**
44. The word is **dip**
45. The word is **teal**
46. The word is **pin**
47. The word is **seem**
48. The word is **fun**
49. The word is **heat**
50. The word is **rip**

UNCONTROLLED COPY FOR INSPECTION AND USE

UNCONTROLLED COPY FOR INSPECTION AND USE

List 4A

1. The word is **bun**
2. The word is **kill**
3. The word is **peace**
4. The word is **big**
5. The word is **told**
6. The word is **kick**
7. The word is **pad**
8. The word is **bean**
9. The word is **lot**
10. The word is **kit**
11. The word is **sum**
12. The word is **bent**
13. The word is **vest**
14. The word is **man**
15. The word is **mop**
16. The word is **sat**
17. The word is **say**
18. The word is **hear**
19. The word is **game**
20. The word is **pave**
21. The word is **dust**
22. The word is **puck**
23. The word is **men**
24. The word is **cuff**
25. The word is **hook**
26. The word is **lane**
27. The word is **fed**
28. The word is **sale**
29. The word is **pip**
30. The word is **peel**
31. The word is **coil**
32. The word is **fit**
33. The word is **mark**
34. The word is **bat**
35. The word is **bill**
36. The word is **bale**
37. The word is **dub**
38. The word is **sing**
39. The word is **tam**
40. The word is **cape**
41. The word is **hang**
42. The word is **law**
43. The word is **rake**
44. The word is **dill**
45. The word is **tease**
46. The word is **fin**
47. The word is **seen**
48. The word is **gun**
49. The word is **seat**
50. The word is **hip**

List 5A

1. The word is **buck**
2. The word is **king**
3. The word is **peat**
4. The word is **dig**
5. The word is **fold**
6. The word is **wick**
7. The word is **pack**
8. The word is **bead**
9. The word is **not**
10. The word is **wit**
11. The word is **sun**
12. The word is **tent**
13. The word is **test**
14. The word is **mad**
15. The word is **pop**
16. The word is **sass**
17. The word is **way**
18. The word is **heat**
19. The word is **same**
20. The word is **pane**
21. The word is **just**
22. The word is **pup**
23. The word is **pen**
24. The word is **cut**
25. The word is **shook**
26. The word is **lay**
27. The word is **red**
28. The word is **sane**
29. The word is **pill**
30. The word is **keel**
31. The word is **oil**
32. The word is **fig**
33. The word is **hark**
34. The word is **ban**
35. The word is **hill**
36. The word is **pale**
37. The word is **dun**
38. The word is **sick**
39. The word is **tab**
40. The word is **cake**
41. The word is **fang**
42. The word is **raw**
43. The word is **rate**
44. The word is **did**
45. The word is **teach**
46. The word is **sin**
47. The word is **seed**
48. The word is **sun**
49. The word is **neat**
50. The word is **tip**

UNCONTROLLED COPY FOR INSPECTION AND USE

UNCONTROLLED COPY FOR INSPECTION AND USE

List 6A

1. The word is **buff**
2. The word is **kid**
3. The word is **peal**
4. The word is **wig**
5. The word is **sold**
6. The word is **pick**
7. The word is **path**
8. The word is **beam**
9. The word is **tot**
10. The word is **sit**
11. The word is **sud**
12. The word is **dent**
13. The word is **nest**
14. The word is **mass**
15. The word is **shop**
16. The word is **sag**
17. The word is **may**
18. The word is **heave**
19. The word is **came**
20. The word is **pace**
21. The word is **rust**
22. The word is **pub**
23. The word is **den**
24. The word is **cub**
25. The word is **look**
26. The word is **lame**
27. The word is **wed**
28. The word is **sake**
29. The word is **pick**
30. The word is **heel**
31. The word is **foil**
32. The word is **fin**
33. The word is **dark**
34. The word is **back**
35. The word is **kill**
36. The word is **sale**
37. The word is **dug**
38. The word is **sip**
39. The word is **tan**
40. The word is **came**
41. The word is **gang**
42. The word is **paw**
43. The word is **rave**
44. The word is **din**
45. The word is **tear**
46. The word is **tin**
47. The word is **seep**
48. The word is **nun**
49. The word is **beat**
50. The word is **lip**

List 7A

1. The word is **lick**
2. The word is **beat**
3. The word is **puff**
4. The word is **cook**
5. The word is **tip**
6. The word is **rave**
7. The word is **hang**
8. The word is **till**
9. The word is **math**
10. The word is **sale**
11. The word is **same**
12. The word is **peal**
13. The word is **kit**
14. The word is **sat**
15. The word is **sin**
16. The word is **gold**
17. The word is **buff**
18. The word is **lay**
19. The word is **nun**
20. The word is **must**
21. The word is **pad**
22. The word is **din**
23. The word is **sit**
24. The word is **win**
25. The word is **teak**
26. The word is **dent**
27. The word is **sub**
28. The word is **led**
29. The word is **tot**
30. The word is **dub**
31. The word is **pip**
32. The word is **seen**
33. The word is **way**
34. The word is **west**
35. The word is **pace**
36. The word is **bat**
37. The word is **mop**
38. The word is **big**
39. The word is **tab**
40. The word is **case**
41. The word is **name**
42. The word is **soil**
43. The word is **fin**
44. The word is **cuff**
45. The word is **heal**
46. The word is **hark**
47. The word is **heat**
48. The word is **then**
49. The word is **law**
50. The word is **bean**

UNCONTROLLED COPY FOR INSPECTION AND USE

UNCONTROLLED COPY FOR INSPECTION AND USE

List 8A

1. The word is **wick**
2. The word is **neat**
3. The word is **puck**
4. The word is **took**
5. The word is **rip**
6. The word is **ray**
7. The word is **sang**
8. The word is **will**
9. The word is **man**
10. The word is **gale**
11. The word is **safe**
12. The word is **peas**
13. The word is **kid**
14. The word is **sass**
15. The word is **sick**
16. The word is **hold**
17. The word is **but**
18. The word is **lane**
19. The word is **bun**
20. The word is **just**
21. The word is **pan**
22. The word is **dig**
23. The word is **bit**
24. The word is **pin**
25. The word is **tease**
26. The word is **sent**
27. The word is **sup**
28. The word is **red**
29. The word is **not**
30. The word is **dung**
31. The word is **pig**
32. The word is **seem**
33. The word is **day**
34. The word is **rest**
35. The word is **page**
36. The word is **bash**
37. The word is **shop**
38. The word is **fig**
39. The word is **tam**
40. The word is **cane**
41. The word is **same**
42. The word is **toil**
43. The word is **fill**
44. The word is **cuss**
45. The word is **feel**
46. The word is **bark**
47. The word is **heath**
48. The word is **ten**
49. The word is **thaw**
50. The word is **bead**

List 9A

1. The word is **pick**
2. The word is **meat**
3. The word is **pub**
4. The word is **look**
5. The word is **sip**
6. The word is **rake**
7. The word is **fang**
8. The word is **bill**
9. The word is **mat**
10. The word is **male**
11. The word is **sane**
12. The word is **peach**
13. The word is **kill**
14. The word is **sack**
15. The word is **sill**
16. The word is **cold**
17. The word is **bug**
18. The word is **lace**
19. The word is **sun**
20. The word is **gust**
21. The word is **pass**
22. The word is **dim**
23. The word is **fit**
24. The word is **fin**
25. The word is **teach**
26. The word is **went**
27. The word is **sum**
28. The word is **wed**
29. The word is **hot**
30. The word is **dud**
31. The word is **pill**
32. The word is **seethe**
33. The word is **pay**
34. The word is **vest**
35. The word is **pace**
36. The word is **back**
37. The word is **top**
38. The word is **rig**
39. The word is **tap**
40. The word is **cave**
41. The word is **game**
42. The word is **foil**
43. The word is **fit**
44. The word is **cup**
45. The word is **eel**
46. The word is **park**
47. The word is **heap**
48. The word is **pen**
49. The word is **paw**
50. The word is **beam**

UNCONTROLLED COPY FOR INSPECTION AND USE

List 10A

1. The word is **kick**
2. The word is **feat**
3. The word is **pup**
4. The word is **book**
5. The word is **lip**
6. The word is **rate**
7. The word is **bang**
8. The word is **fill**
9. The word is **mass**
10. The word is **tale**
11. The word is **sale**
12. The word is **peak**
13. The word is **king**
14. The word is **sag**
15. The word is **sip**
16. The word is **told**
17. The word is **bun**
18. The word is **lake**
19. The word is **gun**
20. The word is **bust**
21. The word is **pat**
22. The word is **did**
23. The word is **kit**
24. The word is **tin**
25. The word is **tear**
26. The word is **bent**
27. The word is **sun**
28. The word is **shed**
29. The word is **pot**
30. The word is **duck**
31. The word is **pin**
32. The word is **seed**
33. The word is **gay**
34. The word is **test**
35. The word is **pave**
36. The word is **bath**
37. The word is **pop**
38. The word is **pig**
39. The word is **tan**
40. The word is **cape**
41. The word is **came**
42. The word is **boil**
43. The word is **fib**
44. The word is **cud**
45. The word is **keel**
46. The word is **lark**
47. The word is **heave**
48. The word is **den**
49. The word is **saw**
50. The word is **beat**

UNCONTROLLED COPY FOR INSPECTION AND USE

List 11A

1. The word is **tick**
2. The word is **heat**
3. The word is **pus**
4. The word is **hook**
5. The word is **dip**
6. The word is **raze**
7. The word is **gang**
8. The word is **hill**
9. The word is **mad**
10. The word is **bale**
11. The word is **save**
12. The word is **peace**
13. The word is **kin**
14. The word is **sad**
15. The word is **sit**
16. The word is **fold**
17. The word is **bus**
18. The word is **late**
19. The word is **run**
20. The word is **rust**
21. The word is **pack**
22. The word is **dill**
23. The word is **wit**
24. The word is **din**
25. The word is **teal**
26. The word is **rent**
27. The word is **sung**
28. The word is **bed**
29. The word is **lot**
30. The word is **dug**
31. The word is **pick**
32. The word is **seek**
33. The word is **say**
34. The word is **best**
35. The word is **pay**
36. The word is **ban**
37. The word is **hop**
38. The word is **dig**
39. The word is **tang**
40. The word is **cake**
41. The word is **fame**
42. The word is **coil**
43. The word is **fig**
44. The word is **cut**
45. The word is **peel**
46. The word is **mark**
47. The word is **heal**
48. The word is **men**
49. The word is **jaw**
50. The word is **beach**

UNCONTROLLED COPY FOR INSPECTION AND USE
UNCONTROLLED COPY FOR INSPECTION AND USE
UNCONTROLLED COPY FOR INSPECTION AND USE
UNCONTROLLED COPY FOR INSPECTION AND USE

List 12A

1. The word is **sick**
2. The word is **seat**
3. The word is **pun**
4. The word is **shook**
5. The word is **hip**
6. The word is **race**
7. The word is **rang**
8. The word is **kill**
9. The word is **map**
10. The word is **pale**
11. The word is **sake**
12. The word is **peat**
13. The word is **kick**
14. The word is **sap**
15. The word is **sing**
16. The word is **sold**
17. The word is **buck**
18. The word is **lame**
19. The word is **fun**
20. The word is **dust**
21. The word is **path**
22. The word is **dip**
23. The word is **hit**
24. The word is **sin**
25. The word is **team**
26. The word is **tent**
27. The word is **sud**
28. The word is **fed**
29. The word is **got**
30. The word is **dun**
31. The word is **pit**
32. The word is **seep**
33. The word is **may**
34. The word is **nest**
35. The word is **pane**
36. The word is **bad**
37. The word is **cop**
38. The word is **wig**
39. The word is **tack**
40. The word is **came**
41. The word is **tame**
42. The word is **oil**
43. The word is **fizz**
44. The word is **cub**
45. The word is **reel**
46. The word is **dark**
47. The word is **hear**
48. The word is **hen**
49. The word is **raw**
50. The word is **beak**

UNCONTROLLED COPY FOR INSPECTION AND USE
UNCONTROLLED COPY FOR INSPECTION AND USE
UNCONTROLLED COPY FOR INSPECTION AND USE
UNCONTROLLED COPY FOR INSPECTION AND USE

Revision History

Revision	Date	Reason for Revision
0.0	21 March 2020	Original release

UNCONTROLLED COPY
FOR INSPECTION AND USE
UNCONTROLLED COPY
FOR INSPECTION AND USE
UNCONTROLLED COPY
FOR INSPECTION AND USE
UNCONTROLLED COPY
FOR INSPECTION AND USE
UNCONTROLLED COPY
FOR INSPECTION AND USE

DETERMINATION OF AIR FLOW FOR
POWERED AIR-PURIFYING RESPIRATORS
STANDARD TESTING PROCEDURE (STP)

1. PURPOSE

This test establishes the procedure for ensuring that the level of protection provided by the air flow test requirements on powered air-purifying respirator submitted for Approval, Extension of Approval, or examined during Certified Product Audits, meet the minimum certification standards set forth in 42 CFR, Part 84, Subpart G, Section 84.63(a)(c)(d), and Subpart KK, Section 84.1157(a); Volume 60, Number 110, June 8, 1995.

2. GENERAL

This procedure describes the Determination of Air Flow For Powered Air-Purifying Respirators test in sufficient detail that a person in the appropriate technical field can conduct the test and determine whether or not the product passes the test.

3. EQUIPMENT/MATERIALS

3.1. The list of necessary test equipment and materials is as follows:

3.1.1. Air tight chamber - approximately 24 inches by 24 inches by 16 inches with a hinged door, a 3 inch diameter inlet for accepting breathing tubes and adapters, a one inch diameter outlet, and a 1/4 inch outlet for a manometer probe.

3.1.2. Setra electronic manometer.

3.1.3. Tubing and connectors.

3.1.4. Dry test meter - 10 cubic feet per revolution.

3.1.5. Vacuum source - Spencer turbo compressor Model 075-1/3.

3.1.6. Digital stopwatch.

UNCONTROLLED COPY

4. TESTING REQUIREMENTS AND CONDITIONS

4.1. Prior to beginning any testing, all measuring equipment to be used must have been calibrated in accordance with the manufacturer's calibration procedure and schedule. At a minimum, all measuring equipment utilized for this testing must have been calibrated within the preceding 12 months using a method traceable to the National Institute of Standards and Technology (NIST).

4.2. Normal laboratory safety practices must be observed. This includes safety precautions described in the current ALOSH Facility Laboratory Safety Manual.

4.2.1. Safety glasses, lab coats, and hard-toe shoes must be worn at all times.

4.2.2. Work benches must be maintained free of clutter and non-essential test equipment.

4.2.3. When handling any glass laboratory equipment, lab technicians and personnel must wear special gloves which protect against lacerations or punctures.

5. PROCEDURE

Note: Reference Section 3 for equipment, model numbers and manufacturers. For calibration purposes use those described in the manufacturer's operation and maintenance manuals.

5.1. Set up the equipment as show in Figure 1.

5.2. Connect the respirator to the chamber. On units with breathing tubes, the blower is placed outside of the chamber and the breathing tube is attached to an adapter identical to the manufacturer's connector on the facepiece, helmet, or hood. On units where the blower is in the helmet, an adapter is attached to the helmet and inserted through the inlet of the chamber and sealed.

5.3. Close the door of the chamber.

5.4. Check the electric manometer for zero, adjust to zero if necessary.

5.5. Turn on the PAPR and the vacuum pump.

5.6. Attach the tubing to the electric manometer and adjust to zero using the valve on the vacuum pump.

5.7. Check all connections for leaks.

5.8. If a leak is detected, reseal and readjust the vacuum.

5.9. Time 1 minute on a stopwatch and count the number of CFM on the dry test meter. 1 revolution equals 10 CFM.

UNCONTROLLED COPY

5.10. Calculate the airflow of the respirator using equation #1 (Listed in 6.3).

5.11. Disconnect manometer tubing.

5.12. Turn off the PAPR and vacuum pump.

6. PASS/FAIL CRITERIA

6.1. The criterion for passing this test is set forth in 42 CFR, Part 84, Subpart G, Section 84.63(a)(c)(d), and Subpart KK, Section 84.1157(a); Volume 60, Number 110, June 8, 1995.

6.2. This test establishes the standard procedure for ensuring that:

84.63. Test requirements; general.

(a) Each respirator and respirator component shall when tested by the applicant and by the Institute, meet the applicable requirements set forth in subparts H through L of this part.

(c) In addition to the minimum requirements set forth in subparts H through L of this part, the Institute reserves the right to require, as a further condition of approval, any additional requirements deemed necessary to establish the quality, effectiveness, and safety of any respirator used as protection against hazardous atmospheres.

(d) Where it is determined after receipt of an application that additional requirements will be required for approval, the Institute will notify the applicant in writing of these additional requirements, and necessary examinations, inspections, or tests, stating generally the reasons for such requirements, examinations, inspections, or tests.

84.1157. Chemical cartridge respirators with particulate filters; performance requirements; general. Chemical cartridge respirators with particulate filters and the individual components of each such device shall, as appropriate, meet the following minimum requirements for performance and protection:

(a) Breathing resistance test. (1) Resistance to airflow will be measured in the facepiece, mouthpiece, hood, or helmet of a chemical cartridge respirator mounted on a test fixture with air flowing at a continuous rate of 85 liters per minute, both before and after each test conducted in accordance with paragraphs (d) through (f) of this section

UNCONTROLLED COPY

(2) The maximum allowable resistance requirements for chemical cartridge respirators are as follows:

Maximum Resistance
[mm. water-column height]

Type of chemical cartridge respirator	Inhalation		Exhalation
		Final[1]	
For gases, vapors, or gases and vapors, and dusts, fumes, and mists	50	70	20
For gases, vapors, or gases and vapors, and mists of paints, lacquers, and enamels	50	70	20

[1]Measured at end of service life specified in Table 11 in subpart L of this part.

6.3. Equation #1: Airflow (LPM) = $\frac{(28.32 \text{ LPM}) * (\text{number of CFM})}{1 \text{ min}}$

7. RECORDS/TEST SHEETS

7.1. Test data collected shall be recorded on the DETERMINATION OF AIR FLOW FOR POWERED AIR PURIFYING RESPIRATORS test data sheet.

7.2. All videotapes and photographs of the actual test being performed, or of the tested equipment shall be maintained in the task file as part of the permanent record.

7.3. All equipment failing any portion of this test will be handled as follows:

7.3.1. If the failure occurs on a new certification application, or extension of approval application, send a test report to the RCT Leader and prepare the hardware for return to the manufacturer.

7.3.2. If the failure occurs on hardware examined under an Off-the-Shelf Audit the hardware will be examined by a technician and the RCT Leader for cause. All equipment failing any portion of this test may be sent to the manufacturer for examination and then returned to NIOSH. However, the hardware tested shall be held at the testing laboratory until authorized for release by the RCT Leader, or his designee, following the standard operating procedures outlined in Procedure for Scheduling, and Processing Post-Certification Product Audits, RB-SOP-0005-00.

UNCONTROLLED COPY

UNCONTROLLED COPY

National Institute for Occupational Safety and Health
Respirator Branch
Test Data Sheet

Task Number:

Test:

Manufacturer:

Item Tested:

Reference No.:

STP No.:

Mask Type:

AIR FLOW			
Sample	Minimum Allowed (Lpm)	Actual (Lpm)	Result
Initial			
Final			
Overall Result:			

Signature:

Engineering Technician

Date: ____________

UNCONTROLLED COPY

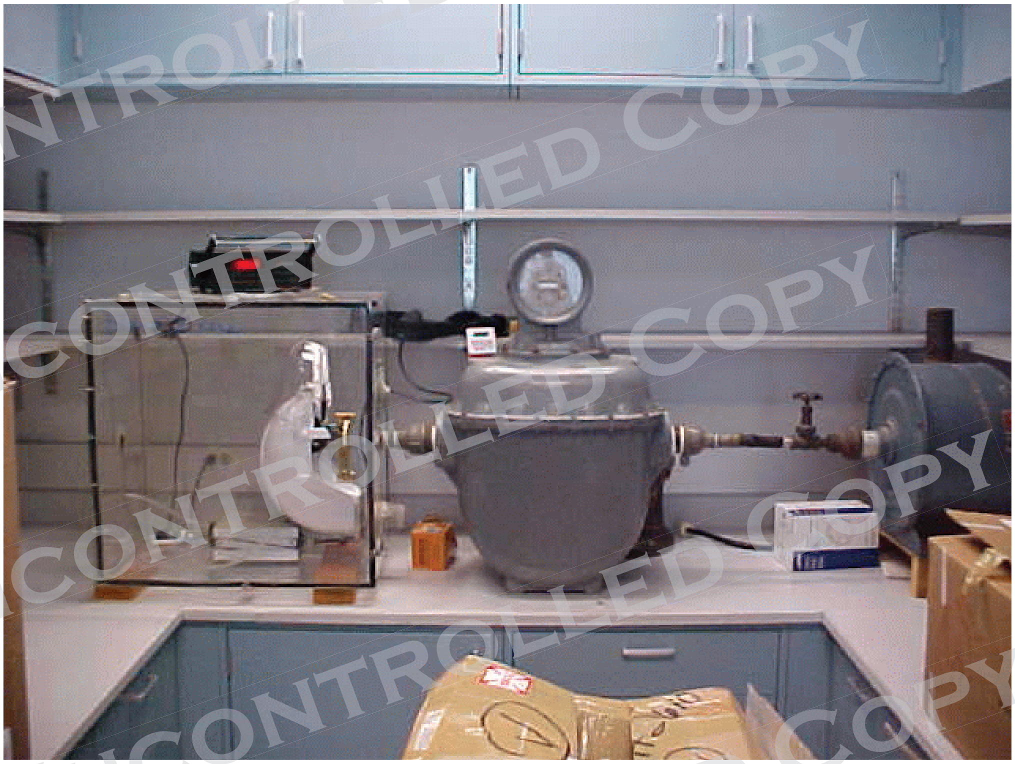

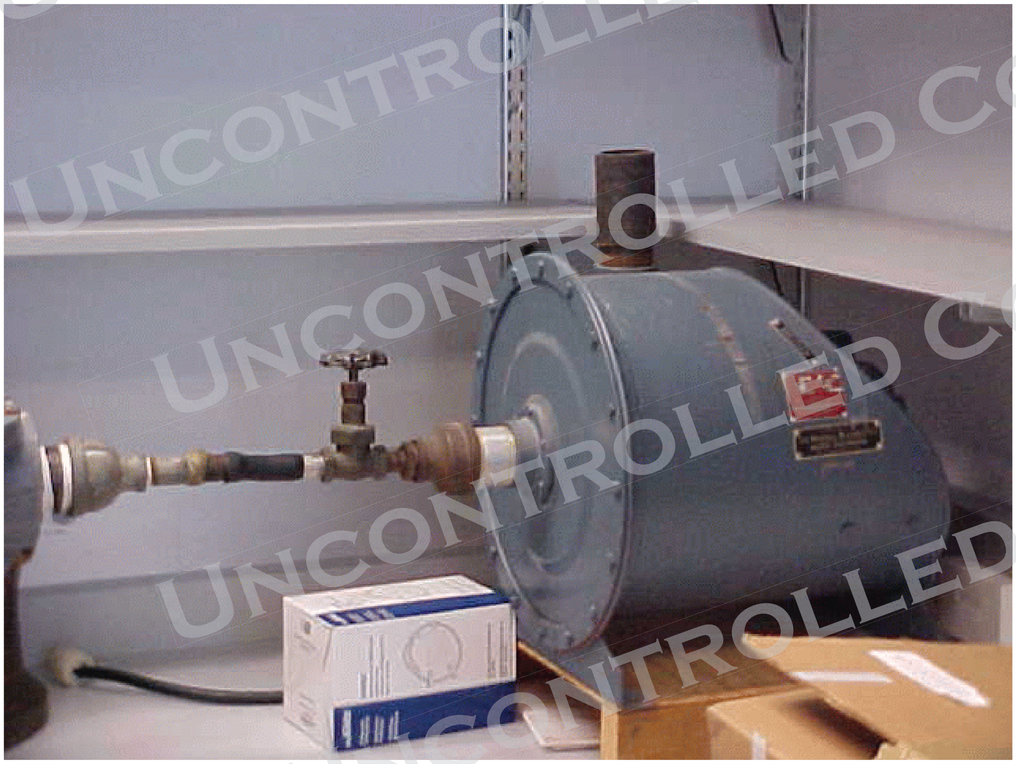

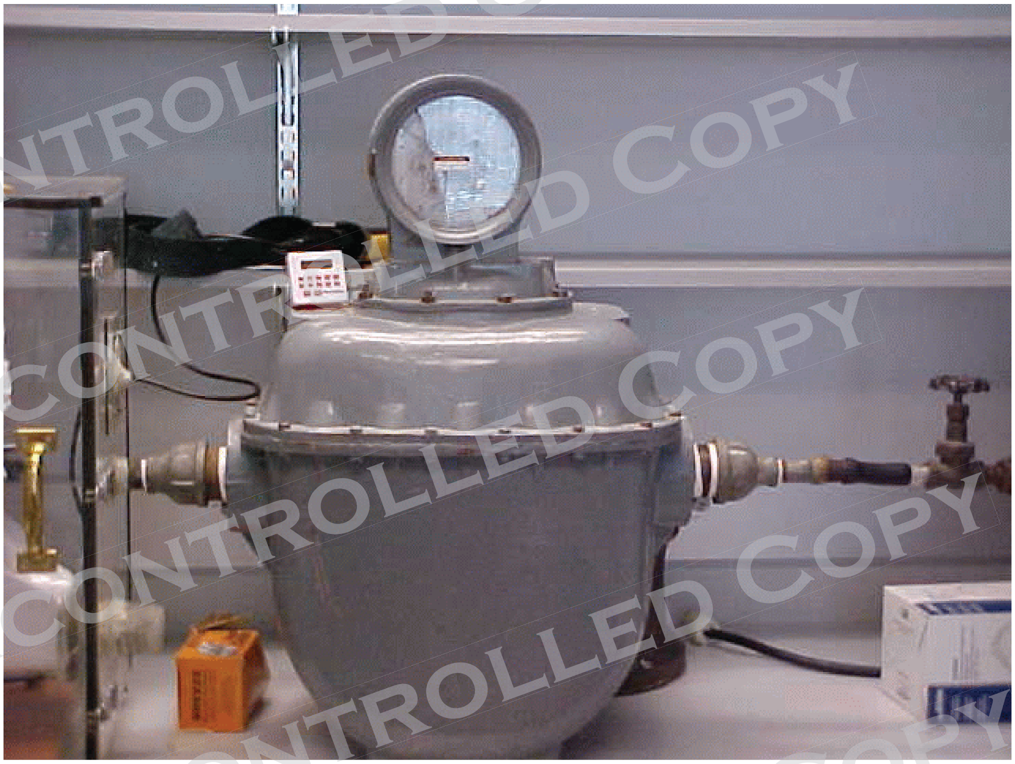

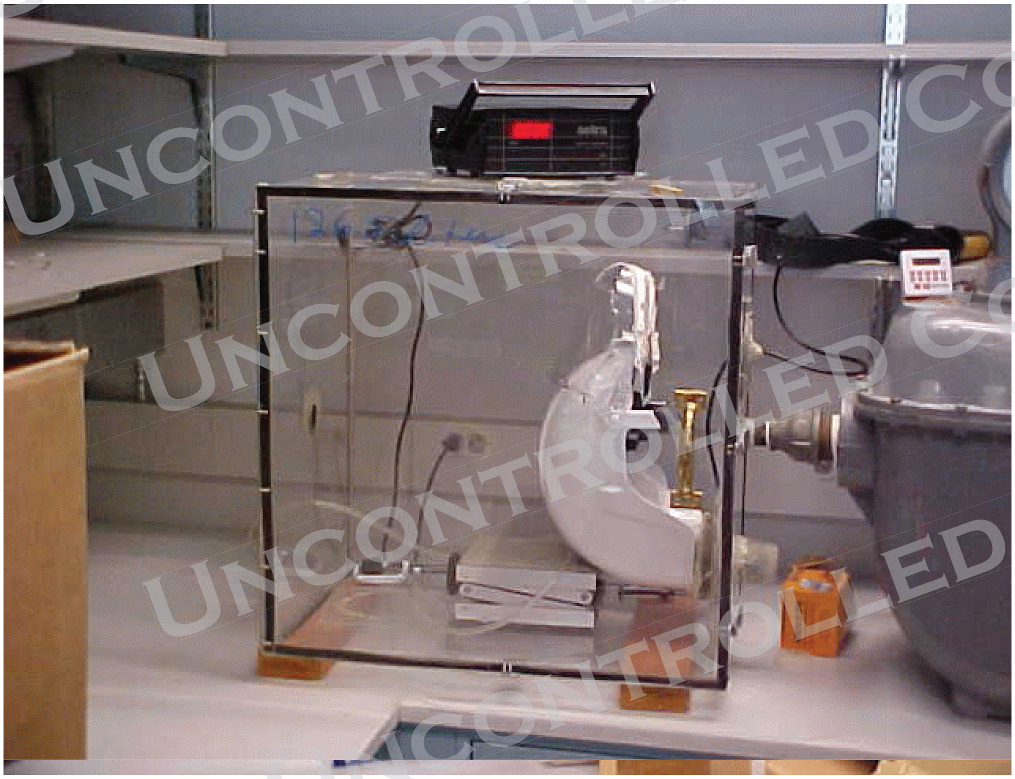

UNCONTROLLED COPY

Revision History

Revision	Date	Reason for Revision
1.0	12 July 2001	Historic document
1.1	6 June 2005	Update header and format to reflect lab move from Morgantown, WV<br>No changes to method

UNCONTROLLED COPY

UNCONTROLLED COPY

DETERMINATION OF NOISE LEVEL TEST,
POWERED AIR-PURIFYING RESPIRATOR WITH HOODS OR HELMETS
STANDARD TESTING PROCEDURE (STP)

1. PURPOSE

This document establishes the procedure for ensuring that operational noise levels of powered air-purifying respirators with hoods or helmets, as submitted for Approval, Extension of Approval, or examined during Certified Product Audits, do not exceed the established maximum certification requirements as provided for by 42 CFR Part 84, Subpart G, Section 84.63(a)(c)(d), Subpart L, Section 84.202, and Subpart KK, Section 84.1139; Volume 60, Number 110, June 8, 1995.

2. GENERAL

This STP describes the Determination of Noise Level Test, Powered Air-Purifying Respirators with Hoods or Helmets test in sufficient detail that a person knowledgeable in the appropriate technical field can select equipment with the necessary resolution, conduct the test, and determine whether or not the product passes the test.

3. EQUIPMENT/MATERIALS

3.1. The list of necessary test equipment and materials follows.

3.1.1. Completely assembled, powered air-purifying hood or helmet respirator in the configuration as worn by the user with fully charged battery and new air purifying elements

3.1.2. Life-size mannequin

3.1.3. Precision, fast-response sound level meter with built in A-weighting network, capable of averaging measurements over selected time intervals of at least up to 30 seconds. Sound level meter must accommodate two microphone inputs.

4. TESTING REQUIREMENTS AND CONDITIONS

4.1. Prior to beginning any testing, confirm that all measuring equipment employed has been calibrated in accordance with the testing laboratory's calibration procedure and schedule. All measuring equipment utilized for this testing must have been calibrated using a method traceable to recognized international standards when available.

4.2. A background noise level of no greater than 60 dB shall be established and maintained in the location where the procedure is performed.

5. PROCEDURE

5.1 Prior to the use of the test subjects, a noise level screening test will be performed on the complete respirator assembly affixed to a mannequin. The purpose of the screening is to prevent exposing subjects to noise levels which may exceed 85 dBA.

5.1.1. Position the microphones of the sound level meter on each ear of the mannequin.

5.1.2. Using the sound level meter, verify the background noise requirement per section 4.2.

5.1.1. Following the respirator manufacturer's instructions, mount the respirator assembly onto the mannequin.

5.2. Each sample measurement should be averaged over 30 seconds. Once the dBA noise level of the mannequin setup has been determined to be below the 85 dBA safety limit, test subject testing may begin.

5.3 The evaluation is made on three test subjects.

5.4. It is recommended that both males and females be employed as test subjects, and that a wide variation in body size and shape of subjects be sought.

5.5. The test subjects will be allowed to wear ear-insert type hearing protectors, which do not interfere with the positioning of the microphones, if they desire. A choice of protectors will be provided for this purpose.

5.6. Two readings are taken on each subject at both ears and the results averaged.

5.7. Record the results.

6. PASS/FAIL CRITERIA

6.1. The criterion for passing this test is set forth in 42 CFR Part 84, Subpart G, Section 84.63(a)(c)(d), Subpart L, Section 84.202 and Subpart KK, Section 84.1139; Volume 60, Number 110, June 8, 1995.

6.2. This test establishes the standard procedure for ensuring that:

84.63 Test requirements; general.

(a) Each respirator and respirator component shall when tested by the applicant and by the Institute, meet the applicable requirements set forth in subparts H through L of this part.

(c) In addition to the minimum requirements set forth in subparts H through L of this part, the Institute reserves the right to require, as a further condition of approval, any

UNCONTROLLED COPY

additional requirements deemed necessary to establish the quality, effectiveness, and safety of any respirator used as protection against hazardous atmospheres.

(d) Where it is determined after receipt of an application that additional requirements will be required for approval, the Institute will notify the applicant in writing of these additional requirements, and necessary examinations, inspections, or tests, stating generally the reasons for such requirements, examinations, inspections, or tests.

84.202 Air velocity and noise levels; hoods and helmets; minimum requirements.

Noise levels generated by the respirator will be measured inside the hood or helmet at maximum airflow obtainable and shall not exceed 80 dBA.

84.1139 Air velocity and noise levels; hoods and helmets; minimum requirements.

Noise levels generated by the respirator will be measured inside the hood or helmet at maximum airflow obtainable and shall not exceed 80 dBA.

7. RECORDS\TEST SHEETS

7.1. Record test data in a format that shall be stored and retrievable. Data is to be reported as shown in the attached example data sheet.

8. ATTACHMENTS

8.1. Example Test Data Sheet

UNCONTROLLED COPY

Attachment 8.1. Example Test Data Sheet

National Institute for Occupational Safety and Health
Respirator Branch
Test Data Sheet

NIOSH

Task Number:
Test: Sound Level Test-Direct dB
Manufacturer:
Item Tested:

Reference No.: CFR 84.1139; 84.202
STP No.: 30.1

Subject	Trial 1 (dba)		Trial 2 (dba)		Average (dba)		Maximum Allowable (dba)	Result
	Left Ear	Right Ear	Left Ear	Right Ear	Left Ear	Right Ear		
Manikin							85	
1							80	
2							80	
3							80	

Overall Result:

Signature Date:_____

Engineering Technician

Task Number:
Test: Sound Level Test-Direct dB
Manufacturer: Item Tested:

Reference No.: CFR 84.1139; 84.202
STP No.: 30.1

Comments:
Testing was done using the (respirator part numbers). All test subjects were medically cleared for testing.
Was all equipment verified to be in calibration throughout all testing? O Yes O No

Signature Date:_____

Engineering Technician

UNCONTROLLED COPY

Revision History

Revision	Date	Reason for Revision
1.0	7 March 2002	Historic document
1.1	14 June 2005	Update header and format to reflect lab move from Morgantown, WV No changes to method
2.0	28 May 2019	The document is updated to current style and content standards. There is no change to the test set up or method, but the specified sound measurement instrument has been updated. The ability to collect an average measurement expressed in dBA over the specified 30-second interval eliminates the need to convert dose to dBA.

UNCONTROLLED COPY

NIOSH — National Institute for Occupational Safety and Health	National Institute for Occupational Safety and Health<br>National Personal Protective Technology Laboratory<br>P.O. Box 18070<br>Pittsburgh, PA 15236

Procedure No. TEB-APR-STP-0001	Revision: 2.0	Date: 14 April 2009

DETERMINATION OF PARTICULATE FILTER PENETRATION TEST
POWERED AIR-PURIFYING RESPIRATOR FILTERS
STANDARD TESTING PROCEDURE (STP)

1. PURPOSE

This test establishes the procedure for ensuring that the level of protection provided by powered air-purifying respirator filters submitted for Approval, Extension of Approval, or examined during Certified Product Audits, meet the filter penetration requirements set forth in 42 CFR, Part 84, Subpart G, Section 84.63(a)(c)(d) and Subpart KK, Section 84.1151(a)(c), except that the flow requirements of Section 84.1151(a)(c) are not used. In their place, flow requirements specified in Section 84.1156(c)(2) are applied. This is done in order that PAPR filters are tested, to the extent possible, at the minimum required air flow rate of the PAPR.

2. GENERAL

This STP describes the Determination of Particulate Filter Penetration Test, Powered Air-purifying Respirator Filters test in sufficient detail that a person knowledgeable in the appropriate technical field can select equipment with the necessary resolution, conduct the test, and determine whether or not the product passes the test.

3. EQUIPMENT/MATERIALS

3.1. The list of necessary test equipment and materials follows:

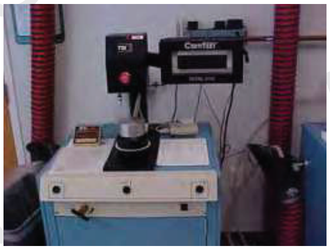

3.1.1. TSI Model 8130 Automated Filter Tester or equivalent instrument. Air flow control accuracy is 2% of full scale. Pressure measurement accuracy is 2% of full scale. Penetrations can be measured to 0.001%, efficiencies to 99.999%.

Approvals: First Level	Second Level	Third Level	Fourth Level

UNCONTROLLED COPY

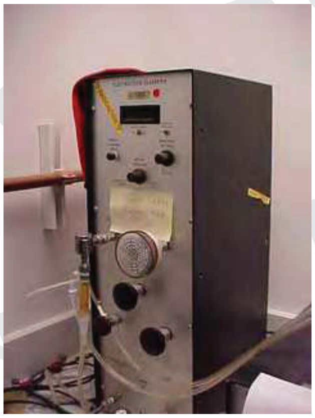

3.1.2. Particle sizing instrument (such as TSI Model 3936 Scanning Mobility Particle Size Spectrometer or equivalent) that is capable of determining submicrometer particles according to count median diameter (CMD).

.

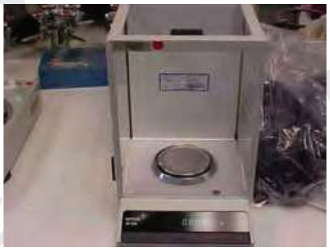

3.1.3. Microbalance accurate to 0.0001 grams (g).

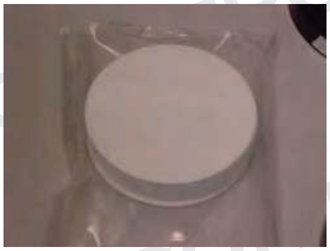

3.1.4. Gelman 102 mm diameter, type A/E glass filters or equivalent high efficiency filters with a 1 micrometer pore size.

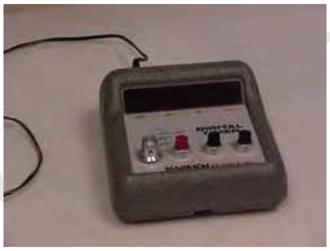

3.1.5. Timer (accurate to 0.01 second).

UNCONTROLLED COPY

3.1.6. Dioctyl phthalate ((DOP, di(2-ethylhexyl)phthalate)) min. 98%.

3.1.7. Respirator filter holder supplied for specific manufacturer type which is compatible with TSI filter tester. NIOSH will not be obligated to use these holders for actual certification testing. All manufacturer test fixtures must be correlated with the NIOSH test method (see Work Instruction WI- 1611).

3.1.8 Thermal printer (supplied) or optional data acquisition system.

3.2. Refer to the following Work Instructions for further information on performing this test:
TEB-RCT-APR-WI-1011 – Laboratory Safety Procedures for Particulate Tests for Powered Air Purifying Respirators
TEB-RCT-APR-WI-1111 – Calibration Procedures for Particulate Test for Powered Respirators
TEB-RCT-APR-WI-1211 – Start–Up and Shut–Down Procedures for Particulate Test for Powered Air Purifying Respirators
TEB-RCT-APR-WI-1411 – Reporting Results for Particulate Test for Powered Air Purifying Respirators
TEB-RCT-APR-WI-1511 – Checking System Performance for Particulate Test for Powered Air Purifying Respirators
TEB-RCT-APR-WI-1611 – Correlating Manufacturer – Supplied Test Fixtures for Particulate Test for Powered Air Purifying Respirators

4. TESTING REQUIREMENTS AND CONDITIONS

4.1. Prior to beginning any testing, all measuring equipment to be used must have been calibrated in accordance with the testing laboratory's calibration procedure and schedule. All measuring equipment utilized for this testing must have been calibrated using a method traceable to the National Institute of Standards and Technology (NIST) when available.

4.2. Any laboratory using this procedure to supply certification test data as a contractor to NIOSH will be subject to the provisions of the NIOSH Supplier Qualification Program (SQP). This program is based on the tenets of *ISO/IEC 17025, the NIOSH Manual of Analytical Methods* and other NIOSH guidelines. An initial complete quality system audit and follow on audits are requirements of the program. Additional details of the Program and its requirements can be obtained directly from the Institute.*
***Note** 4.2 does not apply to Pretest data from applicants as required under 42 CFR 84.64.

4.3. Precision and accuracy (P&A) must be determined for each instrument in accordance with laboratory procedures and NIOSH/NPPTL guidance. Sound practice requires, under *NIOSH Manual of Analytical Methods*, demonstrating a tolerance range of expected data performance of a plus or minus 25% of a 95% confidence interval of the stated standard requirement. NIOSH/NPPTL P&A tolerance can be higher but not lower.

UNCONTROLLED COPY

4.4. The precision and accuracy of this method is monitored by the validation method which is incorporated in the automated filter tester procedure. This procedure is performed on a daily basis when testing is performed. This procedure is designed to test many aspects of the method, for proper photometer and general system operation. The validation technique uses "green line" filter media discs, 6 inch diameter, HE 1071 grade, H & V brand, P/N 813010, with a known penetration range, which are tested at least once in each 8-hour test period (see 5.2.5).

4.4.1. Two sheets of unused filter media are stacked together and the penetration, flow rate and pressure drop are measured to evaluate the higher range of penetration values. Five unused sheets are stacked together to evaluate the lower range of penetration values.

4.4.2. The analysis of these readings over the long term was used to examine the precision and accuracy of this test method. The table below summarizes the data.

	Two Sheets	Five Sheets
Mean	2.459%	0.011%
Std. Dev.	0.157	0.001
Range	2.04 – 2.97%	0.008 – 0.017%
N	56	56

4.5. Normal laboratory safety practices must be observed. Please refer to Material Safety Data Sheets and the current NIOSH Pittsburgh Health and Safety Program for the proper protection and care in handling, storing, and disposing of the chemicals used in this procedure.

4.6. Dioctyl phthalate is considered a low hazard material with a recommended exposure limit (REL) of 5 mg/m^3 with a short-term exposure limit of 10 mg/m^3. It may cause mild skin or eye irritation. Carcinogenic effects: Classified as a proven animal carcinogen with unknown relevance to humans by ACGIH; classified as a suspect carcinogen by NTP; not listed by IARC. Local exhaust ventilation is used for the potential sources of DOP from the TSI 8310 filter tester. Safety eyewear and a lab coat should be worn. Splash goggles, protective clothing, boots and gloves should be worn in case of a large spill. Discharge, treatment or disposal may be subject to national, state or local laws.

5. PROCEDURE

Note: Reference Section 3. for equipment, model numbers and manufacturers. For calibration purposes use those described in the manufacturers' operation and maintenance manuals.

5.1. Respirator filters will be challenged by a neat cold–nebulized DOP aerosol at 25 ±5˚C that has been neutralized to the Boltzmann equilibrium state. The particle size distribution will be a count median diameter of 0.185 ± 0.020 micrometer and a geometric standard deviation not exceeding 1.6. Each respirator filter unit will be challenged with an aerosol concentration of 100 ± 10 mg/m^3.

UNCONTROLLED COPY

5.1.1. The DOP aerosol concentration will be determined daily by the following gravimetric method and calculated as milligrams per cubic meter (mg/m^3).

5.1.2. Weigh a Gelman 102 mm filter to the nearest 0.1 mg., mount in the gravimetric filter holder, subject it to the generated aerosol at 30 Lpm for 40 minutes , and reweigh the filter. Use a timer to monitor the duration of the test. Record the pre- and post-weights, time, and average flow rate on the data sheet and calculate the aerosol concentration in mg/m^3 by the following formula:

$$\text{Concentration in } mg/m^3 = \frac{W2 - W1}{(Q / 1000)\ (T)}$$

Where:
W1 = Initial filter weight in mgs.
W2 = Final filter weight in mgs.
Q = Flowrate in liters per minute
T = Elapsed time in minutes

With a flowrate of 30 Lpm for 40 minutes, the above formula simplifies to:

$$C = \frac{W2 - W1}{1.2}$$

5.1.3. The upstream and downstream photometer readings are used for monitoring stability and for calculating a photometer correlation factor (CF). The correlation factor is determined with an empty filter holder and is calculated internally as shown below:

$$CF = \frac{\text{Downstream Photometer Voltage} - \text{Downstream Background Voltage}}{\text{Upstream Photometer Voltage} - \text{Downstream Background Voltage}}$$

The correlation factor is used by the software to express the upstream photometer signal in terms of the downstream photometer signal. Follow Work Instruction WI- 1511 for determining, monitoring and recording the CF.

5.1.4. The DOP particle size distribution shall be verified using “green line” filter discs supplied by TSI with a known penetration range. Graphs of penetration vs. resistance for two sheets and five sheets of stacked filter discs are supplied with each lot of the standard filters, with a central line and upper and lower lines representing the expected penetration range at a given resistance. The test data should fall within an acceptance zone having boundaries defined by the upper and lower curves on the graphs. Follow the procedure in Work Instruction WI-1505. The standard filter test using both 2 sheets and 5 sheets will be run at least once in each 8 hour test period to verify that the aerosol distribution is within the acceptance zone.

5.1.5. If the instantaneous filter penetration is not within the acceptance zone for any sample, abort testing and check the aerosol particle size with the Scanning

UNCONTROLLED COPY

Mobility Particle Size (SMPS) Spectrometer.

5.2. The DOP particle size will be monitored at least once every three months (quarterly) with the SMPS spectrometer to ensure the particle size distribution count median diameter remains in the range of 0.185 ± 0.020 micrometer with a geometric standard deviation of not more than 1.6.

5.3. Filters shall be tested as follows:

5.3.1. The filter, including the filter holders and gaskets, shall be tested for particle penetration. When the filtering element is not separable from the cartridge or canister, the complete component shall be tested.

5.3.2. When filters are not separable from the respirator body, any exhalation valves shall be sealed to ensure that any leakage due to an exhalation valve is not included in the filter penetration measurement.

5.3.3. Filters not separable from cartridges, canisters, respirators, and odd or unusually shaped filters may be tested on a headform assembly or an assembly provided by manufacturer. Note: NIOSH is not obligated to use the headform assembly or any assembly provided by the manufacturer for certification testing.

5.4. Filters shall be mounted and sealed on holders to prevent leakage around the filter holder. PAPRs are normally designed to use from one to four filters. Filters shall be tested using a single filter regardless of the number of filters used on the unit. Adjustment is made to the flow rate of the test by dividing the specified flow rate for the test, which is based on the minimum required air flow of 115 lpm for tight fitting PAPRs and 170 lpm for loose fitting PAPRs, by the number of filters used. The table below shows the test flow rate depending on the type of PAPR and the number of filters employed. The highest obtainable air flow for the automated filter testers is typically 96 lpm and this flow rate will be used to test single PAPR filters.

	PAPR FILTER TEST - FLOW RATE (LPM)	
NUMBER OF FILTERS	TIGHT FITTING FACEPIECE	LOOSE FITTING FACEPIECE
1	96 ± 5	96 ± 5
2	57 ± 3	85 ± 4
3	38 ± 2	57 ± 3
4	29 ± 2	43 ± 2

5.4.1. The challenge flow rate must be checked for stability for at least 30 seconds prior to testing.

5.4.2. If using a TSI 8130 tester, the tester rise time shall be set at 10 seconds, the tester sample time shall be set at 10 seconds, and the tester purge time shall be set at 9 seconds.

5.5. A total of 3 filters shall be tested against the DOP liquid aerosol. Each filter shall be instantaneously loaded and evaluated.

UNCONTROLLED COPY

5.5.1. Any filter that exceeds the specified limit shall be remounted and retested to ensure that leakage was not caused by a mounting leak. If retesting eliminates the leakage, that filter shall be considered an invalid sample and another filter shall be tested in its place.

5.6. The penetration of the 3 filters shall be measured and recorded.

6. <u>PASS/FAIL CRITERIA</u>

6.1. The legal basis for passing this test is set forth in 42 CFR, Part 84, Subpart G, Section 84.63(a)(c)(d) and Subpart KK, Section 84.1151(a)(c); except that the flow requirements of Section 84.1151(a)(c) are not used. In their place, flow requirements specified in Section 84.1156(c)(2) are applied to the extent possible.

6.2. The total leakage for the connector and filter shall not exceed 0.03 percent of the ambient DOP concentration for any test sample.

7. <u>RECORDS/TEST SHEETS</u>

7.1. Record the test data in a format that shall be stored and retrievable.

8. <u>ATTACHMENTS</u>

8.1. Data Sheet

8.2. Test Setup

UNCONTROLLED COPY

8.1. Data Sheet

National Institute for Occupational Safety and Health
Respirator Branch
Test Data Sheet

NIOSH

Task Number:
Test:
Manufacturer:
Item Tested:

Reference No.:
STP No.:

Filter	Flow Rate	Maximum Allowable Percent Leakage	Actual Percent Leakage	Result

Overall Result:

Signature:

Date: ______________

Engineering Technician

UNCONTROLLED COPY

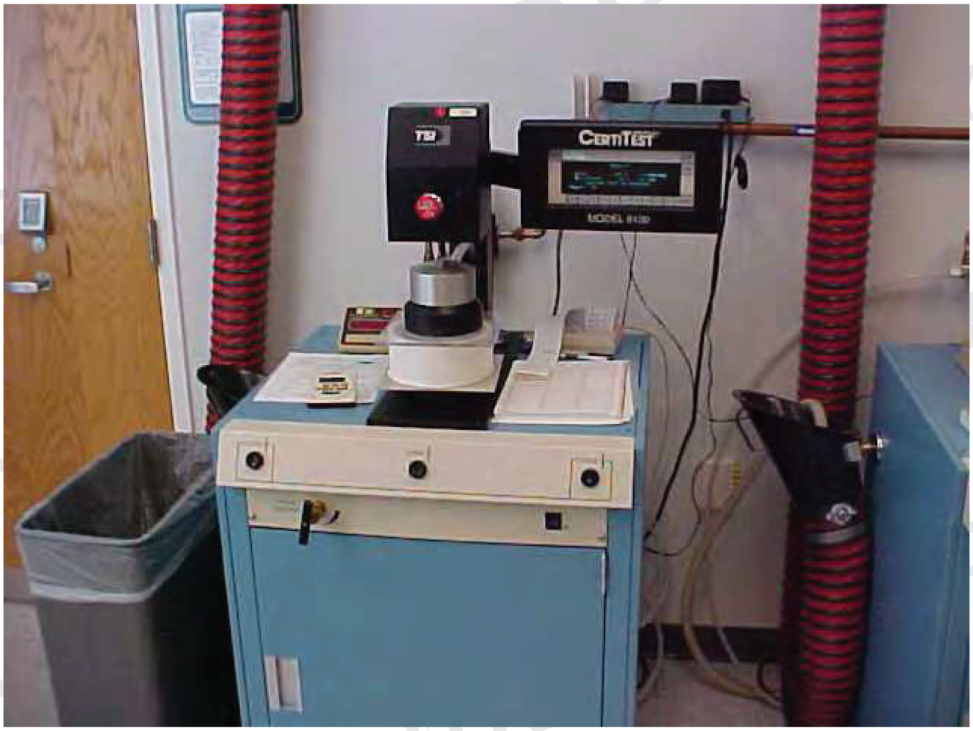

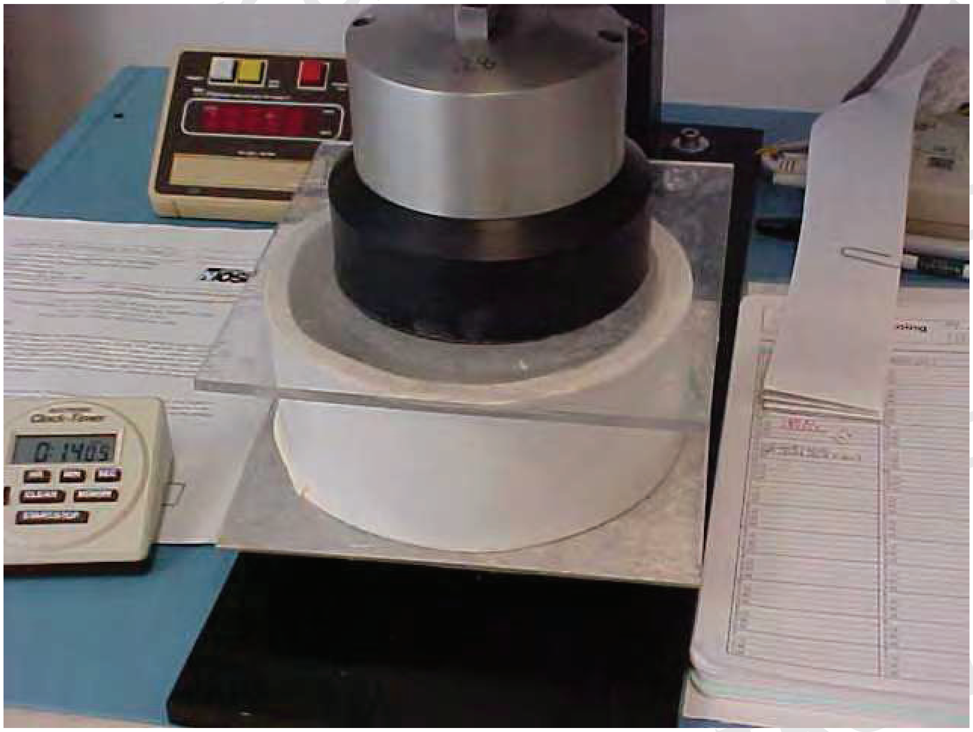

8.2. Test Setup

UNCONTROLLED COPY

Revision History

Revision	Date	Reason for Revision	
1.0	7 March 2004	Historic document	
1.1	1 June 2005	Update header and format to reflect lab move from Morgantown, WV No changes to method	
2.0	14 April 2008	Section	Change
		3.2.	List of Work Instructions added
		4.	Requirements and data for precision and accuracy added
		5.	Editorial changes to clarify procedures
		5.1.2.	Example calculation for challenge concentration added
		5.4.	Clarifications to test flow in light of the maximum total test flow of 96 lpm. A table of appropriate flow values is added.
		All	Editorial changes to improve clarity throughout

UNCONTROLLED COPY

more in any one year. Though this rule will not result in such an expenditure, we do discuss the effects of this rule elsewhere in this preamble.

F. Environment

We have analyzed this rule under Department of Homeland Security Directive 023–01, Rev. 1, associated implementing instructions, and Environmental Planning COMDTINST 5090.1 (series), which guide the Coast Guard in complying with the National Environmental Policy Act of 1969 (42 U.S.C. 4321–4370f), and have determined that this action is one of a category of actions that do not individually or cumulatively have a significant effect on the human environment. This rule involves an RNA lasting a minimum amount of time on the Savannah River when a LNG tankship in excess of heel is transiting the area or moored at the LNG facility. It is categorically excluded from further review under paragraph L60(a) of Appendix A, Table 1 of DHS Instruction Manual 023–01–001–01, Rev. 1. A Record of Environmental Consideration supporting this determination is available in the docket where indicated under **ADDRESSES**.

G. Protest Activities

The Coast Guard respects the First Amendment rights of protesters. Protesters are asked to call or email the person listed in the **FOR FURTHER INFORMATION CONTACT** section to coordinate protest activities so that your message can be received without jeopardizing the safety or security of people, places, or vessels.

List of Subjects in 33 CFR Part 165

Harbors, Marine safety, Navigation (water), Reporting and recordkeeping requirements, Security measures, Waterways.

For the reasons discussed in the preamble, the Coast Guard amends 33 CFR part 165 as follows:

PART 165—REGULATED NAVIGATION AREAS AND LIMITED ACCESS AREAS

■ 1. The authority citation for part 165 continues to read as follows:

Authority: 46 U.S.C. 70034, 70051; 33 CFR 1.05–1, 6.04–6, and 160.5; Department of Homeland Security Delegation No. 0170.1.

■ 2. Amend § 165.756 by:
■ a. In paragraph (b), removing the definitions for ''Fire Wire'', ''Made-up'', and ''Make-up'';
■ b. Revising paragraphs (d)(1)(iii)(D), (d)(2) and (3); and
■ c. Removing paragraphs (d)(4), (d)(5) and (d)(6).

The revisions read as follows:

§ 165.756 Regulated Navigation Area; Savannah River, Georgia.

* * * * *

(d) * * *

(1) * * *

(iii) * * *

(D) While transiting the RNA, LNG Tankships of cargo capacity over 120,000 m^3, carrying LNG in excess of heel, shall have a minimum of two escort towing vessels with a minimum of 100,000 pounds of bollard pull, 4,000 horsepower, and capable of safely operating in the indirect mode. At least one of the towing vessels shall be FiFi Class 1 equipped.

(2) *Requirements while LNG tankships are moored inside the LNG facility slip.* (i) An LNG Tankship of cargo capacity over 120,000 m^3, moored inside the LNG facility slip shall have two standby towing vessels with a minimum capacity of 100,000 pounds of bollard pull, 4,000 horsepower, and the ability to operate safely in the indirect mode. At least one of the towing vessels shall be FiFi Class 1 equipped. The standby towing vessels shall take appropriate action in an emergency.

(ii) If two LNG tankships of cargo capacity over 120,000 m^3 are moored inside the LNG facility slip, each vessel shall provide a standby towing vessel that is FiFi Class 1 equipped with a minimum capacity of 100,000 pounds of bollard pull and 4,000 horsepower that is available to assist.

(3) *Requirements for other vessels while within the RNA.* (i) Vessels 1,600 gross tons or greater shall at a minimum, transit at bare steerageway when within an area 1,000 yards on either side of the LNG facility slip to minimize potential wake or surge damage to the LNG facility and vessel(s) within the slip.

(ii) Vessels 1,600 gross tons or greater shall make a broadcast on channel 13 at the following points on the Savannah River:

(A) Buoy ''33'' in the vicinity of Fields Cut for inbound vessels;

(B) Buoy ''53'' in the vicinity of Fort Jackson for outbound vessels.

(iii) Vessels 1,600 gross tons or greater shall not meet nor overtake within the area adjacent to either side of the LNG facility slip when an LNG tankship is present within the slip.

(iv) Except for vessels involved in those operations noted in paragraph (c) of this section entitled Applicability, no vessel shall enter the LNG facility slip at any time without the permission of the Captain of the Port. The Coast Guard will issue a Broadcast Notice to Mariners on channel 16 upon enforcement of this RNA.

* * * * *

Dated: March 27, 2020.

E.C. Jones,

Rear Admiral, U.S. Coast Guard, Commander, Seventh Coast Guard District.

[FR Doc. 2020–06894 Filed 4–13–20; 8:45 am]

BILLING CODE 9110–04–P

DEPARTMENT OF HEALTH AND HUMAN SERVICES

42 CFR Part 84

[Docket No. CDC–2020–0036; NIOSH–335]

RIN 0920–AA69

Approval Tests and Standards for Air-Purifying Particulate Respirators

AGENCY: Centers for Disease Control and Prevention, HHS.

ACTION: Interim final rule with comment.

SUMMARY: The Department of Health and Human Service (HHS) is publishing this interim final rule to update the regulatory requirements used by the Centers for Disease Control and Prevention's (CDC) National Institute for Occupational Safety and Health (NIOSH) to test and approve air-purifying particulate respirators for use in the ongoing public health emergency. With this rulemaking, parallel performance standards are added to existing regulatory requirements for PAPRs to allow for the approval of respirators in a new class, PAPR100, that may be better suited to the needs of workers in the healthcare and public safety sectors currently experiencing a shortage of air-purifying particulate respirators due to Coronavirus Disease 2019 (COVID–19), the disease caused by severe acute respiratory syndrome coronavirus 2 (SARS-CoV–2). This rulemaking also consolidates the technical standards for all types of air-purifying particulate respirators into one subpart, and standards pertaining to obsolete respirators designed for dust, fume, and mist; pesticide; and paint spray are removed from the regulation entirely. This rulemaking will have no substantive impact on the continued certification testing and approval by the NIOSH National Personal Protective Technology Laboratory of existing PAPR class HE (high-efficiency series) respirators or non-powered air-purifying particulate respirators, including N95 filtering facepiece respirators, currently in demand by healthcare workers and emergency responders. NIOSH expects

that the addition of PAPR100 devices to the marketplace will help to relieve the current high demand for possibly hundreds of thousands of additional particulate filtering facepiece respirators needed specifically for healthcare and emergency medical response settings.

DATES: This rule is effective on April 14, 2020. Comments must be received by August 12, 2020.

ADDRESSES:

Written comments: Comments may be submitted by any of the following methods:

- *Federal eRulemaking Portal: http://www.regulations.gov.* Follow the instructions for submitting comments to the docket.
- *Mail:* NIOSH Docket Office, Robert A. Taft Laboratories, MS–C34, 1090 Tusculum Avenue, Cincinnati, OH 45226.

Instructions: All submissions received must include the agency name (Centers for Disease Control and Prevention, HHS) and docket number (CDC–2020–0036; NIOSH–335) or Regulation Identifier Number (0920–AA69) for this rulemaking. All relevant comments, including any personal information provided, will be posted without change to *http://www.regulations.gov.* For detailed instructions on submitting public comments, see the ''Public Participation'' heading of the **SUPPLEMENTARY INFORMATION** section of this document.

FOR FURTHER INFORMATION CONTACT: Jeffrey Palcic, NIOSH National Personal Protective Technology Laboratory (NPPTL), Pittsburgh, PA, (412) 386–5247 (this is not a toll-free number). Information requests can also be submitted by email to *NIOSHregs@cdc.gov.*

SUPPLEMENTARY INFORMATION:

I. Public Participation

Interested parties may participate in this rulemaking by submitting written views, opinions, recommendations, and data. Comments received, including attachments and other supporting materials, are part of the public record and subject to public disclosure. Any information in comments or supporting materials that is not intended to be disclosed should not be included. Comments may be submitted on any topic related to this interim final rulemaking, including the following:

- What operational and/or functional characteristics should be considered in establishing a standard for a healthcare PAPR?
- Should there be more than one class of healthcare PAPR, for example, surgical versus non-surgical?

II. Statutory Authority

Pursuant to the Occupational Safety and Health (OSH) Act of 1970 (Pub. L. 91–596), the Organic Act of 1910 (Pub. L. 179), and the Federal Mine Safety and Health Act of 1977 (Pub. L. 91–173 (codified at 30 U.S.C. 842(h), 844, 957)), NIOSH is authorized to approve respiratory equipment used in mines and other workplaces for the protection of employees potentially exposed to hazardous breathing atmospheres. The Occupational Safety and Health Administration (OSHA) requires U.S. employers to supply NIOSH-approved respirators to their employees whenever the employer requires the use of a respirator. (29 CFR 1910.134(d))

III. Background

A. Introduction

Air-purifying respirators use either filters, cartridges, or canisters (or combinations of filters and cartridges or filters and canisters), to protect users from gases; vapors; aerosols, including viruses capable of being transmitted by aerosolized droplets; and other contaminants in the air. Since these respirators simply purify the ambient atmosphere and do not provide an independent supply of breathing air to the wearer, most types cannot be used in atmospheres that are immediately dangerous to life and health (IDLH).[1] Air-purifying particulate respirators, a subclass of air-purifying respirators, are approved by NIOSH pursuant to 42 CFR part 84. Currently, testing and performance standards for non-powered air-purifying particulate respirators are codified in part 84, subpart K; standards for powered air-purifying particulate respirators are codified in subpart KK.

Non-powered air-purifying particulate respirators include filtering facepiece respirators and elastomeric half- and full-facepiece respirators, and are used in a very wide variety of work settings.

Powered air-purifying particulate respirators (PAPRs) are used in many similar work settings and are distinguished from the non-powered type by the powered blower that moves air through the attached filters, canisters, and/or cartridges. This respirator type comes in a variety of sizes, weights, and mounting configurations. PAPRs play an integral role in respiratory protection programs across multiple sectors, including general industry, healthcare, and police operations.

[1] With the exception of gas masks designed for escape from IDLH atmospheres. *See* 42 CFR 84, subpart I—Gas Masks.

Current regulatory standards provide for the NIOSH approval of high-efficiency (HE) particulate filters which are incorporated into PAPRs. The NIOSH National Personal Protective Technology Laboratory has determined the need for increasing the utility of PAPRs in the workplace and offering a wider array of options for today's work practices. Although the current PAPR approval program has proven protections, these interim requirements offer the potential to extend the same proven level of protection to smaller, lighter systems which may be more comfortable to wear, as discussed below.

B. PAPR Certification and Approval

NIOSH currently approves PAPRs under 42 CFR part 84, *Approval of Respiratory Protective Devices.* Within part 84, subpart KK, *Dust, Fume and Mist; Pesticide; Paint Spray; Powered Air-Purifying High Efficiency Respirators and Combination Gas Masks,* specifies testing and certification requirements for PAPRs with high-efficiency particulate filters. NIOSH reviews and approves such respirators for use, for example, by industrial, healthcare, and public safety workers.

C. Scope of the Rulemaking

This rulemaking applies to air-purifying particulate respirators and gas and vapor respirators which also incorporate a particulate filter. NIOSH is (1) consolidating all air-purifying, particulate respirator requirements, whether powered or non-powered, into subpart K; (2) eliminating unneeded and archaic parts of the standard related to PAPRs which were left in place since the 1995 promulgation of part 84; and (3) better aligning PAPR particulate filter testing for a new class of PAPR with the requirements for non-powered particulate respirators which were established in the 1995 rulemaking.

With this rulemaking, a new class of PAPR is established, PAPR100, in parallel with the current PAPR class HE, to open opportunities for designs offering the characteristics desired by many end-users, as revealed through user-sector input following the public meetings in 2003–2008 and a 2014 Institute of Medicine workshop, discussed below. PAPRs tested to the current requirements relocated from subpart KK are designated series ''HE''; those requirements are otherwise unchanged. PAPR100s tested to the new alternative testing and approval requirements are designated either series ''PAPR100–N,'' which is not for use against oil-based aerosols, or

''PAPR100–P,'' which is strongly resistant to oil aerosols.

Requirements for the current class HE are unchanged because those devices have a proven track record and widespread use. The existing HE requirements result in the approval of PAPRs that are well-suited to heavy industry settings where the particulates of concern may be dense in terms of their airborne concentration. In those settings, the PAPR is often unavoidably challenged to remove a large quantity of larger, non-respirable particles while it is doing the important work of removing the much smaller, but much more hazardous, respirable-sized particles. While the existing silica dust test specified in subpart KK demonstrates a portion of the unit's ability to remove the respirable-sized particles, it is a very good test to demonstrate the PAPR's ability to provide ongoing filtration across the wider aerosol size spectrum in these ''dirtier'' industrial settings. With this rulemaking, NIOSH is promulgating a new standard for the new class PAPR100, which replaces the silica dust test with a sodium chloride aerosol when testing PAPR100–N series filters, and with a dioctyl phthalate aerosol when testing PAPR100–P series filters. NIOSH will not designate either class specifically for industrial or non-industrial use, but it is thought that the PAPR class HEs will continue to be the design of choice in industrial settings. Since protections provided by the current class HE respirators are considered equivalent to the protections expected by the new PAPR100 devices, respiratory safety continues to be assured, regardless of the setting.

This rulemaking also eliminates the requirements for other obsolete types of respirators, including dust, fume, and mist; pesticide; and paint spray respirators identified in current subpart KK. Subpart KK is removed from part 84 in its entirety.

D. Need for Rulemaking

PAPRs are often used in high-hazard procedures in the healthcare setting because they are designed to filter chemicals, blood-borne pathogens, and aerosol-transmissible diseases. However, the size and weight of the PAPRs approved under the current regulations has been said to limit their widespread adoption in healthcare and by first responders. The current requirements for PAPR class HE (high-efficiency series) contained in 42 CFR part 84 were established in 1972 primarily for more industrial-type uses and exposures, such as mining and milling operations. The silica dust loading test is currently incorporated among the requirements which determine the PAPR filter efficiency. In order to pass the silica dust test, current NIOSH-approved PAPRs must provide a high flow of breathing air against a highly loaded filter for a duration of 4 hours. This generally results in approved PAPRs having blowers and batteries which may be inconveniently large, heavy, or both. Respirator designers and end-users have expressed a desire for greater latitude in the regulatory requirements in order to reduce the bulk and weight of currently approved PAPR class HE devices, given the advances in modern battery and sensor technology that would allow for smaller, lighter designs with service durations continuously monitored by required flow-detection devices.

During the past 20 years, PAPRs have played an increasing role in respiratory protection programs in the United States in sectors beyond general industry, including healthcare. PAPRs are also frequently considered for public safety and other specialized industrial uses. The 2002 Severe Acute Respiratory Syndrome (SARS), the 2009 H1N1 influenza, and the 2014 Ebola virus outbreaks ushered in more extensive use of respiratory protection, and specifically PAPRs, for today's 18 million healthcare workers.

In a 2014 assessment designed to quantify the amount of personal protective equipment held in U.S. acute care hospitals, the Association of States and Territorial Health Officials (ASTHO) estimated that acute care hospitals across the United States had no more than 83,196 PAPRs on-hand in 2012 compared with 114,694,159 N95s, demonstrating that the currently approved PAPRs are not as widely-used in healthcare as the N95s.[2] However, the Association for Professionals in Infection Control and Epidemiology (APIC) reported that healthcare employers are expected to increase the relative number of PAPRs used in healthcare as the devices become less expensive and lighter.[3] PAPRs have a number of advantages over N95 filtering facepiece respirators, including that they are reusable and can be cleaned and disinfected, loose-fitting PAPR do not need to be fit tested and often can be worn with facial hair, and have a higher assigned protection factor (as determined by the Occupational Safety and Health Administration in the Department of Labor). Designs not requiring fit testing are expected to be especially advantageous in a public health emergency, such as the Coronavirus Disease 2019 (COVID–19) response, by saving resources including both person-hours and the need to fit test multiple makes and models to find the right fit for an individual worker. Loose-fitting PAPR designs are also typically equipped with a head covering that delivers filtered air over the user's entire head, including the eyes and hair, thus offering greater overall protection from contact with any airborne infectious agents.

Healthcare workers and first responders are on the front line of efforts to contain COVID–19, the disease caused by severe acute respiratory syndrome coronavirus 2 (SARS-CoV–2). The virus is thought to spread primarily by person-to-person contact through respiratory droplets produced when an infected person coughs or sneezes; it may also spread through contact with contaminated surfaces or objects. The ease of SARS-CoV–2 transmission has resulted in a surge in hospitalizations in many jurisdictions, resulting in a well-documented shortage of personal protective equipment, especially respiratory protection, for healthcare workers and emergency responders. An APIC survey conducted March 23–24, 2020 found that 20 percent of respondents indicated they do not have any respirators and 61 percent of respondents indicated they are almost out of respirators. Only 18 percent of respondents said they have a sufficient number of respirators.[4]

Between March 16 and April 3, 2020, five potential approval holders seeking to develop PAPRs to support the COVID–19 response solicited NIOSH to explore the possibility of producing PAPRs for healthcare and emergency responders to increase the inventory of PAPRs across the nation. NIOSH expects that PAPR100s will be purchased to replace the current inventory of larger class HE devices designed for industrial use, as well as to substitute for the use of disposable N95 filtering facepiece respirators which require fit testing for effective use. NIOSH expects that the addition of PAPR100 devices to the marketplace will help to relieve the current high demand for possibly hundreds of

[2] ASTHO, *Assessment of Respiratory Personal Protective Equipment in U.S. Acute Care Hospitals—2012* (2014).

[3] *See* APIC public comment submitted to NIOSH Docket-272 for National Institute for Occupational Safety and Health, CDC, *Respiratory Protective Devices Used in Healthcare; Notice of Request for Information and Comment,* 79 FR 14515 [March 14, 2014].

[4] APIC, *Protecting Healthcare Workers During the COVID–19 Pandemic: A Survey of Infection Preventionists* (March 27, 2020), *https://apic.org/wp-content/uploads/2020/03/Protecting-Healthcare-Workers-Survey_Report_3_26_20_Final.pdf.*

thousands of additional particulate filtering facepiece respirators designed specifically for healthcare settings.

E. History of the PAPR100 Concept

NIOSH held a series of public meetings from 2003 through 2008 to discuss technical issues regarding a new PAPR concept.[5] Participants raised issues regarding the existing PAPR certification requirements and offered input on the need to eliminate the silica dust test and incorporate warnings for low air flow, pressure, and/or battery life.

In response to the growing number of PAPRs in healthcare, NIOSH sponsored an Institute of Medicine (IOM) workshop on the ''Use and Effectiveness of PAPRs in Healthcare'' in 2014.[6] The intent of the workshop was to assist NIOSH with prioritizing and updating approval requirements for NIOSH-approved PAPRs suitable for use in the healthcare sector. IOM workshop participants included government agencies, healthcare institutions, professional associations, respirator manufacturers, and unions representing healthcare workers. A general finding from the IOM workshop stated that current PAPR requirements are not always suitable for the healthcare work environment. Workshop participants indicated that powered air-purifying respirators should have the following attributes:

- Suitable for use in sterile field;
- Good visibility and communication;
- Ease of donning, doffing, and cleaning;
- Variable flows based on work rates;
- Smaller and less bulky;
- Sensors and alarms that monitor flow and power; and
- Training materials as part of certification.

In addition to the IOM workshop, NIOSH reached out to the International Safety Equipment Association (ISEA) and 10 manufacturers of NIOSH-approved PAPRs in August and September 2016 to better understand how current requirements impact PAPR designs and how today's technologies are being integrated into PAPR designs. According to the input NIOSH received, the aerosol threat in the healthcare setting, as compared with the industrial settings the current PAPR class HE requirements in part 84 are designed to address, is composed mainly of respirable-sized (or smaller) particles, with practically no other larger particles in the mix. Therefore, the ability to continue to provide needed air flow against high total filter loading is not a necessary consideration for PAPRs suitable for use in the healthcare setting. These experts indicated the following main areas of concern:

1. Silica dust testing adds to the size and weight of PAPR systems.
2. Silica dust test equipment is outdated and the test is a challenge to reproduce, not representative of today's workplace dust conditions, and requires operational safeguards to avoid the test operator's hazardous exposure to silica dust.
3. If the PAPR continuously monitors critical conditions such as flow, pressure, and battery life, the silica dust test would not be needed since the complete system is also evaluated with a quantitative human subject testing (corn oil test).
4. Technologies such as sensors and alarms for monitoring airflow rate, battery life, facepiece pressure, and other critical components are being integrated into many of today's PAPR designs. The current PAPR requirements prevent these technologies from being fully deployed.

NIOSH presented its new PAPR concept at the 2016 biennial International Society for Respiratory Protection (ISRP) conference in Yokohama, Japan and the 2017 meetings of the ISRP Americas Section in Pittsburgh, Pennsylvania and the National Academies Standing Committee on Personal Protective Equipment for Workplace Safety and Health. Attendees of these meetings generally supported the concepts presented.

By modifying and replacing some of the current PAPR requirements, NIOSH would enable manufacturers to take advantage of contemporary technology that could result in smaller and lighter-weight PAPRs having the same effective particulate protections while increasing workplace utility for today's diverse workforces. The addition of requirements for NIOSH-approved PAPRs intended for healthcare and other settings with lower overall particulate presence would allow stakeholders to incorporate additional technologies such as integrated circuits, sensors, batteries, motors, plastics, and fabrics to improve PAPR designs intended to be used in cleaner settings, such as healthcare.

F. Impact on Rulemaking and Other Activities of OSHA

The interim final rule would not require OSHA to make any changes to 29 CFR 1910.134, the OSHA respiratory protection requirements.

IV. Issuance of an Interim Final Rule With Immediate Effective Date

Rulemaking under the Administrative Procedure Act (APA) generally requires a public notice and comment period and consideration of the submitted comments prior to promulgation of a final rule (5 U.S.C. 553). However, the APA provides for exceptions to its notice and comment procedures when an agency finds that there is good cause for dispensing with such procedures on the basis that they are impracticable, unnecessary, or contrary to the public interest. In accordance with the provisions in 5 U.S.C. 553(b)(B), HHS finds good cause to waive the use of prior notice and comment procedures for this interim final rule and to make this action effective immediately.

This interim final rule amends 42 CFR part 84 to allow respirator manufacturers to produce an equally protective or equivalent new class of PAPR, the PAPR100, including both N-series and P-series particulate respirators, designed for use in healthcare or other workplace settings that will benefit the most from smaller, lighter devices. HHS has determined that it is impracticable to use prior notice and comment procedures for this interim final rule because of the ongoing public health emergency. As discussed above, respirator manufacturers have participated in discussions with NIOSH about the need for these new standards and are generally supportive of this effort. Recently, some manufacturers have notified NIOSH that they are ready to submit approval applications for PAPR100s that would be employable in the current public health emergency as soon as the effective date of this interim final rule. Thus, HHS is waiving the prior notice and comment procedures in the interest of protecting the health of healthcare workers and emergency responders who are on the front lines of the current public health emergency as soon as possible.

Under 5 U.S.C. 553(d)(3), HHS also finds good cause to make this interim final rule effective immediately. As stated above, in order to protect the health of healthcare workers and emergency responders, it is necessary that HHS act quickly to amend the existing standards in 42 CFR part 84 to allow NIOSH to approve a new class of PAPR suitable for use in healthcare settings. The addition of this new class of respirator to the market will improve safety of healthcare workers because it will result in the development of PAPRs that are less bulky, less noisy, and more suitable for use in healthcare and emergency response settings to meet the

[5] Transcripts of the public meetings as well as presentations and submissions from interested parties are available in NIOSH Dockets 008 and 008a.

[6] IOM [2015], *The Use and Effectiveness of Powered Air Purifying Respirators in Health Care: Workshop Summary* (National Academies Press: Washington, DC).

immediate needs of those treating patients during the COVID–19 pandemic. The cost of these devices is expected to be lower than the costs of PAPRs currently on the market. Loose-fitting PAPRs do not require fit testing, and because the devices are reusable and have a higher filter efficiency and higher assigned protection factor, thus they are a cost-effective alternative to other respiratory protective devices currently on the market. Because these PAPRs are reusable, it is likely that 1 percent of the stock of PAPRs would be required compared to that of single-use items such as the N95 filtering facepiece respirator, assuming the ability to reuse a PAPR one hundred times. Healthcare organizations using PAPRs in healthcare settings have reported cleaning their PAPR filters for several years prior to replacement, which is well beyond the 1 percent estimate.

While amendments to part 84 are effective on the date of publication of this interim final rule, we request public comment on this rule. After full consideration of public comments, HHS will publish a final rule with any necessary changes. (See Section I. Public Participation, above.)

V. Summary of Interim Final Rule

As discussed above, this interim final rule consolidates all air-purifying particulate respirator requirements in 42 CFR part 84, subpart K, and establishes alternative requirements for the testing and approval of class PAPR100 respirators designed for use in settings such as healthcare, public safety, and other workplaces that require or otherwise place a premium on the use of smaller, lighter devices. Other existing sections in part 84 that reference subpart KK are updated as necessary.

The table directly below matches the reorganized part 84, subpart K, with the originating sections in the current regulation. These changes are discussed in full below the table.

REORGANIZATION AND SECTION TITLE AMENDMENTS

Interim final rule section		Originating section	
84.170(a)	Non-powered air-purifying particulate respirators (series N, R, and P).	84.170	Non-powered air-purifying particulate respirators; description.
84.170(b)	Powered air-purifying particulate respirators (PAPR classes HE and PAPR100).	84.1100(d)	Scope and effective dates—powered air-purifying particulate respirator.
		84.1130(a)(4)	Respirators, description—air-purifying respirators.
			New for PAPR100 class.
84.171	Required components and attributes	84.171	Non-powered air-purifying particulate respirators; required components.
		84.1131	Respirators; required components.
84.171(a)	Respiratory inlet covering	84.175	Half-mask facepiece, full facepiece, hoods, helmets, and mouthpieces; fit; minimum requirements.
		84.171(a)	Non-powered air-purifying particulate respirators; required components.
		84.1135	Half-mask facepiece, full facepiece, hoods, helmets, and mouthpieces; fit; minimum requirements.
		84.1136	Facepieces, hoods, and helmets; eyepieces; minimum requirements.
84.171(b)(1)	Filters for non-powered respirators	84.179	Non-powered air-purifying particulate respirators; filter identification.
84.171(b)(2)	Filters for powered respirators	84.1130(a)(4)	Respirators; description—Powered air-purifying particulate respirators; filter identification.
84.171(c)	Valves	84.177 84.1137	Inhalation and exhalation valves; minimum requirements.
84.171(d)	Head harness	84.178, 84.1138	Head harness; minimum requirements.
84.171(e)	Breathing tube	84.172 84.1132	Breathing tubes; minimum requirements.
84.171(f)	Drink tube		New.
84.171(g)	Container	84.174 84.1134	Respirator containers; minimum requirements.
84.171(h)	Harness	84.173 84.1133	Harnesses; Installation and construction; minimum requirements.
84.171(i)	Attached blower	84.1156(f)	Minimum air flows.
84.171(j)	Low-flow warning device		New.
84.172	Airflow resistance test	84.180	Airflow resistance tests.
		84.1156(a)(1) and (2).	Pesticide respirators; performance requirements; general—breathing resistance test.
		84.1157(a)	Chemical cartridge respirators with particulate filters; performance requirements; general—breathing resistance test.
84.173	Exhalation valve leakage test	84.182 84.1150	Exhalation valve leakage test; minimum requirements.
84.174	Filter efficiency level determination test—non-powered series N, R, and P filtration.	84.181	Non-powered air-purifying particulate filter efficiency level determination.
84.175	Instantaneous filter efficiency level determination test—PAPR series HE, PAPR100–N, and PAPR100–P filtration.	84.1151	DOP filter test.
		84.1156(c)(2)	Pesticide respirators; performance requirements; general—silica dust test.
84.176(a)	Isoamyl acetate (IAA) fit test	84.1156(b)(5)	Pesticide respirators; performance requirements; general—isoamyl acetate tightness test.
84.176(b)	Generated Aerosol		New.
84.177	Total noise level test—PAPR classes HE and PAPR100.	84.1139	Air velocity and noise levels; hoods and helmets.

REORGANIZATION AND SECTION TITLE AMENDMENTS—Continued

Interim final rule section			
84.178	Breath response type, airflow resistance test—PAPR classes HE and PAPR100.		New.
84.179	Silica dust loading test—PAPR series HE filtration.	84.1144	Silica dust test for dust, fume, and mist respirators; single-use or reusable filters; minimum requirements.
		84.1152	Silica dust loading test.
84.180	Particulate loading test—PAPR series PAPR100–N and PAPR100–P filtration.		New.
84.181	Communication performance test—class PAPR100.		New.

Section 84.2 Definitions

In this existing section, located in 42 CFR part 84, subpart A, HHS adds definitions for the terms ''respiratory inlet covering,'' ''tight fitting,'' ''loose fitting,'' and ''warning device.''

Section 84.126 Canister Bench Tests; Minimum Requirements

In this existing section in subpart I—Gas Masks, a new paragraph (f) specifies that PAPRs designed with one or more canisters and particulate filters must meet the end-of-service-life requirements both as received from the applicant and after being equilibrated at room temperature.

Section 84.207 Bench Tests; Gas and Vapor Tests; Minimum Requirements; General

In this existing section in subpart L—Chemical Cartridge Respirators, a new paragraph (h) specifies that PAPRs designed with one or more canisters and particulate filters must meet the end-of-service-life requirements both as received from the applicant and after being equilibrated at room temperature.

Subpart K—Air-Purifying Particulate Respirators

Subpart K is retitled from ''Non-Powered Air-Purifying Particulate Respirators'' to ''Air-Purifying Particulate Respirators.'' The intent of the new title is to properly indicate the broadened scope of the subpart, which includes the requirements for both non-powered and powered air-purifying particulate respirators.

Section 84.170 Air-Purifying Particulate Respirators; Description

This section provides a general description of air-purifying particulate respirators as a class of respirator. It is intended to inform the public and to serve as a legal and practical definition for the purposes of the NIOSH respirator approval program.

Paragraphs (a)(1), (2), and (3), which describe non-powered devices, remain substantively unchanged from the existing language. New paragraphs (b)(1), (2), and (3) describe PAPRs. Specifically, paragraph (b)(1) provides a general description of PAPRs and paragraph (b)(2) indicates that PAPRs are classified into one of two PAPR classes, HE or PAPR100, and one of three filter series, ''HE,'' ''PAPR100–N,'' and ''PAPR100–P.'' Paragraph (b)(3) establishes that the minimum efficiency level for filters employed as part of powered respirator configurations is 99.97 percent for all three filter series, HE (high-efficiency), PAPR100–N, or PAPR100–P.

Requirements for two series of filters have been established for the PAPR100 class to give manufacturers greater flexibility in designing these devices. The PAPR100–P series filter requirements are established to provide a filter that, like the existing PAPR class HE (high-efficiency series) filter, is suitable for use against all aerosols, including those which are comprised of oils.

The PAPR100–N series filter, which is not intended to be used against oil-based aerosols, has also been added to allow for greater use of electrostatic filter media. New filter efficiency requirements in § 84.180 are intended to allow manufacturers to optimize PAPR100–N series filters for environments with very low concentrations of non-oil based (solid- or water-based) aerosols where disposal of the filter after each use is preferred over extended use. The minimum filtration efficiency for the two new series of PAPR filters is maintained at 99.97 percent, the minimum filtration efficiency of the existing and ongoing HE series filters.

Section 84.171 Required Components and Attributes

The title of this existing section is revised to describe the requirements for components and attributes that apply to both powered and non-powered air-purifying particulate respirators. The regulatory language itself is revised to replace terminology such as ''facepiece, mouthpiece with nose clip, hood, or helmet'' with ''respiratory inlet covering''; ''half-mask facepieces and full facepieces'' with ''tight-fitting respiratory inlet coverings''; and ''hoods and helmets'' with ''loose-fitting respiratory inlet coverings.'' The entire section is revised to not only include a list of the required components, but to include the required design attributes of those components.

Paragraph (a) specifies the required attributes for the respiratory inlet covering, currently described in §§ 84.175 and 84.1135.

Paragraph (b)(1) addresses the filter unit, currently described in § 84.179 for non-powered devices; paragraph (b)(2) includes new provisions specifying that powered devices must be labeled as series HE (high-efficiency) or series PAPR100–N or –P.

Paragraph (c) addresses valves, currently described in §§ 84.177 and 84.1137.

Paragraph (d) addresses the head harness, currently described in §§ 84.178 and 84.1138.

Paragraph (e) addresses the breathing tube, currently described in §§ 84.172 and 84.1132.

Paragraph (f) is new, and describes requirements for a drink tube, should the design require a drink tube.

Paragraph (g) addresses the container, currently described in §§ 84.174 and 84.1134.

Paragraph (h) addresses the harness, currently described in §§ 84.173 and 84.1133.

Paragraph (i) is moved from § 84.1156(f) to describe the airflow rate required of PAPR HE class and PAPR100 class tight-fitting and loose-fitting respiratory inlet coverings.

Finally, a new paragraph (j) requires a low-flow warning device for the new PAPR100 class respirators only. There are no requirements for PAPR warning devices in 42 CFR part 84 for class HE respirators. However, if any PAPR system is submitted for approval equipped with a warning device, NIOSH verifies that the warning functions

properly as per the manufacturer's user instructions. In accordance with this paragraph, the required PAPR100 warning must alert users to breathing air flow that falls below 115 liters per minute for tight-fitting facepieces or 170 liters per minute for loose-fitting hoods and helmets (the minimum required in § 84.175). Warning devices must also be able to be heard or otherwise detected by the wearer and must also be readily distinguishable from one another. For example, if an optional low-battery warning is included in addition to the low-flow warning, it needs to be distinguishable from the required low-flow warning. The PAPR100 warning system must also not de-energize while the unit's blower is energized (*i.e.,* power to the warning system must be prioritized), and must not switch off automatically or be able to be switched off manually. The warning should remain active until the reason for the warning is corrected.

Section 84.172 Airflow Resistance Test

This section specifies the test criteria and acceptable performance criteria for inhalation and exhalation resistance of a complete air-purifying particulate respirator. The requirements for non-powered air-purifying particulate respirators are currently specified in § 84.180 and would be consolidated in § 84.172 with requirements for PAPRs, unchanged. The existing requirements for PAPR class HE are moved from § 84.1156(a)(1) and (2) and combined into § 84.172, where the maximum airflow resistance standard for the new class PAPR100 would also be established.

Paragraph (a) addresses the inhalation and exhalation resistance of the complete air-purifying particulate respirator. This paragraph is essentially unchanged in meaning but updated from the existing language in § 84.180(a) to reflect industry standard terminology, replacing ''facepiece, mouthpiece, hood, or helmet'' with ''respiratory inlet covering.''

Paragraph (b) indicates that the airflow resistance of tight-fitting PAPRs is measured with the blower off if the model is designed not to be immediately doffed in the event of a blower failure.

Paragraph (c) maintains the current requirements in § 84.1157(a) for the maximum inhalation and exhalation resistances of complete PAPRs (both classes HE and PAPR100) and the current requirements in § 84.180(b) for non-powered air-purifying respirators.

Section 84.173 Exhalation Valve Leakage Test

This section contains the existing requirements in §§ 84.182 and 84.1150 that describe the NIOSH tests for exhalation valve leakage. The exhalation valve leakage testing is conducted on both non-powered and powered devices.

Section 84.174 Filter Efficiency Level Determination Test—Non-Powered Series N, R, and P Filtration

Text from existing section § 84.181 specifies the test criteria and acceptable performance criteria for non-powered air-purifying particulate filter efficiency levels; it is re-numbered § 84.173. This section is also re-named to clarify the content and indicate its application for all types of air-purifying particulate respirators. The word ''shall'' is replaced with ''will'' throughout the section, to clarify intent and reflect plain language principles. No substantive changes are made to the testing requirements and technical standards for filter efficiency for non-powered devices.

Section 84.175 Instantaneous Filter Efficiency Level Determination Test—PAPR Series HE, PAPR100–N, and PAPR100–P Filtration

This new section describes the NIOSH filter efficiency testing requirements for both classes of PAPR and all three particulate series filters, HE, PAPR100–N, and PAPR100–P. This instantaneous dioctyl phthalate (DOP) test is unchanged from the current § 84.1151. PAPRs are tested at the minimum required flow rates specified in § 84.1156(c)(2).

Paragraph (a) indicates that three filters from each powered air-purifying particulate respirator will have their filtration efficiency evaluated using DOP.

Paragraph (b) describes the current atmospheric concentration of DOP. The test concentration, 100 milligrams per cubic meter, is unchanged. Paragraph (b) also includes the airflow rates for tight- and loose-fitting respiratory inlet coverings currently found in § 84.1156(c)(2).

Paragraph (c) indicates that PAPRs designed with multiple filters will be tested by dividing the specified flow rate by the total number of filters.

Finally, paragraph (d) requires the filters, including holders and gaskets, when separable, to be tested while mounted on a test fixture in the manner as used on the respirator. This allows NIOSH to test the assembly in a configuration as it will actually be used.

Section 84.176 Fit Test—PAPR Classes HE and PAPR100

This section specifies the test criteria and acceptable performance criteria to fit test a complete PAPR. Two options are available to assess fit: Isoamyl acetate (IAA) or generated aerosol.

Paragraph (a) specifies the existing IAA tightness test, originally established in subpart KK, § 84.1156(a)(5). The IAA testing standard is unchanged.

Paragraph (b) describes a new generated aerosol (corn oil) test, intended as an alternative to the IAA method for those powered devices that are equipped solely with particulate filters. The corn oil quantitative fit test was developed by NIOSH, at the behest of respirator manufacturers, and has been used as a voluntary substitute test in place of the qualitative IAA test for series HE PAPRs since approximately 2008. This test utilizes a concentration of 20–40 milligrams per cubic meter of corn oil aerosol with a mass median aerodynamic diameter of 0.4 to 0.6 micrometers. Paragraph (b)(1) describes the work schedule performed by the wearer during the test. The activities that are specified in this paragraph—nodding and turning head, calisthenic arm movements, running in place, and pumping a tire pump—are used by the agency to test the facepiece fit of respirator types by simulating the types of activities workers might perform while wearing the respirator.

Paragraph (b)(2) allows NIOSH to verify that the facepiece is capable of adjustment and that the applicant's donning instructions should be followed. Paragraph (b)(3) requires that the appropriate fit factors for the applicant respirator be exceeded.

Section 84.177 Total Noise Level Test—PAPR Classes HE and PAPR100

This section replicates the testing standard for PAPR noise levels currently found in § 84.1139. The standard requires that the noise levels generated by any PAPR (*i.e.,* HE hood or helmet and any PAPR100) must not exceed 80 decibels using the A-weighting frequency response (dBA) measured at each ear location while the system operates at its maximum airflow obtainable. Today, PAPR designs include head-, neck-, and face-mounted blowers in closer proximity to the user's ears. Additionally, for class HE hood and helmet designs, the provision is revised to clarify that the noise level measurement will be taken at the entrance to the ear rather than ''inside the hood or helmet'' as the standard currently states.

Section 84.178 Breath Response Type, Airflow Resistance Test—PAPR Classes HE and PAPR100

This new section specifies the minimum test criteria for a breath-responsive PAPR. Breath-responsive PAPRs are designed to maintain a positive pressure in the facepiece to match the user's respiratory requirements. Current PAPR requirements in 42 CFR part 84 do not address these design features. Therefore, pursuant to 42 CFR 84.60 and 84.63, these types of PAPRs have been evaluated using the requirements of 42 CFR 84.157, which are applicable to certain types of atmosphere-supplying respirators.

This section specifies that the breath-responsive PAPR airflow will be measured with a breathing machine described in § 84.88(b) and (c). Paragraph (a) specifies that the minimum inhalation resistance shall be greater than zero. Paragraph (b) specifies that the maximum exhalation resistance must be less than 89 millimeters (3.5 inches) of water-column height, in accordance with current requirements in § 84.91(c) and (d).

Section 84.179 Silica Dust Loading Test—PAPR Series HE Filtration

This section contains the requirements from existing §§ 84.1144 and 84.1152, which are themselves removed from part 84 in this action. This section specifies the test criteria for the silica dust loading test of a complete powered PAPR series HE. This test procedure is not used to test PAPR100–N or –P series devices, which NIOSH expects will allow PAPR100 designs to be smaller and lighter than series HE devices. Paragraphs (a) and (f), respectively, specify the test period and flowrate as well as the amount of unretained test suspension; these testing standards are taken from § 84.1152. Paragraphs (b), (c), (d), and (e) establish the test chamber conditions and size and concentration of the test particulate.

Section 84.180 Particulate Loading Test—PAPR Series PAPR100–N and PAPR100–P Filtration

This new section adopts the existing particulate loading test for non-powered air-purifying respirators in § 84.181, applying it to both PAPR100 series filters. Paragraph (a) specifies that NIOSH will test the efficiency of 20 filters of each powered air-purifying particulate respirator model submitted for a class PAPR100 approval.

Paragraph (a)(1) specifies that NIOSH will use a sodium chloride aerosol when testing PAPR100–N series filters. Paragraph (a)(2) specifies that NIOSH will use a dioctyl phthalate or equivalent aerosol when testing PAPR100–P series filters.

Paragraph (b) requires that 20 PAPR100–N series filters be preconditioned with humid air prior to being subjected to the filtration efficiency loading test specified in paragraph (d)(1).

Paragraph (c) specifies the continuous test aerosol flow rates for the filter efficiency testing. Single filters are to be tested at a rate of 85 ± 4 liters per minute; filters used in pairs at a rate of 42.5 ± 2 liters per minute through each filter; and filters used in threes at a rate of 28.3 ± 1 liters per minute through each filter.

Paragraph (d)(1) specifies the filter efficiency test aerosol for series PAPR100–N, sodium chloride or an equivalent solid aerosol. The test conditions for the solid aerosol are specified to be at 25 ± 5 degrees Celsius. The sodium chloride aerosol specified to be used in these tests is to be neutralized to the Boltzmann equilibrium state, and the maximum concentration will not exceed 200 milligrams per cubic meter. This paragraph also specifies the particle size, and size distribution of the sodium chloride test aerosol at a count median diameter of 0.075 ± 0.020 micrometer and a standard geometric deviation not exceeding 1.86 at the specified test conditions as determined with a scanning mobility particle sizer or equivalent.

Paragraph (d)(2) specifies the filter efficiency test aerosol for series PAPR100–P, DOP or an equivalent oil liquid particulate aerosol. The test conditions for the liquid aerosol are specified to be at 25 ± 5 degrees Celsius. The DOP aerosol specified to be used in these tests is to be neutralized to the Boltzmann equilibrium state, and the maximum concentration will not exceed 200 milligrams per cubic meter. This paragraph also specifies the particle size, and sized distribution of the DOP test aerosol at a count median diameter of 0.185 ± 0.020 micrometer and a standard geometric deviation not exceeding 1.60 at the specified test conditions as determined with a scanning mobility particle sizer or equivalent.

Paragraph (e) specifies that both the solid and the liquid aerosol filtration efficiency test must continue until minimum efficiency is achieved or until an aerosol mass of 200 ± 5 milligrams has contacted the filter. This paragraph further specifies that for PAPR100–P series filters, if the filter efficiency is decreasing when the 200 ± 5 mg challenge point is reached, the test shall be continued until there is no further decrease in efficiency.

Paragraph (f) requires the efficiency of the filter (*i.e.,* the amount of aerosol particles that are removed by the filter) to be monitored and recorded throughout the test period by a suitable forward-light-scattering photometer or equivalent instrumentation.

Paragraph (g) requires the minimum filter efficiency for each of the 20 filters to be determined and recorded. The minimum efficiency of each tested filter must be greater than or equal to 99.97 percent for both PAPR100–N and PAPR100–P series filters.

Section 84.181 Communication Performance Test—PAPR Class PAPR100

This new section specifies testing criteria for PAPR communication performance. The 2014 IOM workshop highlighted the limitations posed by PAPRs with regard to communication with patients, potentially compromising patient safety. This test is intended to address healthcare, first responders, and other workers' needs for PAPR100s designed and tested to ensure a PAPR's ability to meet a minimum communication performance level of speech conveyance and intelligibility.

Paragraph (a) requires that PAPR100s are designed to allow minimum communication while being worn.

Paragraph (b) specifies that the Modified Rhyme Test (MRT) will be used to conduct the test. The MRT consists of lists of 50 monosyllabic, phonetically-balanced words and evaluates a listener's ability to comprehend single words spoken by the respirator wearer.

Paragraph (c) specifies that for each MRT trial the overall performance rating is calculated. The performance rating is the ratio of the number of correct responses to the number of incorrect responses with and without a respirator being worn. To obtain a passing score, the PAPR100 must obtain an average overall performance rating greater than or equal to 70 percent.

VI. Regulatory Assessment Requirements

A. Executive Order 12866 (Regulatory Planning and Review) and Executive Order 13563 (Improving Regulation and Regulatory Review)

Executive Orders 12866 and 13563 direct agencies to assess all costs and benefits of available regulatory alternatives and, if regulation is necessary, to select regulatory approaches that maximize net benefits

(including potential economic, environmental, public health and safety effects, distributive impacts, and equity). E.O. 13563 emphasizes the importance of quantifying both costs and benefits, of reducing costs, of harmonizing rules, and of promoting flexibility.

This interim final rule has been determined to be a ''significant regulatory action'' under section 3(f) of E.O. 12866. The rulemaking is considered a deregulatory action because it removes a barrier to the manufacturing, labeling as NIOSH-approved, and selling of new PAPR designs intended for healthcare and other workplace settings. With the promulgation of the interim final requirements, manufacturers have a choice to submit approval applications under either the existing PAPR class HE standard or the new class PAPR100 standard.

The new PAPR100 respirators are required to meet most of the requirements and testing standards applied to class HE respirators except for the silica dust loading test in § 84.179, which requires that the device perform for a minimum service time of 4 hours. Three new requirements—a low-flow warning device (§ 84.171(j)), particulate loading test (§ 84.180), and communication performance testing (§ 84.181)—apply to class PAPR100 respirators only. HHS requests data that would facilitate quantification of: (a) The incremental cost savings resulting from the removal of the silica dust loading test requirements, and (b) the incremental costs resulting from each of the three new requirements.

This rule does not impose any mandatory costs on the public and benefits manufacturers who choose to develop a product under these new technical requirements. Healthcare facilities that currently utilize PAPR class HE devices that are designed for industrial use may also see a cost saving because class PAPR100 respirators designed for healthcare or other workplace settings may be more affordable than the current devices. In discussions with NIOSH, manufacturers have indicated that the cost of future class PAPR100 respirators is likely to be substantially less than the current cost of class HE devices. HHS requests data that would facilitate estimation of: (a) The increase in PAPR device availability resulting from this likely cost reduction, and (b) the timing of such availability relative to the issuance of this interim final rule.

HHS also requests data or other comment relevant to the benefits of this rulemaking—including, but not limited to, quantitative evidence on the duration of worker exposure to the hazards that class PAPR100 devices and other respirators protect against.

B. Regulatory Flexibility Act

The Regulatory Flexibility Act (RFA) does not apply to a rulemaking when a general notice of proposed rulemaking is not required. 5 U.S.C. 603 and 604. As noted previously, the Agencies have determined for good cause that it is impracticable and contrary to the public interest to publish a general notice of proposed rulemaking for this joint final rule. Accordingly, the RFA's requirements relating to an initial and final regulatory flexibility analysis do not apply.

C. Paperwork Reduction Act

The Paperwork Reduction Act (PRA), 44 U.S.C. 3501 *et seq.,* requires an agency to invite public comment on, and to obtain OMB approval of, any regulation that requires 10 or more people to report information to the agency or to keep certain records. The Office of Management and Budget (OMB) has already approved the information collection and recordkeeping requirements for certification and approval of respiratory protective devices under OMB Control Number 0920–0109, *Information Collection Provisions in 42 CFR part 84—Tests and Requirements for Certification and Approval of Respiratory Protective Devices* (expiration date April 30, 2021). Due to this interim final rule, which would allow for the NIOSH approval of respirators in a new class, PAPR100, there is likely to be a change in burden in the approved collection of information.

Based on PAPR activity over the last several years and also the increased number of related inquiries in response to the COVID–19 pandemic, NIOSH estimates that up to 5 respirator manufacturers may submit approximately 23 applications for PAPR100 approvals to the National Personal Protective Technology Laboratory from April 2020 through April 2021. Each application is expected to require an average of 229 hours to complete and maintain.

Accordingly, NIOSH expects 5,267 burden hours to be attributed to applications for PAPR100 approvals. NIOSH estimates an hourly wage rate of $79.89 (wage data is the average unspecified manufacturing industry engineer wage of $45.68 as reported in the 2016 National Sector NAICS Industry-Specific estimates multiplied by 1.06 inflation adjustment and 1.65 factor for overhead expenses).

Section	TitleC	Number of respondents	Average responses per respondent	Average burden per response (hr)	Total burden (hr)
§ 84.170	Air-purifying particulate respirators; description	5	4.6	229	5,267

Section	Title	Total burden hours (from above)	Estimated hourly wage rate	Total cost of hour burden
§ 84.170	Air-purifying particulate respirators; description	5,267	79.89	$420,780

The agency will submit the adjustment in burden for OMB Control No. 0920–0109 to OMB for its emergency review and approval.

D. Congressional Review Act

As required by Congress under the Congressional Review Act (5 U.S.C. 801 *et seq.*), HHS will report the promulgation of this rule to Congress prior to its effective date. This rule is not likely to result in an annual effect on the economy of $100,000,000 or more; a major increase in costs or prices for consumers, individual industries, Federal, State, or local government agencies, or geographic regions; or significant adverse effects on competition, employment, investment, productivity, innovation, or on the ability of U.S.-based enterprises to compete with foreign-based enterprises

in domestic and export markets. Pursuant to the Congressional Review Act (5 U.S.C. 801 *et seq.*), the Office of Information and Regulatory Affairs designated this rule as not a ''major rule,'' as defined by 5 U.S.C. 804(2).

E. Unfunded Mandates Reform Act of 1995

Title II of the Unfunded Mandates Reform Act of 1995 (2 U.S.C. 1531 *et seq.*) directs agencies to assess the effects of Federal regulatory actions on State, local, and Tribal governments, and the private sector ''other than to the extent that such regulations incorporate requirements specifically set forth in law.'' For purposes of the Unfunded Mandates Reform Act, this interim final rule does not include any Federal mandate that may result in increased annual expenditures in excess of $100 million by State, local, or Tribal governments in the aggregate, or by the private sector.

F. Executive Order 12988 (Civil Justice Reform)

This interim final rule has been drafted and reviewed in accordance with Executive Order 12988 and will not unduly burden the Federal court system. This rule has been reviewed carefully to eliminate drafting errors and ambiguities.

G. Executive Order 13132 (Federalism)

HHS has reviewed this interim final rule in accordance with Executive Order 13132 regarding federalism, and has determined that it does not have ''federalism implications.'' The rule does not ''have substantial direct effects on the States, on the relationship between the national government and the States, or on the distribution of power and responsibilities among the various levels of government.''

H. Executive Order 13045 (Protection of Children From Environmental Health Risks and Safety Risks)

In accordance with Executive Order 13045, HHS has evaluated the environmental health and safety effects of this interim final rule on children. HHS has determined that the rule would have no environmental health and safety effect on children.

I. Executive Order 13211 (Actions Concerning Regulations That Significantly Affect Energy Supply, Distribution, or Use)

In accordance with Executive Order 13211, HHS has evaluated the effects of this interim final rule on energy supply, distribution or use, and has determined that the rule will not have a significant adverse effect.

J. Plain Writing Act of 2010

Under Public Law 111–274 (October 13, 2010), executive Departments and Agencies are required to use plain language in documents that explain to the public how to comply with a requirement the Federal government administers or enforces. HHS has attempted to use plain language in promulgating the interim final rule consistent with the Federal Plain Writing Act guidelines but notes that these standards are technical in nature.

List of Subjects in 42 CFR Part 84

Mine safety and health, Occupational safety and health, Personal protective equipment, Respirators.

Final Rule

For the reasons discussed in the preamble, the Department of Health and Human Services amends 42 CFR part 84 as follows:

PART 84—APPROVAL OF RESPIRATORY PROTECTIVE DEVICES

■ 1. The authority citation for part 84 continues to read as follows:

Authority: 29 U.S.C. 651 *et seq.;* 30 U.S.C. 3, 5, 7, 811, 842(h), 844.

Subpart A—General Provisions

■ 2. Amend § 84.2 by adding definitions for ''Loose fitting'', ''Respiratory inlet covering'', ''Tight fitting'', and ''Warning device'' in alphabetical order to read as follows:

§ 84.2 Definitions.

* * * * *

Loose fitting means respiratory inlet covering that covers the wearer's head and neck, or head, neck, and shoulders, or whole body (when integral to the design).

* * * * *

Respiratory inlet covering means that portion of a respirator that forms the protective barrier between the user's respiratory tract and an air-purifying device or breathing air source, or both.

* * * * *

Tight fitting means a respiratory inlet covering that forms a complete gas tight or dust tight seal with the face or neck.

* * * * *

Warning device is a component of a respiratory protective device that informs the wearer to take some action.

Subpart G—General Construction and Performance Requirements

§ 84.60 [Amended]

■ 3. Amend § 84.60, in paragraph (a), by removing the words ''subparts H through KK'' and adding in their place the words ''subparts H through O''.

§ 84.63 [Amended]

■ 4. Amend § 84.63, paragraphs (a) through (c) by removing the words ''subparts H through KK'' and adding in their place the words ''subparts H through O''.

§ 84.64 [Amended]

■ 5. Amend § 84.64, in paragraph (b), by removing the words ''subparts H through KK'' and adding in their place the words ''subparts H through O''.

§ 84.65 [Amended]

■ 6. Amend § 84.65, in paragraph (a), by removing the words ''subparts H through KK'' and adding in their place the words ''subparts H through O''.

Subpart I—Gas Masks

§ 84.125 [Amended]

■ 7. Amend § 84.125 by removing the words ''§§ 84.170 through 84.183, except for the airflow resistance test of § 84.181'' and adding in their place the words ''§§ 84.170 through 84.181, except for the airflow resistance test of § 84.172''.

■ 8. Amend § 84.126 by adding paragraph (f) to read as follows:

§ 84.126 Canister bench tests; minimum requirements.

* * * * *

(f) Powered air-purifying respirators with a canister(s) and particulate filter(s) must meet the as-received minimum service-life requirements and half of the equilibrated minimum service-life requirements set forth in Tables 5, 6, and 7 of subpart I using the flows specified in subpart K, § 84.175(b) and equilibrated in accordance with paragraphs (a) through (e) of this section using the flows specified in subpart K, § 84.175(b).

Subpart L—Chemical Cartridge Respirators

§ 84.206 [Amended]

■ 9. Amend § 84.206, in paragraph (b), by removing the words ''§§ 84.179 through 84.183'' and adding in their place the words ''§§ 84.170 through 84.181''.

■ 10. Amend § 84.207 by adding paragraph (h) to read as follows:

§ 84.207 Bench tests; gas and vapor tests; minimum requirements; general.

* * * * *

(h) Powered air-purifying respirators with a cartridge(s) and particulate filter(s) must meet the as-received minimum service-life requirements and half of the equilibrated minimum service-life requirements set forth in table 11 of subpart L using the flows specified in subpart K, § 84.175(b) and equilibrated in accordance with paragraphs (a) through (g) of this section using the flows specified in subpart K, § 84.175(b).

■ 11. Subpart K is revised to read as follows:

Subpart K—Air-Purifying Particulate Respirators

Sec.
84.170 Air-purifying particulate respirators; description.
84.171 Required components and attributes.
84.172 Airflow resistance test.
84.173 Exhalation valve leakage test.
84.174 Filter efficiency level determination test—non-powered series N, R, and P filtration.
84.175 Instantaneous filter efficiency level determination test—PAPR series HE, PAPR100–N, and PAPR100–P filtration.
84.176 Fit test—PAPR classes HE and PAPR100.
84.177 Total noise level test—PAPR classes HE and PAPR100.
84.178 Breath response type, airflow resistance test—PAPR classes HE and PAPR100.
84.179 Silica dust loading test—PAPR series HE filtration.
84.180 Particulate loading test—PAPR series PAPR100–N and PAPR100–P filtration.
84.181 Communication performance test—PAPR class PAPR100.

Subpart K—Air-Purifying Particulate Respirators

§ 84.170 Air-purifying particulate respirators; description.

(a) *Non-powered air-purifying particulate respirators (series N, R, and P).* (1) Non-powered air-purifying particulate respirators utilize the wearer's negative inhalation pressure to draw the ambient air through the air-purifying filter elements (filters) to remove particulates from the ambient air. They are designed for use as respiratory protection against atmospheres with particulate contaminants at concentrations that are not immediately dangerous to life or health and that contain adequate oxygen to support life.

(2) Non-powered air-purifying particulate respirators are classified into three series, N-, R-, and P-series. The N-series filters are restricted to use in those workplaces free of oil aerosols. The R- and P-series filters are intended for removal of any particulate that includes oil-based liquid particulates.

(3) Non-powered air-purifying particulate respirators are classified according to the efficiency level of the filter(s) as tested according to the requirements of this part.

(i) N100, R100, and P100 filters must demonstrate a minimum efficiency level of 99.97 percent.

(ii) N99, R99, and P99 filters must demonstrate a minimum efficiency level of 99 percent.

(iii) N95, R95, and P95 filters must demonstrate a minimum efficiency level of 95 percent.

(b) *Powered air-purifying particulate respirators (PAPR classes HE and PAPR100).* (1) Powered air-purifying particulate respirators utilize a blower to move the ambient air through the air-purifying filter elements (filters) to remove particulate contaminants and deliver clean air to the respiratory inlet covering. They are designed for use as respiratory protection against atmospheres considered not immediately dangerous to life or health and that contain adequate oxygen to support life.

(2) Powered air-purifying particulate respirators are classified into two classes, HE and PAPR100, and three series, HE, PAPR100–N, and PAPR100–P. The N-series filters are restricted to use in those workplaces free of oil aerosols. The P-series filters are intended for removal of any particulate that includes oil-based liquid particulates.

(3) All three filter series, HE, PAPR100–N, and PAPR100–P, for powered air-purifying particulate respirators must demonstrate a minimum efficiency level of 99.97 percent.

§ 84.171 Required components and attributes.

The components of each air-purifying particulate respirator must meet the minimum construction requirements set forth in subpart G of this part. Each air-purifying particulate respirator described in § 84.170 must, where its design requires, contain the following component parts:

(a) *Respiratory inlet covering.* (1) Tight fitting respiratory inlet coverings must be designed and constructed to fit persons with various facial shapes and sizes either:

(i) By providing more than one size; or

(ii) By providing one size which will fit varying facial shapes and sizes.

(2) Full facepieces must provide for optional use of corrective spectacles or lenses, which must not reduce the respiratory protective qualities of the respirator.

(3) Loose fitting respiratory inlet coverings must be designed and constructed to fit persons with various head sizes, provide for the optional use of corrective spectacles or lenses, and insure against any restriction of movement by the wearer.

(4) Mouthpieces must be equipped with noseclips which are securely attached to the mouthpiece or respirator and provide an airtight seal.

(5) Respiratory inlet coverings that incorporate a lens or faceshield must be designed to prevent eyepiece fogging.

(6) Half-mask facepieces must not interfere with the fit of common industrial safety spectacles, including corrective safety spectacles.

(7) Respiratory inlet coverings must be designed and constructed to provide adequate vision which is not distorted by the eyepieces.

(b) *Filter unit.* The respirator manufacturer, as part of the application for certification, must specify the filter series and the filter efficiency level (*i.e.,* ''N95,'' ''R95,'' ''P95,'' ''N99,'' ''R99,'' ''P99,'' ''N100,'' ''R100,'' ''P100,'' ''HE,'' ''PAPR100–N'' or ''PAPR100–P'') for which certification is being sought.

(1) Filters for non-powered respirators (series N, R, and P) must be prominently labeled as follows:

(i) N100 filters must be labeled ''N100 Particulate Filter (99.97% filter efficiency level)'' and must be a color other than magenta.

(ii) R100 filters must be labeled ''R100 Particulate Filter (99.97% filter efficiency level)'' and must be a color other than magenta.

(iii) P100 filters must be labeled ''P100 Particulate Filter (99.97% filter efficiency level)'' and must be color coded magenta.

(iv) N99 filters must be labeled ''N99 Particulate Filter (99% filter efficiency level)'' and must be a color other than magenta.

(v) R99 filters must be labeled ''R99 Particulate Filter (99% filter efficiency level)'' and must be a color other than magenta.

(vi) P99 filters must be labeled ''P99 Particulate Filter (99% filter efficiency level)'' and must be a color other than magenta.

(vii) N95 filters must be labeled as ''N95 Particulate Filter (95% filter efficiency level)'' and must be a color other than magenta.

(viii) R95 filters must be labeled as ''R95 Particulate Filter (95% filter efficiency level)'' and must be a color other than magenta.

(ix) P95 filters must be labeled as ''P95 Particulate Filter (95% filter

efficiency level)'' and must be a color other than magenta.

(2) Filters for powered respirators (classes HE and PAPR100) must be prominently labeled as follows:

(i) HE filters must be labeled as ''HE Particulate Filter (99.97% filter efficiency level)'' and must be color coded magenta.

(ii) PAPR100–N filters must be labeled as ''PAPR100–N Particulate Filter (99.97% filter efficiency level)'' and must be color coded magenta.

(iii) PAPR100–P filters must be labeled as ''PAPR100–P Particulate Filter (99.97% filter efficiency level)'' and must be color coded magenta.

(c) *Valves.* (1) Inhalation and exhalation valves must be protected against distortion.

(2) Inhalation valves must be designed and constructed and provided where necessary to prevent excessive exhaled air from adversely affecting filters, except where filters are specifically designed to resist moisture.

(3) Exhalation valves must be:

(i) Provided where necessary;

(ii) Protected against damage and external influence; and

(iii) Designed and constructed to prevent inward leakage of contaminated air.

(d) *Head harness.* (1) All facepieces must be equipped with head harnesses designed and constructed to provide adequate tension during use and an even distribution of pressure over the entire area in contact with the face.

(2) Facepiece head harnesses, except those employed on filtering facepiece respirators, must be adjustable and replaceable.

(3) Mouthpieces must be equipped, where applicable, with adjustable and replaceable harnesses, designed and constructed to hold the mouthpiece in place.

(e) *Breathing tube.* Flexible breathing tubes used in conjunction with respirators must be designed and constructed to prevent:

(1) Restriction of free head movement;

(2) Disturbance of the fit of facepieces, mouthpieces, or loose fitting respiratory-inlet covering;

(3) Interference with the wearer's activities; and

(4) Shutoff of airflow due to kinking, or from chin or arm pressure.

(f) *Drink tube.* (1) For particulate respirators equipped with a drink tube, the respirator must meet all requirements of the standard with the drink tube in place.

(2) Dry drinking tube assembly will be subjected to a suction of 75 mm water column height while in a normal operating position (closed).

(3) Leakage through the drinking tube assembly must not exceed 30 mL per minute.

(g) *Container.* (1) Except as provided in paragraph (b) of this section, each respirator must be equipped with a substantial, durable container bearing markings which show the applicant's name, the type of respirator it contains, and all appropriate approval labels.

(2) Containers for respirators may provide for storage of more than one respirator; however, such containers must be designed and constructed to prevent contamination of respirators which are not removed, and to prevent damage to respirators during transit.

(h) *Harness.* (1) Each respirator must, where necessary, be equipped with a suitable harness designed and constructed to hold the components of the respirator in position against the wearer's body.

(2) Harnesses must be designed and constructed to permit easy removal and replacement of respirator parts, and, where applicable, provide for holding a full facepiece in the ready position when not in use.

(i) *Attached blower—PAPR classes HE and PAPR100.* Blowers must be designed to achieve the air flow rates required by the testing standards in § 84.175.

(j) *Low-flow warning device—PAPR class PAPR100.* (1) The design must include a low-flow warning. It must actively and readily indicate when flow inside the respiratory inlet covering falls below the minimum air flow defined in § 84.175.

(2) Any warning must be detectable by the wearer without any intervention by the wearer.

(3) Warning devices must be configured so that they may not be de-energized while the blower is energized.

(4) During use, warning devices must not switch off automatically and must not be capable of being switched off by the wearer.

(5) Any warnings which require different reactions by the wearer must be distinguishable from one another.

(6) If the warning provided is audible only, or other warnings are not readily apparent to the wearer, the minimum sound level must be 80 dBA.

§ 84.172 Airflow resistance test.

(a) Resistance to airflow will be measured in the tight-fitting respiratory inlet covering of a complete particulate respirator mounted on a test fixture with air flowing at continuous rate of 85 ±2 liters per minute, before each test conducted in accordance with § 84.173.

(b) Resistance of a complete tight-fitting powered air-purifying particulate respirator system will be measured with the blower off if the manufacturer indicates that the respirator should not be doffed in the event of a blower failure.

(c) The maximum allowable resistance requirements for air-purifying particulate respirators are as follows:

MAXIMUM RESISTANCE

[mm water-column height]

Respirator type	Inhalation		Exhalation
	Initial	Final	
Non-Powered (N, R, and P)	35	N/A	25
Powered (tight fitting) (HE class and PAPR100 class)	50	70	20

§ 84.173 Exhalation valve leakage test.

(a) Dry exhalation valves and valve seats will be subjected to a suction of 25 mm water-column height while in a normal operating position.

(b) Leakage between the valve and valve seat must not exceed 30 mL per minute.

§ 84.174 Filter efficiency level determination test—non-powered series N, R, and P filtration.

(a) Twenty filters of each non-powered air-purifying particulate respirator model will be tested for filter efficiency against:

(1) A solid sodium chloride particulate aerosol as per this section, if N-series certification is requested by the applicant.

(2) A dioctyl phthalate (DOP) or equivalent liquid particulate aerosol as per this section, if R-series or P-series

certification is requested by the applicant.

(b) Filters including holders and gaskets, when separable, will be tested for filter efficiency level, as mounted on a test fixture in the manner as used on the respirator.

(c) Prior to filter efficiency testing of 20 N-series filters, the 20 to be tested will be taken out of their packaging and placed in an environment of 85 ± 5 percent relative humidity at 38 ± 2.5 °C for 25 ±1 hours. Following the pre-conditioning, filters will be sealed in a gas-tight container and tested within 10 hours.

(d) When the filters do not have separable holders and gaskets, the exhalation valves will be blocked so as to ensure that leakage, if present, is not included in the filter efficiency level evaluation.

(e) For non-powered air-purifying particulate respirators with a single filter, filters will be tested at a continuous airflow rate of 85 ± 4 liters per minute. Where filters are to be used in pairs, the test-aerosol airflow rate will be 42.5 ± 2 liters per minute through each filter.

(f) Filter efficiency test aerosols:

(1) When testing N-series filters, a sodium chloride or equivalent solid aerosol at 25 ± 5 °C and relative humidity of 30 ± 10 percent that has been neutralized to the Boltzmann equilibrium state will be used. Each filter will be challenged with a concentration not exceeding 200 mg/m^3.

(2) When testing R-series and P-series filters, a neat cold-nebulized dioctyl phthalate (DOP) or equivalent aerosol at 25 ± 5 °C that has been neutralized to the Boltzmann equilibrium state will be used. Each filter will be challenged with a concentration not exceeding 200 mg/m^3.

(3) The test will continue until minimum efficiency is achieved or until an aerosol mass of at least 200 ± 5 mg has contacted the filter. For P-series filters, if the filter efficiency is decreasing when the 200 ± 5 mg challenge point is reached, the test will be continued until there is no further decrease in efficiency.

(g) The sodium chloride test aerosol will have a particle size distribution with count median diameter of 0.075 ± 0.020 µm and a standard geometric deviation not exceeding 1.86 at the specified test conditions as determined with a scanning mobility particle sizer or equivalent. The DOP aerosol will have a particle size distribution with count median diameter of 0.185 ± 0.020 µm and a standard geometric deviation not exceeding 1.60 at the specified test conditions as determined with a scanning mobility particle sizer or equivalent.

(h) The efficiency of the filter will be monitored and recorded throughout the test period by a suitable forward-light-scattering photometer or equivalent instrumentation.

(i) The minimum efficiency for each of the 20 filters will be determined and recorded and must be equal to or greater than the filter efficiency criterion listed for each level as follows:

Filter series	Efficiency (%)
P100, R100, N100	≥99.97
P99, R99, N99	≥99
P95, R95, N95	≥95

§ 84.175 Instantaneous filter efficiency level determination test—PAPR series HE, PAPR100–N, and PAPR100–P filtration.

(a) Three filters from each powered air-purifying particulate respirator for efficiency will be tested against a neat cold-nebulized dioctyl phthalate (DOP) or equivalent aerosol at 25 ± 5 °C that has been neutralized to the Boltzmann equilibrium state.

(b) Single air-purifying particulate respirator filter units will be tested in an atmosphere concentration of 100 mg/m^3 of DOP at the following continuous flow rates for a period of 5 to 10 seconds:

Type of respiratory inlet covering	Airflow rate (liters per minute)
Tight-fitting	115
Loose-fitting	170

(c) Powered air-purifying particulate respirators with multiple filter units will be tested by dividing the flow rate specified in paragraph (b) of this section by the total number of filters used.

(d) The filter will be mounted on a connector in the same manner as used on the respirator and the total efficiency must be ≥99.97 percent.

§ 84.176 Fit test—PAPR classes HE and PAPR100.

NIOSH will assess powered air-purifying respirator fit using either isoamyl acetate or generated aerosol.

(a) *Isoamyl acetate (IAA) fit test.* The applicant must provide a charcoal-filled canister or cartridge of a size and resistance similar to the filter unit with connectors which can be attached to the facepiece in the same manner as the filter unit.

(1) The canister or cartridge will be used in place of the filter unit, and persons will each wear a modified half-mask facepiece for 8 minutes in a test chamber containing 100 parts (by volume) of isoamyl acetate vapor per million parts of air.

(i) The following work schedule will be performed by each wearer in the test chamber:

(A) Two minutes nodding up and down, and turning head side to side; and

(B) Two minutes calisthenic arm movements.

(C) Two minutes running in place.

(D) Two minutes pumping with tire pump.

(ii) The facepiece must be capable of adjustment, according to the applicant's instructions, to each wearer's face, and the odor of isoamyl acetate must not be detectable by any wearer during the test.

(2) Where the respirator is equipped with a full facepiece, hood, helmet, or mouthpiece, the canister or cartridge will be used in place of the filter unit, and persons will each wear the modified respiratory inlet covering for 8 minutes in a test chamber containing 500 parts (by volume) of isoamyl acetate vapor per million parts of air, performing the work schedule specified in paragraph (b)(2) of this section.

(b) *Generated aerosol fit test.* The powered air-purifying particulate respirator system is tested in an atmosphere containing 20–40 mg/m^3 corn oil aerosol having a mass median aerodynamic diameter of 0.4 to 0.6 µm.

(1) The following activities will be performed by each wearer in the test chamber:

(i) Two minutes, nodding and turning head;

(ii) Two minutes, calisthenic arm movements;

(iii) Two minutes, running in place; and

(iv) Two minutes, pumping with a tire pump into a 28-liter (1 ft^3) container.

(2) The respiratory inlet covering will be adjusted, according to the applicant's instructions, to each wearer's face.

(3) The appropriate fit factor must be exceeded during the entire test.

§ 84.177 Total noise level test—PAPR classes HE and PAPR100.

Noise levels generated by any powered air-purifying respirators that cover the ears (*i.e.,* hood or helmet) will be measured at the entrance to each ear at maximum airflow obtainable and must not exceed 80 dBA.

§ 84.178 Breath response type, airflow resistance test—PAPR classes HE and PAPR100.

Resistance to airflow will be measured with a breathing machine as described in § 84.88.

(a) Minimum inhalation resistance must be greater than zero mm of water-column height.

(b) Maximum exhalation resistance must be less than 89 mm of water-column height.

§ 84.179 Silica dust loading test—PAPR series HE filtration.

(a) Three powered air-purifying particulate respirators will be tested for a period of 4 hours each at a flowrate not less than 115 liters per minute for tight-fitting facepieces, and not less than 170 liters per minute for loose-fitting hoods and helmets.

(b) The relative humidity in the test chamber will be 20–80 percent, and the room temperature approximately 25 °C.

(c) The test suspension in the chamber will not be less than 50 nor more than 60 mg of flint (99 + percent free silica) per m^3 of air.

(d) The flint in suspension will be 99 + percent through a 270-mesh sieve.

(e) The particle-size distribution of the test suspension will have a geometric mean of 0.4 to 0.6 µm and the standard geometric deviation will not exceed 2.

(f) The total amount of unretained test suspension in samples taken during testing must not exceed 14.4 mg for a powered air-purifying particulate respirator with tight-fitting facepiece, and 21.3 mg for a powered air-purifying particulate respirator with loose-fitting hood or helmet.

§ 84.180 Particulate loading test—PAPR series PAPR100–N and PAPR100–P filtration.

(a) Twenty filters of each powered air-purifying particulate respirator design will be tested for filter efficiency against:

(1) A solid sodium chloride particulate aerosol, in accordance with paragraph (d)(1) of this section, if series PAPR100–N approval is requested by the applicant.

(2) A dioctyl phthalate or equivalent liquid particulate aerosol, in accordance with paragraph (d)(2) of this section, if series PAPR100–P approval is requested by the applicant.

(b) Prior to filter efficiency testing of 20 series PAPR100–N filters, the 20 to be tested will be taken out of their packaging and placed in an environment of 85 ±5 percent relative humidity at 38 ±2.5 °C for 25 ±1 hours. Following the pre-conditioning, filters will be sealed in a gas-tight container and tested within 10 hours.

(c) For powered air-purifying particulate respirators with a single filter, filters will be tested at a continuous airflow rate of 85 ±4 liters per minute. Where filters are to be used in pairs, the test-aerosol airflow rate will be 42.5 ±2 liters per minute through each filter.

(d) Filter efficiency test aerosols:

(1) Series PAPR100–N filters:

(i) A sodium chloride or equivalent solid aerosol at 25 ±5 °C and relative humidity of 30 ±10 percent that has been neutralized to the Boltzmann equilibrium state will be used. Each filter will be challenged with a concentration not exceeding 200 mg/m^3.

(ii) The sodium chloride test aerosol will have a particle size distribution with count median diameter of 0.075 ±0.020 µm and a standard geometric deviation not exceeding 1.86 at the specified test conditions as determined with a scanning mobility particle sizer or equivalent.

(2) Series PAPR100–P filters:

(i) A neat cold-nebulized dioctyl phthalate (DOP) or equivalent aerosol at 25 ± 5 °C that has been neutralized to the Boltzmann equilibrium state will be used. Each filter will be challenged with a concentration not exceeding 200 mg/m^3.

(ii) The DOP aerosol shall have a particle size distribution with count median diameter of 0.185 ±0.020 µm and a standard geometric deviation not exceeding 1.60 at the specified test conditions as determined with a scanning mobility particle sizer or equivalent.

(e) The test will continue until minimum efficiency is achieved or until an aerosol mass of at least 200 ±5 mg has contacted the filter. For PAPR100–P series filters, if the filter efficiency is decreasing when the 200 ±5 mg challenge point is reached, the test will be continued until there is no further decrease in efficiency.

(f) The efficiency of the filter will be monitored and recorded throughout the test period by a suitable forward-light scattering photometer or equivalent instrumentation.

(g) The minimum efficiency for each of the 20 filters will be determined and recorded and must be equal to or greater than the filter efficiency criterion for PAPR100–N and PAPR100–P, efficiency ≥99.97 percent, pursuant to § 84.170(b).

§ 84.181 Communication performance test—PAPR class PAPR100.

(a) Powered air-purifying respirators must be designed to allow for proper communication while worn.

(b) A Modified Rhyme Test [7] will be used to test the wearer's ability to communicate efficiently.

(c) The communications requirement is met if the overall performance rating is greater than or equal to 70 percent.

[7] The Modified Rhyme Test is used in speech intelligibility experiments. *See https://www.nist.gov/ctl/pscr/modified-rhyme-test-audio-library.*

Subpart KK [Removed]

■ 12. Subpart KK, consisting of §§ 84.1100 through 84.1158 and the tables, is removed.

Dated: April 7, 2020.

Eric D. Hargan,

Deputy Secretary, Department of Health and Human Services.

[FR Doc. 2020–07804 Filed 4–9–20; 4:15 pm]

BILLING CODE 4150–18–P

DEPARTMENT OF COMMERCE

National Oceanic and Atmospheric Administration

50 CFR Part 622

[Docket No. 200401–0096]

RIN 0648–BJ08

Fisheries of the Caribbean, Gulf of Mexico, and South Atlantic; Reef Fish Fishery of the Gulf of Mexico; Greater Amberjack Management Measures

AGENCY: National Marine Fisheries Service (NMFS), National Oceanic and Atmospheric Administration (NOAA), Commerce.

ACTION: Final rule.

SUMMARY: NMFS issues regulations to implement management measures described in a framework action to the Fishery Management Plan for the Reef Fish Resources of the Gulf of Mexico (FMP). This final rule revises the commercial trip limit in the Gulf of Mexico (Gulf) exclusive economic zone (EEZ) for greater amberjack. In addition, this final rule revises the boundaries of several Gulf reef fish management areas to reflect a change in the seaward boundary of Louisiana, Mississippi, and Alabama. The purpose of this final rule is to extend the commercial fishing season for greater amberjack by constraining the harvest rate while continuing to prevent overfishing and rebuild the stock in the Gulf, and to update the boundaries of reef fish management areas to reflect the current state water's boundaries for reef fish management.

DATES: This final rule is effective on May 14, 2020.

ADDRESSES: Electronic copies of the framework action, which includes an environmental assessment, a regulatory impact review, and a Regulatory Flexibility Act (RFA) analysis may be obtained from the Southeast Regional Office website at *https://www.fisheries.noaa.gov/action/framework-action-greater-amberjack-commercial-trip-limits.*
