# Supplementary material for: De Novo Powered Air-Purifying Respirator Design and Fabrication for Pandemic Response
Source: Front Bioeng Biotechnol. 2021 Sep 6;9:690905. doi: 10.3389/fbioe.2021.690905 (PMC8450396; doi:10.3389/fbioe.2021.690905)
Supplement: Supplementary file 1 [file DataSheet1.ZIP › Additional Materials/Supplementary Material 4/Bill of Materials/Commercial Design.pdf]

Bill of Materials for PanFab Commercial PAPR Design

Akshay Kothakonda[1,2,*], Lyla Atta[1,3,*], Deborah Plana[1,4,5,*], Ferrous Ward[1,2,*], Chris Davis[1,6], Avilash Cramer[1,5], Robert Moran[1,7], Jacob Freake[1,8], Enze Tian[1,9], Ofer Mazor[1,10], Pavel Gorelik[1,10], Christopher Van[1,11], Christopher Hansen[1,12], Helen Yang[1,13], Yao Li[1,14], Michael S. Sinha[1,13], Ju Li[1,14], Sherry H. Yu[1,15], Nicole R. LeBoeuf[1,16,†], Peter K. Sorger[1,4,†,‡]

[1]Greater Boston Pandemic Fabrication Team (PanFab) c/o Harvard-MIT Center for Regulatory Science, Harvard Medical School, Boston, MA, USA

[2]Department of Aeronautics and Astronautics, MIT, Cambridge, MA, USA

[3]Johns Hopkins University School of Medicine, Baltimore, MD, USA

[4]Harvard Ludwig Cancer Research Center and Department of Systems Biology, Harvard Medical School, Boston, MA, USA

[5]Harvard-MIT Division of Health Sciences & Technology, Cambridge, MA, USA

[6]GenOne Technologies, Cambridge, MA, USA

[7]Mine Survival, Panama City Beach, FL, USA

[8]Fikst Product Development, Woburn, MA, USA

[9]Beijing Key Laboratory of Indoor Air Quality Evaluation and Control, Department of Building Science, Tsinghua University, Beijing, China

[10]Research Instrumentation Core Facility, Harvard Medical School, Boston, MA, USA

[11]Borobot, Middleborough, MA, USA

[12]Harvard Graduate School of Design, Cambridge, MA, USA

[13]Harvard-MIT Center for Regulatory Science, Harvard Medical School, Boston MA, USA

[14]Department of Nuclear Science and Engineering and Department of Materials Science and Engineering, MIT, Cambridge, MA, USA

[15]Department of Dermatology, Yale School of Medicine, New Haven, CT USA

[16]Department of Dermatology, Center for Cutaneous Oncology, Brigham and Women's Hospital and Dana-Farber Cancer Institute, Boston, MA, USA

[*]These authors contributed equally to this work

[†]Co-corresponding authors. E-mails: nleboeuf@bwh.harvard.edu; peter_sorger@hms.harvard.edu cc: Maureen_Bergeron@hms.harvard.edu

[‡]Lead contact

© President and Fellows of Harvard College and The Massachusetts Institute of Technology, 2020.

ORCID IDs:
Akshay Kothakonda, 0000-0001-5424-4228
Lyla Atta, 0000-0002-6113-0082
Deborah Plana, 0000-0002-4218-1693
Avilash Cramer, 0000-0003-0014-8921
Jacob Freake, 0000-0002-5198-835X
Enze Tian, 0000-0001-6410-5360
Christopher Van, 0000-0003-3262-964X
Christopher Hansen, 0000-0002-6640-2745
Helen Yang, 0000-0002-9455-5300
Michael S. Sinha 0000-0002-9165-8611
Ju Li, PhD, 0000-0002-7841-8058
Sherry H. Yu: 0000-0002-1432-9128
Nicole R. LeBoeuf, MD, MPH, 0000-0002-8264-834X
Peter Sorger, PhD, 0000-0002-3364-1838

Parts used in the PanFab Commercial PAPR Design. The estimated costs assume a total production run of 2000 units.

S. No.	Item	Qty.	Supplier/Method	Part No.	Estimated cost ($)
1	Enclosure	1	Pelican	V100 Vault	40
2	Threaded Inserts for Filter	2	ABS Molding	N/A	6 (Tooling Cost = 6645)
3	Threaded Insert for Hose	1	ABS Molding	N/A	2.80 (Tooling Cost = 6846)
4	Blower, Centrifugal	1	Delta Electronics	BFB1012HD-04D4L	36
5	Battery pack, 12V NiMH	1	Tenergy	Amazon Standard Identification Number: B077Y9HNTF	23
6	Controller	1	Arduino	R3	23
7	PCB Shield	1	OSH Park	N/A	9.50
8	Differential Pressure Sensor	1	Sensirion	SDP810-500PA	19
9	Buzzer	1	Mallory Sonalert Products	PS-580Q	5
10	Potentiometer	1	Bourns Inc.	93R1A-R22-A12L	3

© President and Fellows of Harvard College and The Massachusetts Institute of Technology, 2020.

11	Transistor	1	ON Semiconductor	2N3904BU	0.20
12	Resistor	1	Vishay BC Semiconductor	PR02000201001JR500	0.32
13	Electrical Connector	1	TE Connectivity AMP Connectors	1-2834184-3	1.40
14	Venturi	1	BPE Inc.	178-71-2	0.54
15	Venturi Ports	2	Car-Anth Manufacturing	16-1204-2	0.68 (Tooling Cost = 375)
16	Blower Silicone Tube	1	McMaster-Carr	3038K29	N/A
17	Venturi Silicone Tube	2	McMaster-Carr	3038K12	N/A
18	Blower Adapter	1	ABS 3D Printing	N/A	6
20	Switch	1	McMaster-Carr	8002K114	28
21	Switch Cover	1	McMaster-Carr	70205K4	4.40
22	Gaskets for Threaded Connections	4	McMaster-Carr	5647K62	3
23	Waist Strap	1	Skil-Care	PathoShield Gait Belt	11
24	Filter	2	Milwaukee	49-90-1900	40
25	Filter Adapter	2	ABS Injection molding	N/A	6 (Tooling Cost = 6846)
26	Filter Cover	2	ABS Injection molding	N/A	8 (Tooling Cost = 18345)
27	Hose Adapter	2	ABS Injection Molding	N/A	6.60 (Tooling Cost = 9997)
28	Hose	1	Flexaust	Flex-Tube PU-IH, PN: 33800125000	8.50
29	Hood Coupler	1	ABS Injection molding	N/A	3.30 (Tooling Cost = 7456)
30	Locking Ring	1	ABS Injection	N/A	2.70

© President and Fellows of Harvard College and The Massachusetts Institute of Technology, 2020.

			Molding		(Tooling Cost = 5156)
31	Hood	1	University of Washington	VHA ADAPT PAPR Hood	30
**Total**					**327.94**

Notes:
1. Several costs are unofficial quotes.
2. Tooling cost for machining the Pelican case and joining the threaded inserts (Items 2 and 3) are TBD.

© President and Fellows of Harvard College and The Massachusetts Institute of Technology, 2020.
